# Revealing the Principle of Progressively Enhanced Photocatalytic Reactivity in Dual Single‐Atoms‐Mediated Electronic Interactions Optimization of Cd/Te‐TiO_2_

**DOI:** 10.1002/advs.202413379

**Published:** 2025-03-17

**Authors:** Yihang Zhang, Hao Zhao, Shan Jiang, Yanrong Zhang, Yong Chen, Jianyu Gong

**Affiliations:** ^1^ Hubei Key Laboratory of Multi‐media Pollution Cooperative Control in Yangtze Basin School of Environmental Science & Engineering Huazhong University of Science and Technology (HUST) 1037 Luoyu Road Wuhan Hubei 430074 China

**Keywords:** Cd/Te, dual‐single atoms, electronic interactions, photocatalytic activity, TiO2 nanotube

## Abstract

In this work, a CdTe@TiO_2_ single atoms (SAs) catalysts is successfully synthesized, realizing unique portion of nonbonding oxygen‐coordinated configuration of Cd─O─Te dimers coupling. Astonishingly, the 5th CdTe@TiO_2_ (0.027 min^−1^) shows progressively augmenting phenomenon, accompanied with 2.73 times higher than that of fresh CdTe@TiO_2_ (0.010 min^−1^) on the photocatalytic rate constant of gaseous toluene conversion. The incrementally enhanced photocatalytic activity is attributed to atomically dispersed Cd/Te SAs sites generation during the photoreduction process, and further leading to the optimized electron interactions between Cd, Te atoms, and TiO_2_ NTs and causing a positive shift in the d‐band center closer to the Fermi level. Density Functional Theory (DFT) calculations reveal that this unique Cd/Te SAs increasing phenomenon can mutually elevate the electronic density around Cd/Te SAs and generate a substantial local electric field at the interface. In essence, the free energy barriers of the benzene intermediates ring‐opening as the rate‐determining step appeared to significantly diminish tendency from 1.10 to 0.96 eV, in line with the ICOHP calculation of Cd/Te─O bonds in TS promoted from −2.43 to −3.49 eV. This work unearths the mechanism for ascendant electronic states of synergies dual‐metal sites, providing a versatile strategy to tailor the SAs catalysts for solar energy conversion.

## Graphical abstract

The incrementally enhanced photocatalytic reactivity is attributed to persistent in situ spontaneous growing of atomically dispersed Cd/Te SAs. Consequently, it mutually elevates the electronic density around Cd/Te SAs and strengthens interactions and binding capacity between occupied d orbitals at Cd/Te SAs and empty p orbits in TS. In essence, the free energy barriers of the benzene intermediates ring‐opening as the rate‐determining step appeared to significantly diminish tendency.

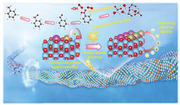

## 1 Introduction

Single‐atom catalysts have raised considerable attention owing to their exceptional catalytic performance and distinctive electronic structure.^[^ ^1^, ^2^ ^]^ It presented herein possess active sites that actively attract reactants, effectively lower the energy barrier, and facilitate the efficient transfer of photogenerated charges, and thereby, exhibiting exceptional co‐catalytic properties for pollutant conversion deriving from photoelectron transfer from the photocatalysts.^[^ ^3^ ^]^ It is well known that with decreasing the dimensions of metal clusters, there is an increase in the ratio of surface atoms involved in redox reactions, realizing exclusively optimal utilization of atoms for SAs catalysts.^[^ ^4^, ^5^ ^]^ Alternative approaches have been extensively investigated in the manipulation of SAs anchored on support composites. Notably, metal oxides exhibit a higher affinity toward reactant molecules even compared to carbon‐based counterparts, and thereby, offering potential for enhancing photocatalytic performance through the incorporation of isolated metal atoms.^[^ ^6^, ^7^, ^8^ ^]^

Disappointingly, the incidental instability and deactivation characteristics of SAs catalysts during sequential photo‐oxidation period still cannot be properly resolved, leading to unpromising application prospect in decontamination of industrially degradation‐resistant gaseous pollutants.^[^ ^9^ ^]^ Given by this, it is imperative to cultivate enduring and steadfast strategy for SAs catalysts design. Previous studies pointed out that the incorporation of metals, particularly those possessing noble properties, has frequently been employed to enhance the electron density on the surface of photocatalysts.^[^ ^10^, ^11^, ^12^ ^]^ It suggests that metals have been commonly introduced to enhance the surface electron density of photocatalysts.^[^ ^13^, ^14^, ^15^ ^]^ However, the regrettable stability of SAs composed of noble metals and the delicate nature of coordination bonds between noble metals and neighboring atoms, conversely, significantly cause adverse effects on the catalytic activity at these metal sites.^[^ ^16^, ^17^ ^]^

Therefore, the efforts directed toward atomically dispersed non‐noble metals SAs catalysts has received significant attention and investment, with the aim of reducing reliance on noble metals while preserving their exceptional catalytic activity. Corresponding, the non‐noble metals Cd/Te SAs catalysts are in favor of facilitating the photo‐oxidation activity evidenced by the heightened adsorption of reactants and the consequent redistribution of charges induced. Moreover, the impact of the metal center and its local coordination on photocatalytic performance is widely recognized.^[^ ^17^, ^18^, ^19^ ^]^ Given that the introduction of Cd/Te metal SAs results in redistribution of electron density, leading to electron accumulation around the metal and its surrounding atoms, considerable attention has been given to modifying the metal center with the aim of achieving enhanced photocatalytic activity. Zhang et al.^[^ ^20^ ^]^ reported a non‐noble metal Ni SAs catalysts for highly efficient synthesis of H_2_O_2_ in pure water, realizing an extraordinary apparent quantum yield and impressive solar‐to‐chemical conversion efficiency of 0.82%. Therefore, extensive research has revealed that the specific local coordination regulation in the non‐noble metals SAs catalysts plays a crucial role in determining the efficiency of electron transfer.^[^ ^20^, ^21^, ^22^, ^23^ ^]^

On the other hand, the support materials hold equal significance based on the pivotal role enacted by metal‐support interactions in the performance of metal SAs catalysts.^[^ ^24^, ^25^ ^]^ The lone‐pair electrons existing in oxygen atoms of TiO_2_, which can act as optimal locations for stabilizing metal SAs within its structural nanotubes, ensuring the formation of distinctive coordination structures that effectively regulate the electronic configuration and proffer desirable active sites through interaction between the metal SAs center and carrier.^[^ ^26^, ^27^, ^28^ ^]^ Moreover, the localized polarization state of host materials could be regulated by the reasonable coordination environment of SAs, and thereby, enabling manipulation of the charge transfer behavior.^[^ ^29^, ^30^ ^]^ The intrinsic activity of metal oxide‐loaded SAs catalysts could be significantly enhanced by optimizing the carrier properties, in this regard, creating new chemical bonds on the carrier to remotely influence the electronic structure of the metal center.^[^ ^31^, ^32^ ^]^

The dynamic structural evolution of the atomically dispersed metal catalytic sites and corresponding adsorbed chemical bonds variation in photocatalytic degradation under practical reaction conditions remain unclear,^[^ ^33^, ^34^ ^]^ severely limiting the further design and development of highly active photocatalysts system.^[^ ^35^ ^]^ Therefore, in our study presented, the novel SAs catalysts with Cd‐Te dual‐metal sites on TiO_2_ NTs for realizing miraculously progressive photocatalysis is successfully designed, and we utilize the gaseous toluene decontamination performance as the evaluation criteria for investigating the photocatalytic activity. And more importantly, Cd and Te dual SAs are synchronously in situ co‐anchored on TiO_2_ NTs with deactivation property under the action of photoreduction during continuous photocatalytic process. We deeply explored the mechanism of enduring formation of atomically dispersed Cd/Te catalytic sites for altering localized electronic configuration and interactions surrounding Cd/Te SAs, and thereby strengthening in‐plane polarization.

## 2 Results and Discussion

**Figure 1:**
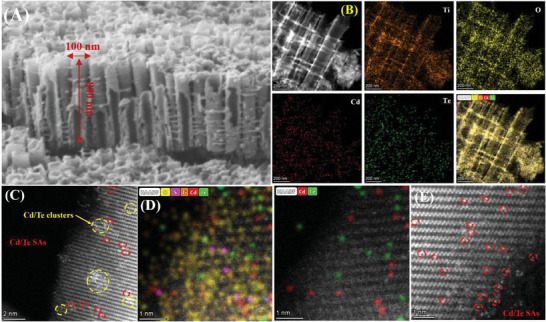
A) SEM image, B) Energy dispersive X‐ray spectra, and C) HAADF‐STEM image of the fresh CdTe@TiO_2_ NTs, D) HAADF‐STEM with energy dispersive X‐ray spectra of the fresh CdTe@TiO_2_ NTs on the scale of 1 nm, E) HAADF‐STEM image of the 5th CdTe@TiO_2_ NTs (single atomic Cd/Te sites high‐lighted by red circles).

In the anchoring procedure, a certain density of Cd atoms and/or Te atoms can be individually anchored as monodispersed sites on TiO_2_ NTs substrate for realizing the anticipated CdTe@TiO_2_ NTs assembling. The representative NTs morphology and microstructure in the TiO_2_ NTs and CdTe@TiO_2_ NTs were investigated through utilizing scanning electron microscope (SEM) and transmission electron microscopy (TEM) images (**Figure** **1**A,B; Figures S1–S3, Supporting Information). The TiO_2_ NTs were mainly fabricated with highly ordered long tubes with the roughly 100 nm diameter and 800 nm length of uniform size distribution. The unique high length‐to‐diameter fabrication yielded advantages to enhancing charge transfer,^[^ ^36^ ^]^ attributing to the selected electrolyte of glycerin could adjust local concentration fluctuations to the weak chemical dissolution of barrier layers, and thereby, resulting in the establishment of smooth NTs.^[^ ^36^ ^]^ The stable and high‐purity CdTe@TiO_2_ NTs crystalline structure was further verified from the nearly identical X‐ray diffraction (XRD) pattern in Figure S4 (Supporting Information). Notably, no sharpness of crystalline diffraction peaks for Cd and Te were observed, demonstrating the uniform dispersion of Cd and Te on TiO_2_ NTs surface and no periodic structure and crystalline clusters were arised on TiO_2_ NTs surface.^[^ ^37^ ^]^ The characteristic lattice fringes of the CdTe@TiO_2_ NTs were clearly identified through HRTEM images, as probed in Figure S5 (Supporting Information), the interplanar distances of 3.52 Å was determined for TiO_2_ NTs along the (101) crystallographic plane. Moreover, in order to optimize the atomic resolution for verifying the dispersions of Cd and Te sites on TiO_2_ NTs, the high‐angle annular dark‐field scanning transmission electron microscopy (HAADF‐STEM) equipped with a probe aberration‐corrector was adopted. Specifically, the bright spots labeled with red circles were recognized Cd/Te metals position mainly as SAs states dispersed in TiO_2_ NTs substrate as shown in Figure 1C. To further acquire the relative localizations of Cd and Te atoms, HAADF‐STEM analysis with energy dispersive X‐ray spectra were performed in Figure 1D and Figure S6 (Supporting Information), and thereby confirming the certain Cd and Te atoms were observed to be in associated proximity to each other spatially and anchored in isolated atoms patterns. Inspiringly, the adjacent Cd and Te SAs could produce a synergistic effect to rearrange electrons density around Cd and Te SAs.^[^ ^38^ ^]^

The pristine tubular architecture of the CdTe@TiO_2_ NTs remained intact experiencing photocatalytic process (Figure S7, Supporting Information), and it still exhibited homogeneously distributed arrangement of Ti, O, Cd and Te atoms throughout the CdTe@TiO_2_ NTs in terms of EDS in Figure S8 (Supporting Information) and HADDF‐STEM with EDX in Figure S9 (Supporting Information). Astonishingly, in HAADF‐STEM image of the 5th CdTe@TiO_2_ NTs, more Cd/Te SAs could be clearly observed, and the isolated bright dots circled in red in Figure 1E and Figure S10 (Supporting Information) unambiguously implied the atomically dispersed Cd and Te in the recycled CdTe@TiO_2_ NTs. In contrast, minimum Cd/Te SAs in the CdTe@TiO_2_ NTs could be meticulously monitored. Therefore, it could speculate that Cd/Te were partially in situ dissociated into the single sites throughout persistent photo‐reduction process during the continuous catalytic period, promoting the Cd and Te dual SAs sites quantity in the recycled CdTe@TiO_2_ NTs.^[^ ^39^, ^40^ ^]^ Correspondingly, the Cd/Te SAs quantity evolved from 10 atoms/nm^2^ (fresh CdTe@TiO_2_) to 22.6 atoms/nm^2^ (5th CdTe@TiO_2_) with photo‐reduction proceeding.^[^ ^38^ ^]^ Combining with the HAADF‐STEM analysis with energy dispersive X‐ray spectra in Figure S11 (Supporting Information), more highly dispersed Cd and Te dual SAs species were assembled on the 5th CdTe@TiO_2_ NTs compared with fresh CdTe@TiO_2_ NTs. Namely, Cd/Te dual SAs were generated in situ under the action of photoreduction during photocatalytic recycling degradation period, realizing the optimal coexistence of Cd and Te dual SAs sites in the CdTe@TiO_2_ NTs. Therefore, the sharply improving photocatalysis attained in the CdTe@TiO_2_ NTs could be potentially ascribed to synergistic impacts over the Cd and Te dual SAs sites, signaling cooperation on atomic level in this dual metal doped TiO_2_ support.

**Figure 2:**
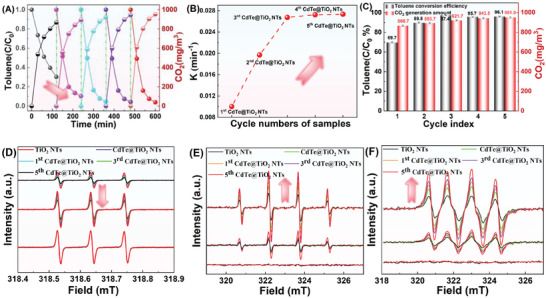
A) Photocatalytic performance for five‐run recycling test of toluene degradation curve on the CdTe@TiO_2_ NTs, B) Photocatalytic reaction rate constant, and C) Toluene conversion efficiency and CO_2_ generation amount of the CdTe@TiO_2_ NTs with various recycling times, EPR spectra of D) TEMPO‐h^+^, E) DMPO‐·OH, F) DMPO‐·O_2_^−^ for the TiO_2_ NTs, CdTe@TiO_2_ NTs, and recycled CdTe@TiO_2_ NTs under dark and light condition.

The photocatalytic activity was explored toward cyclic degradation of gaseous toluene under simulated solar light illumination. Only 30% degradation efficiency of toluene was reached over TiO_2_ NTs in 2 h irradiation during all repeated treatments (Figure S12, Supporting Information). Interestingly, after decorating Cd/Te SAs on the TiO_2_ NTs, CdTe@TiO_2_ NTs achieved 67% at the 1st cycle, and further sharply increased to 90% and 95% at the 2nd and 3rd time of recycling displayed in **Figure** **2**A. Furthermore, the optimized photocatalytic efficiency was noticed with the 4th and 5th CdTe@TiO_2_ NTs, which achieved complete degradation of gaseous toluene. The reaction kinetic curves depicted a rising pattern in the photocatalytic conversion rate as the recycling times further increasing to the 5th CdTe@TiO_2_ NTs (0.02734 min^−1^) (Figure 2B), manifesting 2.73 times higher than that of the CdTe@TiO_2_ NTs (0.01 min^−1^), and thus demonstrated that the multiple recycling period play a pivotal role in regulating the photocatalytic capacity of the CdTe@TiO_2_ NTs. Correspondingly, CO_2_ yield converted from toluene in the 5th CdTe@TiO_2_ NTs also achieved the highest value (Figure 2C). To exclude the potential influence of collaborative Cd and Te on photocatalytic performance, the photocatalytic property of individual Cd or Te SAs anchored on TiO_2_ NTs was investigated. Besides, the residual sulfur atoms participation could have the slight but negligible influence on the promoting photocatalysis, confirmed in Figure S13 (Supporting Information). It concluded that sole Cd or Te SAs sites existing would also favor the persistent enhancing photocatalytic activity (Figure S14A,B, Supporting Information). It still demonstrated that the CdTe@TiO_2_ NTs showed progressively enhancing photocatalytic activity with the cyclic degradation trials proceeding in Figure S14C (Supporting Information). Given by this, the synergistic effect on lowing activation energy of ring‐opening period of Cd and Te SAs existing would account for sharping enhancing toluene conversion efficiency.

In Figure 2D, it displayed a gradually depletion in 2,2,6,6‐tetramethylpiperidinyl‐1‐oxide (TEMPO) signal intensity with recycling times increasing, implying that persistent escalating of anchored Cd/Te dual SAs could facilitate carriers separation and generate more photo‐generated holes. As demonstrated in Figure 2E,F, under simulated light illumination, the 5th CdTe@TiO_2_ NTs performed the most intensitive ·OH and ·O_2_^−^ signals, evidencing more electrons could be transferred to surface for initiating ·OH and ·O_2_^−^ generation. This, therefore, implies more Cd─Te coordination sites could optimize the enrichment of photo‐generated charge around Cd/Te SAs and then exhibit exceptional abilities in lowering activation energy of photocatalytic conversion, engendering numerous active species.

The chemical state variation of Cd 3p, Te 3d, O 1s, and Ti 2p, evaluated from the corresponding XPS spectra, were presented in Figure S15 (Supporting Information). For fresh CdTe@TiO_2_ NTs, it revealed that Ti 2p peaks all had two peaks representing Ti 2p_3/2_ and Ti 2p_1/2_. In addition, O 1s possessed an asymmetric peak that could be fitted by two peaks, one had a lower binding energy implied the oxygen atoms in TiO_2_ lattice, while the other represented the surface ─OH.^[^ ^39^, ^40^ ^]^ Besides, the two main asymmetric peaks were observed at 405.7 and 412.3 eV of the CdTe@TiO_2_ NTs, indicating Cd 3d_5/2_ and Cd 3d_3/2_ states respectively. Te 3d_5/2_ and 3d_3/2_ at 576.7 and 587.1 eV of the CdTe@TiO_2_ was also presented. Notably, in 3rd CdTe@TiO_2_ NTs, the binding energies of inter Ti 2p, O 1s, Cd 3d, and Te 3d peaks all presented a shift to lower binding energy compared to that of the fresh CdTe@TiO_2_ NTs, manifesting valence state of Cd/Te metal tended to develop into 0 valence. It attributed to the synergy of persistent increasing of Cd─Te dual SAs originated from under light illumination.

**Figure 3:**
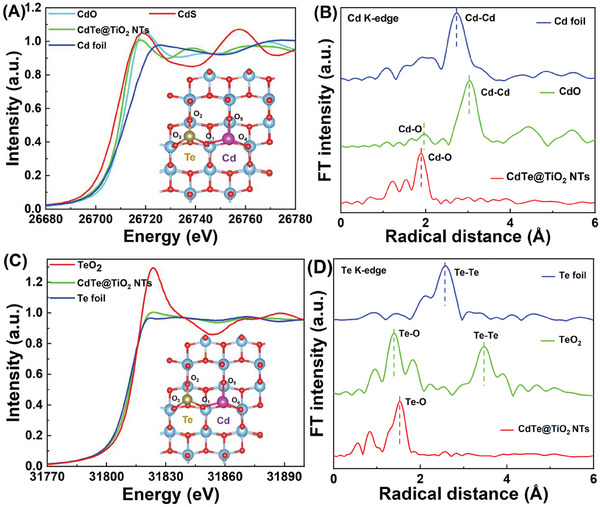
Atomic structure and coordination state investigation. A) Normalized XANES spectra, B) Fourier transform EXAFS spectra at the Cd K‐edge of the recycled CdTe@TiO_2_ NTs and reference; C) Normalized XANES, D) EXAFS spectra at the Te K‐edge of the recycled CdTe@TiO_2_ NTs and reference.

To further realize the valence states and coordination environment of the Cd─Te dual SAs sites at the atomic level, X‐ray absorption near‐edge structure (XANES) and extended X‐ray absorption fine structure (EXAFS) analysis were carried out. The XANES of Cd K‐edge (**Figure** **3**A) confirmed that the center peak energy was between that of Cd foil and CdO, hinting at the Cd oxidation state, ranging from 0 to 2+. This was attributed to the potent electric interaction between Cd, Te SAs, and TiO_2_, which was consistent with the XPS results. It manifested that the k^3−^ weighted Fourier‐transform Cd K‐edge EXAFS spectra (FT‐EXAFS) of Cd─Te dual SAs catalysts in Figure 3B. It exhibited the main peaks at 1.9 Å corresponding to Cd─O coordination peak, which was approaching but not identical as the Cd‐O bond nearest length in CdO crystalline, suggesting the distorted Cd─O coordination structure formation. Notably, faint Cd‐Cd coordination peak (2.8 Å) was perceived, materializing the occurrence of slight Cd SAs aggregation state. Moreover, it vividly depicted the k‐space of maximum intensity of roughly 5 Å^−1^ and R‐space value with 1.9 Å in the originating from the Cd─O coordination in the wavelet transform (WT) profiles, exhibiting the distinction from the signals emitted by Cd foil and CdO (Figure S16A, Supporting Information). Similarly, Te atoms presented the valence state of partially positive between 0 and 4+ compared to the XANES spectra of Te foil and TeO_2_ at Te K‐edge (Figure 3C). Corresponding, the presence of scattering of Te─O coordination and the absence of Te─Te coordination suggested that Te species were not existing as metallic Te nanoparticles or clusters, but primarily presented as isolated atom (Figure 3D). The coordination characteristics of Te─O and Te─Te bonds were vividly demonstrated in the WT contour plots (Figure S16B, Supporting Information). The dominant peak at 1.7 Å was ascribed to the scattering interactions between the Te SAs and the first coordination shell (Te─O), with the approximate coordination number of four.^[^ ^41^ ^]^ The presence of isolated Cd and Te atomic sites suggested that they were uniformly distributed at an atomic level and fractionally interacted with O atoms without noticeable aggregation.^[^ ^42^ ^]^

**Figure 4:**
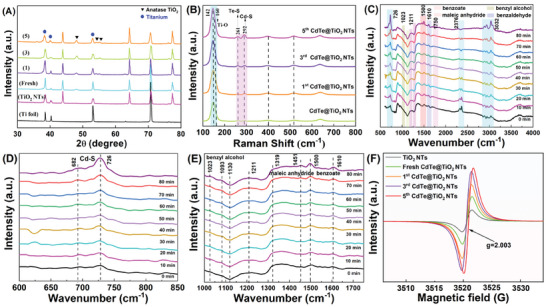
A) XRD patterns and B) Raman spectra of as‐synthesized TiO_2_ NTs, CdTe@TiO_2_ NTs, and recycled CdTe@TiO_2_ NTs, C–E) In situ DRIFTS measurement of gaseous toluene in PC reaction on the 1st CdTe@TiO_2_ NTs under visible light illumination (λ = 420 nm), F) EPR spectra for the TiO_2_ NTs, CdTe@TiO_2_ NTs and recycled CdTe@TiO_2_ NTs.

**Figure** **4**A shows the X‐Ray Diffraction (XRD) patterns of the recycled CdTe@TiO_2_ NTs, revealing no notable changes in the structure of crystals.^[^ ^43^ ^]^ Moreover, given its exceptional sensitivity toward microstructural variations, Raman spectroscopy was employed to investigate the chemical bonds variation related with sulfur atoms from electrolyte. As depicted in Figure 4B and Figure S17A (Supporting Information), the prominent and distinct peak observed at a frequency of 142 cm^−1^ corresponds to the E_g_ (1) mode of anatase TiO_2_, while the less pronounced and broader peak detected at 396 cm^−1^ could be attributed to the B1g(1)/A1_g_ modes. The presence of a gradually prominent peak at 261 cm^−1^ suggested the formation of interactions between Te ions and epibiotic S adsorbed in photocatalysts, indicating the active participation of Te‐S LO mode (Figure S17B, Supporting Information). The observed persistently remarkable redshift of Cd─S LO phonon at 293 cm^−1^, in comparison to the reported bulk value (314 cm^−1^), also signified that a distinct Te─S LO phonon existing in the recycled CdTe@TiO_2_ NTs.^[^ ^44^ ^]^ Given by this, it was speculated that the sulfur related bonds could be progressively generated under the light irradiation, originating from remaining sulfate radical adsorbed in photocatalysts surface of electrolyte after electrochemical deposition. Remarkably, the prominence of these peaks slightly intensified as the duration of visible‐light irradiation increases.

Fourier transform infrared (FT‐IR) was utilized to analyze the variation of chemical bonds and functional group during photocatalytic period (Figure S18, Supporting Information). There were intensive vibration peaks of ─OH group in the spectra of samples, one was the stretching vibration at 3450 cm^−1^ (O─H) and the other one was deformation vibration at 1630 cm^−1^ (H─O─H). The presence of these intense peaks signified the existence of a substantial quantity of water molecules adsorbed onto the surface of the sample. However, these two peaks exhibited a noticeable decrease undergoing photo‐illumination duration, with the deformation vibration peak of H─O─H nearly vanishing, thereby indicating a rapid reduction in hydroxyl groups present on the surface and active involvement in the ROS generation.

Furthermore, in order to deeply reveal the mechanism of surface functional group and chemical bonds variation process during light illumination period, the interaction between toluene and photocatalyst was traced through in situ DRIFTS spectra. Figure 4C illustrated that, in the dark state of adsorption, toluene typical absorption bonds displayed at 1476 cm^−1^. With the photocatalysis proceeding, the peak intensity of benzyl alcohol (1023 cm^−1^) progressively increased at the process of light on condition, implying that the gradual accumulation of benzaldehyde increase continuously (Figure 4D). The peaks of benzoate (1432 cm^−1^, 1480 cm^−1^), maleic anhydride (1500 cm^−1^) and benzaldehyde (1620 cm^−1^) experienced subsequent increase and deservedly reached equilibrium at a time point (Figure 4E), suggesting that following photocatalytic conversion of benzyl alcohol to benzaldehyde and benzoate occurred.^[^ ^45^ ^]^ In addition, it also showed some new bonds peaks occurrence and subsequent variation at 2980, 1720, 1240, 1100, 1040, and 725 cm^−1^ with light irradiation continuously proceeding, respectively (Figure S19, Supporting Information). These new peaks indicated that toluene or intermediates products (benzoquinone, malonic acid, glycolic acid, etc.) are adsorbed on the catalyst surface.^[^ ^46^ ^]^ Specifically, the bonds observed at 2980 cm^−1^ was assigned to the C─H vibration of the methyl group. And the bonds at 1720 cm^−1^ was assigned to C═O stretching. Its appearance due to aliphatic C═O groups indicated that aromatic ring cleavage occurred for the byproduct compounds. The bond at 1240 cm^−1^ was assignable to benzaldehyde and bonds at 1100 cm^−1^ can be assignable to the C─O─C stretching vibration mode. The sharp peak at 725 cm^−1^ was assigned to Ar‐H out‐plane deformation vibration, which proved the exist of benzene ring. Furthermore, based on the joint analysis of GC/FID, online‐MS and TDGC/MS (Figure S20, Supporting Information), the toluene decomposition intermediates detected could be further confirmed. It revealed that toluene conversion process could be conducted in more rapid process rate with visible‐light illumination duration postponing, especially for weakening ring‐opening activation energy as the rate‐determining reaction step of toluene conversion.^[^ ^47^ ^]^

As depicted in Figure 4F, the CdTe@TiO_2_ NTs presented an Electron Paramagnetic Resonance (EPR) response at g‐value of 2.003, provide corroborating proof concerning the existence of inherent oxygen vacancies possessing paramagnetic characteristics. Notably, the 5th CdTe@TiO_2_ NTs displayed the highest EPR signal intensity trend, ascribing to the formation of tight interfacial chemical interaction between Cd, Te SAs and TiO_2_ NTs. Subsequently, under light illumination, O‐related vacancies could establish trap states and hinder the recombination of photoinduced electron and hole pairs. Consequently, Cd/Te SAs coordination environment could serve as electrons acceptors, and therefore, likely tended to effectively lower the migration barrier of photo‐induced electrons. This further confirmed that the electronic interaction optimization regulating activation energy of toluene conversion was successful.

**Figure 5:**
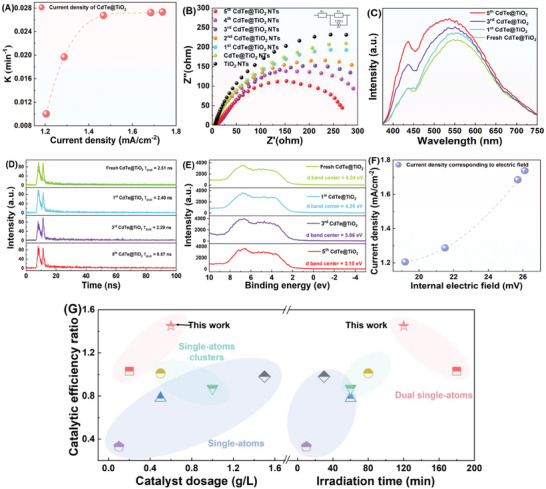
PEC measurements: A) Photocatalytic reaction rate constant versus current density, B) EIS at applied potential of 0.5 V and C) PL emission spectra D) TRPL spectroscopy under excitation wavelength of 325 nm light, E) Resolution XPS profiles in the valence band region F) current density versus internal electric field magnitude, G) Catalytic efficiency ratio between recycled and fresh catalysts for single‐atoms catalysts.

UV–vis Diffuse Reflectance Spectra (DRS) in Figure S21 (Supporting Information) highlighted that the optical absorption edge of the 5th CdTe@TiO_2_ NTs extended to 525 nm with strengthened capability for harvesting visible‐light, presenting the apparent red‐shift trend. Meanwhile, after five cycles of photocatalytic degradation, the photocurrent density in the CdTe@TiO_2_ NTs climbs apparently achieving the highest of 1.8 mA cm^−2^, expedited its superior efficiency in photo‐induced charge transfer when further in situ Cd/Te SAs anchored (Figure S22A, Supporting Information). And the photocatalytic reaction rate constant versus current density trend performed the increasing state (**Figure** **5**A). The 5th CdTe@TiO_2_ NTs electrode presented remarkably promoted photocatalytic activity and conversion efficiency with maximum IPCE value of roughly 7.5% compared with the fresh CdTe@TiO_2_ NTs (6.2%), shown in Figure S22B (Supporting Information). Furthermore, the Electrochemical Impedance Spectroscopy (EIS) Nyquist plots in Figure 5B presented electronic conductivity variation and remarkable decrease in electron transfer resistance for the CdTe@TiO_2_ NTs with various recycling times. The Tab. S1 presented fitting parameters of equivalent circuit for diverse photocatalysts, consistent with aforementioned results. The unique coordination environment of Cd─O─Te endowed a strong driving force to steer the electron transport from TiO_2_ NTs to atomic centers, providing ultrafast electron hopping,^[^ ^48^ ^]^ and subsequently, elevated the inactivation property. Mott−Schottky plots of the CdTe@TiO_2_ NTs exhibited more superior charge carriers density with light irradiation proceeding, in line with correspondingly notable electronic conductivity (Figure S23, Supporting Information).

Regarding fluorescence signal intensity, there is a remarkable reduction in the steady‐state Photoluminescence (PL) emission peak intensity for the 5th CdTe@TiO_2_ NTs compared to the CdTe@TiO_2_ NTs (Figure 5C). It originated from the suppressed inter‐band recombination of electrons‐holes pairs, facilitated by electrons traps established by interaction between Cd/Te dual SAs and supporting material TiO_2_ NTs. Moreover, the charge transfer kinetics enhancement induced by in situ uninterrupted Cd/Te SAs on bimetallic synergism mechanism was explored in terms of Time‐resolved Photoluminescence (TRPL) Spectroscopy (Figure 5D). The average lifetime of photo‐induced carriers, was estimated as 2.51, 2.40, 2.29, and 0.57 ns for the CdTe@TiO_2_ NTs, 1st CdTe@TiO_2_ NTs, 3^rd^ CdTe@TiO_2_ NTs and 5th CdTe@TiO_2_ NTs, respectively. It exhibited the most efficient electron transfer from TiO_2_ NTs to surface Cd/Te SAs through potential Cd─O─Te coordination channels to participate in the redox reductions, and thereby,^[^ ^49^ ^]^ more electrons could transfer to the Cd/Te dual sites as effective mediator for participating in the photocatalytic conversion.

In order to probing the evolution of the d‐band center values of creative Cd/Te dual SAs during continuous visible‐light illumination period for the CdTe@TiO_2_ NTs, the VB‐XPS spectra in the valence band region was estimated, displayed in Figure 5E. The photocatalytic process is typically hindered by excessive adsorption energy at the surface interface, impeding the desorption of the adsorbent. Conversely, insufficient adsorption energy fails to break the bonds in reactants to ensure swift progress of the catalytic conversion. The optimal adsorption energy level derived from the moderate d‐band center, therefore, guaranteeing both the expeditious advancement of the photocatalytic duration and the proficient desorption of the intermediates.^[^ ^50^ ^]^ And the upshift of the d‐band center with respect to the Fermi level followed the atomic dispersed Cd/Te sites quantity augment process, and thus, accompanied with the 5th CdTe@TiO_2_ NTs attaining an appropriate d‐band center value at 3.15 eV (Figure S24, Supporting Information). Additionally, the various CdTe@TiO_2_ NTs film electrode performed the smooth surface property shown in Figure S25 (Supporting Information). The enhancement of built‐in electric fields mediated by the incremental Cd/Te SAs decoration was further explored utilizing Kelvin probe force microscopy (KPFM), as evident in Figure S26 (Supporting Information). Notably, the surface potential of the 5th CdTe@TiO_2_ NTs reached a maximum of 26.0 mV compared with the internal electric field reached in the CdTe@TiO_2_ NTs (ΔE = 19.2 mV), 1st CdTe@TiO_2_ NTs (21.5 mV), and 3rd CdTe@TiO_2_ NTs (25.7 mV) (Figures S27 and S28, Supporting Information).^[^ ^49^ ^]^ Moreover, the gradually magnifying trend of surface potential profiles and corresponding built‐in electric field correlated with the photocatalysis. Given by this, the internal electric field magnitude variation among Cd/Te SAs and TiO_2_ NTs could regulate local charge distribution in the semiconductor.^[^ ^51^ ^]^ Notably, the interface properties between Cd/Te SAs and TiO_2_ NTs could also be monitored, and the corresponding surface potentials of the CdTe@TiO_2_ NTs with disparate light illumination duration revealed significant transformations at the interfaces. Hence, the contact potential difference found at the interface region substantiated the existence of a local electric field for transferring charges at the interface among Cd/Te SAs and TiO_2_ NTs. Besides that, with regard to the line scan across the interface, there are subtle surface potential differences between Cd, Te atoms and TiO_2_ NTs, derived from atomically dispersed Cd/Te SAs quantity variation. Hence, the incorporation of Cd/Te heteroatoms increase in the cavity would regulate the local charge distribution of the surrounding atoms, and thereby alter the local dipole moments.^[^ ^52^, ^53^ ^]^ Namely, the photocurrent density was controlled through internal electric field magnitude (Figure 5F). Thus, the catalytic efficiency of the CdTe@TiO_2_ NTs performed the most excellent durability among single‐atoms catalysts research, as shown in Figure 5G.^[^ ^52^, ^53^, ^54^, ^55^, ^56^, ^57^ ^]^

**Figure 6:**
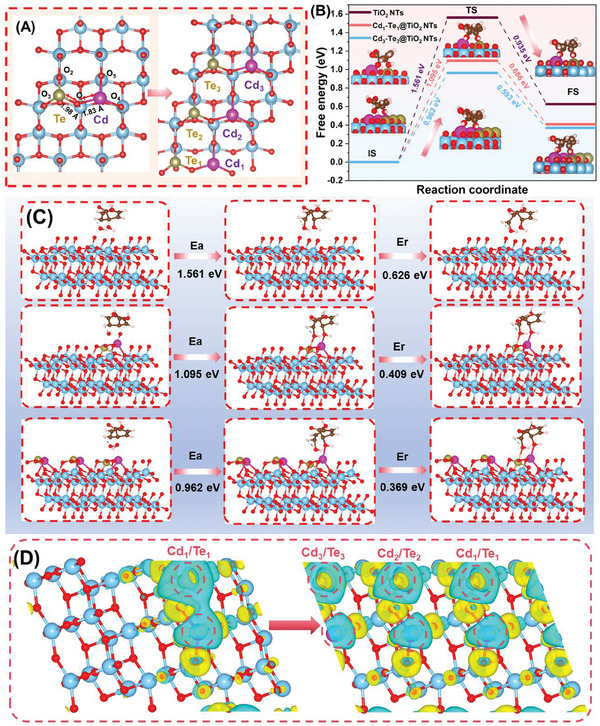
A) Calculated bond length variation of Cd─O and Te─O on Cd_1_Te_1_@TiO_2_ NTs and Cd_3_Te_3_@TiO_2_ NTs; B) Gibbs free energy diagrams and the pathways for photo‐oxidation ring‐opening process for benzoic acid and C) Calculated reaction energy barriers of ring‐opening processes on TiO_2_ NTs, Cd_1_Te_1_@TiO_2_ NTs, and Cd_3_Te_3_@TiO_2_ NTs; D) Charge density distribution of Cd_1_Te_1_@TiO_2_ NTs and Cd_3_Te_3_@TiO_2_ NTs with 0.002 e Å^−3^. (Blue ball represents Ti atom; Red ball represents O atom; Purple ball represents Cd atom; Brown ball represents Te atom; Yellow and cyan isosurfaces denote electron accumulation and depletion regions, respectively).

To further realistically simulate atomically dispersed Cd/Te quantities variation in the CdTe@TiO_2_ NTs experiencing multiple rounds of photoreduction process, one Cd/Te atoms were adopted in TiO_2_ NTs to represent the fresh CdTe@TiO_2_ NTs and three Cd/Te atoms were launched in TiO_2_ NTs to emulate the recycled CdTe@TiO_2_ NTs in DFT modelling, expressed as Cd_1_Te_1_@TiO_2_ NTs and Cd_3_Te_3_@TiO_2_ NTs, respectively. The optimized structure model of TiO_2_ NTs, Cd_1_Te_1_@TiO_2_ NTs, and Cd_3_Te_3_@TiO_2_ NTs were visually presented in side view (Figure S29, Supporting Information) and top view (Figure S30, Supporting Information). The optimized model revealed the coordination environment of Cd and Te species, and thus effectively differentiates between the Cd─O and Te─O configurations in the first scattering shell originating from the regulation and stabilization of metals SAs by anchoring O sites. The absence of Cd─Cd and Te─Te configurations in the simulated structure clearly ruled out any possibility of metal aggregation. The bond lengths of Cd─O (1.85 Å) and Te─O (2.01 Å) in Cd_1_Te_1_@TiO_2_ NTs were obtained, accompanied with slightly decreasing of Cd_1_‐O (1.83 Å) and Te_1_‐O (1.98 Å) in Cd_3_Te_3_@TiO_2_ NTs (**Figure** **6**A). It derived updated electronic states and property surrounding Cd─Te dual‐metal coordination sites, contributed the promoting photocatalysis.^[^ ^54^ ^]^

To unveil the underlying photocatalytic activity enhancement, the DFT calculation was employed to investigate the Gibbs free energy for identifying the transition state and energy barrier, as well as examined photocatalytic kinetics with persistent generation of atomically dispersed Cd/Te sites. And the optimized structure for intermediates initial state (IS), transition state (TS), and final state (FS) were presented for TiO_2_ (Figure S31, Supporting Information), Cd_1_Te_1_@TiO_2_ (Figure S32, Supporting Information), and Cd_3_Te_3_@TiO_2_ (Figure S33, Supporting Information). For photocatalytic toluene conversion, the ring‐opening process is the rate‐determination step. Theoretically, benzoic acid species was the crucial intermediate in toluene degradation into CO_2_ since benzene ring‐opening procedure oxidized by ·OH would be attained at benzoic acid intermediate on the CdTe@TiO_2_ NTs. And the pivotal rate‐determination reaction involved the elementary step as follows:

(1) $$C_{6}H_{5}\mathrm{COO}H^{*}+2\cdot \mathrm{OH}\to \mathrm{HOOCOH}C_{6}H_{5}OH^{*}+e^{-}$$

Therefore, progressively accelerated photocatalysis accompanied with Cd/Te SAs increasing could be essentially explained through calculated Gibbs free energy variation of ring‐opening of benzoic acid intermediate. As displayed in Figure 6B, the energy profiles highlighted that the potential conversion pathway was from C_6_H_5_COOH* initial state to HOC_6_H_4_OHCOOH* transition state and further to HOOCOHC_6_H_5_OH* final state in terms of the estimated energy barriers. Apparently, we demonstrated that the energy barriers of C_6_H_5_COOH* ring‐opening on Cd_3_Te_3_@TiO_2_ NTs was 0.96 eV, which was lower than that of Cd_1_Te_1_@TiO_2_ NTs (1.10 eV) and TiO_2_ NTs (1.56 eV) (Figure 6C). In addition, the formation free energy of HOOCOHC_6_H_5_OH* final state on Cd_3_Te_3_@TiO_2_ NTs (0.36 eV) also featured lower tendency than that of Cd_1_Te_1_@TiO_2_ NTs (0.41 eV) and TiO_2_ NTs (0.63 eV). Given by this, it would be more beneficial to effectually stabilize the HOOCOHC_6_H_5_OH* intermediate by the charge‐enriched Cd/Te sites with Cd/Te SAs increasing, which thus explained the enhanced photocatalysis of Cd_3_Te_3_@TiO_2_ NTs compared to Cd_1_Te_1_@TiO_2_ NTs and TiO_2_ NTs. Moreover, the reduced thermodynamic energy barrier suggested that the incorporation of in situ growing atomic Cd/Te species as active centers boosted dynamic electron transfer from the TiO_2_ NTs support to atomically dispersed Cd/Te sites.^[^ ^54^ ^]^

The charge density difference diagram was executed to scrutinize the charge distributions across the metal‐support interface, and consequently, provided evidences of the distinct charge distribution in the CdTe@TiO_2_ NTs, with the noticeable electronic accumulation area around the Te sites (yellow isosurfaces) and electron depletion near Cd sites (cyan isosurfaces) (Figure 6D). And the charge density distributions of Cd_1_Te_1_@TiO_2_ NTs in top view (Figure S34, Supporting Information) and side view (Figure S35, Supporting Information), as well as the Cd_3_Te_3_@TiO_2_ NTs in top view (Figure S36, Supporting Information) and side view (Figure S37, Supporting Information) were shown. Thus, Cd SAs could serve as the particular electronic mediator for facilitating the electrons migration toward active Te SAs sites, enabling high toluene activation efficiency and intermediates stability. The increasing of Cd/Te SAs sites resulted in augmentation of charge density around Te SAs, effectively inducing electrons redistribution around the Cd/Te SAs, consistent with the KPFM analysis. Thus, it validated Schottky effect causing a swift electron‐hole pairs separation, as well as more prominent electron acquisition property with Cd/Te SAs sites growing. Consequently, the charge density variation conveyed an exquisite atomic‐level collaboration across the functional Cd/Te dual‐metal sites on the CdTe@TiO_2_ NTs experiencing persistent growing of SAs sites.

Specifically, based on the Bader charge distribution analysis (Figure S38A, Supporting Information), it highlighted that the Cd‐atoms (1.29 e) and Te‐atoms (−2.16 e) in Cd_1_Te_1_@TiO_2_ NTs were prone to undergo electron donating and acquisition due to correspondingly negative and positive Bader charge distribution, respectively, in accordance with electric density difference. Clearly, this conveyed that Cd SAs sites could perform as the electron donor, while Te SAs sites took the role of the electron acceptor. In comparison with the Cd_1_Te_1_@TiO_2_ NTs, the charge quantity in the individual Cd and Te atom in Cd_3_Te_3_@TiO_2_ NTs exhibited no significant variation (Figure S38B, Supporting Information), and thereby, illustrating that growing Cd/Te SAs could produce more intensitive driving force for charge migration from TiO_2_ NTs support to monolithic bimetallic Cd/Te sites.

The charge density disparity within the CdTe@TiO_2_ NTs gave rise to a resulting net charge accumulation, ultimately leading to the formation of a local built‐in electric field.^[^ ^57^ ^]^ DFT calculations theoretically predicted that the internal electric field directed toward TiO_2_ NTs. The polarity of the built‐in electric field experienced a gradual promotion with SAs increasing, accompanied by the depletion of interface charge. Consequently, it demonstrated the more intense electronic states interactions of increasing Cd‐Te dual SAs sites in facilitating energy barriers weakening, originating from rearranging electronic structure and modulating electron density surrounding Cd─O─Te dual‐metal coordination sites.^[^ ^56^ ^]^

**Figure 7:**
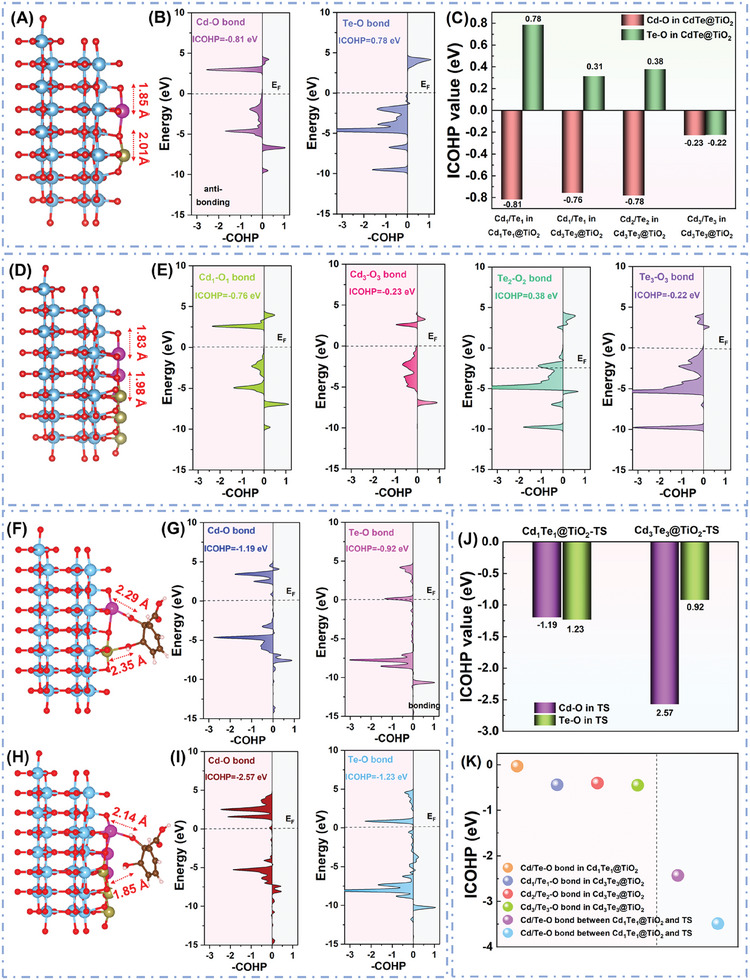
A,B) Projected crystal orbital Hamilton population (COHP) for the Cd─O/Te─O bond in Cd1Te1@TiO2 NTs, C) ICOHP value for the Cd─O/Te─O bond in Cd1Te1@TiO2 and Cd3Te3@TiO2 NTs, D,E) COHP for the Cd─O/Te─O bond in Cd3Te3@TiO2 NTs; F,G) COHP for the Cd─O/Te─O bond between Cd1Te1@TiO2 and HOC6H4OHCOOH* TS (H,I) COHP for the Cd─O/Te─O bond between Cd3Te3@TiO2 and HOC6H4OHCOOH* TS, J) ICOHP value for Cd─O/Te─O bond between Cd1Te1@TiO2/Cd3Te3@TiO2 and HOC6H4OHCOOH* TS, K) ICOHP value for metal‐O bond. Bonding and antibonding states are shown on the right and left regions, respectively. The horizontal gray lines indicate the Fermi level.

To acquire the deeper insight into the bond strength of Cd─O/Te─O formed in the CdTe@TiO2 NTs, the corresponding COHPs in **Figure** **7** presented the bonding orbitals and occupied anti‐bonding orbitals on the right (light grey area) and left (light purple area) sides, respectively. Gaining advantages from the elevated occupied d orbitals of Cd (Figure 7A,B), the Cd─O bond exhibited the more pronounced anti‐bonding orbital intensity above the Fermi level compared to that of the Te─O bond for both Cd1Te1@TiO2 (Figure 7C) and Cd3Te3@TiO2 NTs (Figure 7D,E; Figure S39, Supporting Information). As a consequence, more intense Cd─O bond displayed that more intermediates adsorption was favored on the Cd SAs sites. Remarkably, the filling of the antibonding orbital population below the Fermi level (EF) decreases when Cd/Te SAs growing, originating from the downshift of the O 2p band and the upshift of the Cd/Te 3d band. It suggested a promotion in the bonding strength of metal‐O bonds and more intensitive interactions occurred between Cd/Te and O atoms with SAs increasing. Additionally, the integrated COHP value (ICOHP) up to the Fermi level for the Cd1‐O1/Te1‐O1 bond as a whole on the Cd1Te1@TiO2 NTs was striking at −0.03 eV, which was slightly negative than that of the Cd1‐O1/Te1‐O1 bond (−0.44 eV), Cd1‐O1/Te1‐O1 bond (−0.40 eV), and Cd1‐O1/Te1‐O1 bond (−0.45 eV) in Cd3Te3@TiO2 NTs. This observation unequivocally corroborated that the stability of the Cd─O, Te─O bonds, and subsequent metal‐O bonds interactions intensity were significantly optimized with Cd/Te sites expanding.

Based on the combination of Lowest Energy principle with the aforementioned analysis of ICOHP of metal‐O, it is revealed that transition state of HOC6H4OHCOOH* presented a preferential adsorption to the in‐plane Cd sites in both Cd1Te1@TiO2 and Cd3Te3@TiO2 NTs, and subsequently intelligently transitions toward the interface of Cd/Te hetero‐phase due to the stronger force exerted by the Cd─O/Te─O bonds at the Cd/Te interface. The interaction between HOC6H4OHCOOH* TS and Cd1/Te1 in Cd1Te1@TiO2 NTs (Figure 7F,G), in contrast, was relatively weaker compared to that observed bond strength between HOC6H4OHCOOH* TS and Cd1/Te1 in Cd3Te3@TiO2. This resulted in the preferential adsorption and binding capacity of HOC6H4OHCOOH* TS from the in‐plane Cd/Te phase sites of Cd3Te3@TiO2 with SAs increasing during the photo‐conversion process, as supported by the aforementioned COHP calculations. Therefore, it speculated that the electron cloud between HOC6H4OHCOOH* TS and the Cd3Te3@TiO2 surface exhibited a greater extent of expansion compared to that observed on the surfaces of Cd1Te1@TiO2, indicating a facile transfer of electrons from partially occupied d orbitals at Cd/Te sites to empty p orbitals of TS of HOC6H4OHCOOH* intermediates (Figure 7H,I). The observations were in excellent agreement with the obtained free energy barriers of HOC6H4OHCOOH* TS formation on the catalytic model surface. The resulting strengthening Cd─O/Te─O bonding between the CdTe@TiO2 and HOC6H4OHCOOH* TS was furthered verified by the ICOHP (Figure 7J), which displayed promoted average Cd─O/Te─O interactions ranging from −2.43 to −3.49 eV (Figure 7K). Consequently, HOC6H4OHCOOH* TS was remarkably strengthened on Cd3Te3@TiO2, emphasizing the crucial role of increasing Cd/Te SAs sites in stabilizing the key intermediates in aspects of optimizing the electrons interactions between Cd/Te and HOC6H4OHCOOH* TS.

## 3 Conclusion

In summary, we have successfully synthesized a dual‐metal‐doped CdTe@TiO2 NTs by precisely controlling the specifically atomic coordination environment of Cd─Te through a self‐seeded process in photoreduction phase. The oxygen‐mediated dimer catalytic mechanism, as revealed by theoretical calculation study, unveils a novel mode of interaction between the oxygen‐coordinated Cd and Te SAs, characterized by nonbonding yet highly interactive behavior. Experimental characterizations and DFT calculations reveal the distinct roles in progressively enhancing photocatalytic performance played by Cd and Te atomic sites increasing, as well as their synergistic effects in photocatalysis by leveraging their respective advantages. Specifically, photoelectric dynamic characterizations demonstrate that the growing bimetallic Cd─Te species facilitates charge carrier dynamics, while TR‐PL spectra, KPFM analysis confirm that persistent generation of atomically dispersed Cd─Te sites promotes electron transfer to enable long‐lasting charge separation. Furthermore, in situ DRIFTS measurements, Barder charge, Gibbs free energy, and COHP calculation suggest that the synergistic effect on electronic density regulation of Cd─Te SAs sites would account for weakening activation energy of decisive ring‐opening. The study offers novel insights into the photocatalysts with atomic precision and cooperation via regulation of regulation of the neighboring coordination environment, thereby inspiring a rational approach to designing highly‐durable dual‐metal SAs catalysts for efficient solar energy conversion.

## Conflict of Interest

The authors declare no conflict of interest.

## Supporting information



Supporting Information

## Data Availability

The data that support the findings of this study are available from the corresponding author upon reasonable request.
