# Supplementary material for: Revealing the Principle of Progressively Enhanced Photocatalytic Reactivity in Dual Single‐Atoms‐Mediated Electronic Interactions Optimization of Cd/Te‐TiO2
Source: Adv Sci (Weinh). 2025 Mar 17;12(18):2413379. doi: 10.1002/advs.202413379 (PMC12079335; doi:10.1002/advs.202413379)
Supplement: Supplementary file 1 — Supporting Information [file ADVS-12-2413379-s001.docx]

**Supporting Information**

**Revealing the Principle of Progressively Enhanced Photocatalytic Reactivity in Dual Single-Atoms Mediated Electronic Interactions Optimization of Cd/Te-TiO_2_**

Yihang Zhang, Hao Zhao, Shan Jiang, Yanrong Zhang, Yong Chen, Jianyu Gong^*^

Hubei Key Laboratory of Multi-media Pollution Cooperative Control in Yangtze Basin,

School of Environmental Science & Engineering,

Huazhong University of Science and Technology (HUST),

1037 Luoyu Road, Wuhan, Hubei, 430074, China

^*^ Corresponding author contact information:

Professor Jianyu Gong, Ph.D.

E-mail address: [jygong@hust.edu.cn](mailto:jygong@hust.edu.cn)

**List of** [**Supporting Information**](#_Toc5258)

[Figure S1. SEM images of TiO_2_ NTs (the inset is the cross of TiO_2_ NTs) 9](#_Toc20255)

[Figure S2. SEM images of CdTe@TiO_2_ NTs (A) in 1um and (B) in 200 nm scale 10](#_Toc28028)

[Figure S3. FSEM images of cross of CdTe@TiO_2_ NTs 11](#_Toc27034)

[Figure S4. XRD pattern of as-synthesized TiO_2_ NTs and CdTe@TiO_2_ NTs 12](#_Toc2958)

[Figure S5. HRTEM images observation (A) in 5 nm and (B) in 10 nm for CdTe@TiO_2_ NTs 13](#_Toc20218)

[Figure S6. (A) HAADF-STEM, (B) Energy dispersive X-ray spectra of fresh CdTe@TiO_2_ NTs (C) EDX spectra of individual Cd and Te elements (single atomic Cd/Te sites high-lighted by red circles; Cd/Te clusters high-lighted by yellow circles) 14](#_Toc1102)

[Figure S7. The SEM for (A) 1^st^ CdTe@TiO_2_ NTs, (B) 2^nd^ CdTe@TiO_2_ NTs, (C) 3^rd^ CdTe@TiO_2_ NTs, (D) 4^th^ CdTe@TiO_2_ NTs and (E) and 5^th^ CdTe@TiO_2_ NTs 15](#_Toc13114)

[Figure S8. The corresponding EDS spectrum for (A) CdTe@TiO_2_ NTs, (B) 1^st^ CdTe@TiO_2_ NTs, (C) 3^rd^ CdTe@TiO_2_ NTs, (D) 5^th^ CdTe@TiO_2_ NTs 16](#_Toc4267)

[Figure S9. HAADF-STEM with energy dispersive X-ray spectra of (A) 5^th^ CdTe@TiO_2_ NTs; (B) Ti, O, Cd, Te elements in 5^th^ CdTe@TiO_2_ NTs 17](#_Toc29929)

[Figure S10. HAADF-STEM images of 5^th^ CdTe@TiO_2_ NTs (A) in 1nm (B-D) in 2nm (E) in 5 nm (single atomic Cd sites high-lighted by red circles; Cd clusters high-lighted by yellow circles) 18](#_Toc24812)

[Figure S11. HAADF-STEM with energy dispersive X-ray spectra of (A) 5^th^ CdTe@TiO_2_ NTs; (B) Ti, O, Cd, Te elements in 5^th^ CdTe@TiO_2_ NTs 19](#_Toc26460)

[Figure S12. Recycling test for toluene degradation curve on TiO_2_ NTs 20](#_Toc30355)

[Figure S13. Recycling test for toluene degradation curve on (A) CdTe@TiO_2_ NTs (HNO_3_ served as pH value regulators) (B) TiO_2_ NTs (H_2_SO_4_ served as pH value regulators without Cd/Te during electrochemical deposition process), (C) CdTe@TiO_2_ NTs (pH value in electrolyte did not be regulated) 21](#_Toc7044)

[Figure S14. Recycling test for toluene degradation curve on (A) Cd@TiO_2_ NTs and (B) Te@TiO_2_ NTs 22](#_Toc18018)

[Figure S15. XPS peak position spectra of photocatalysis: (A) Ti 2p, (B) O 1s, (C) Cd 3d, (D) Te 3d of as-synthesized TiO_2_ NTs, CdTe@TiO_2_ NTs and recycled CdTe@TiO_2_ NTs with various repeating times 23](#_Toc1130)

[Figure S16. Atomic structure and coordination state investigation. (A) WT-EXAFS contour plots at the Cd K-edge of recycled CdTe@TiO_2_ NTs and reference; (B) WT-EXAFS contour plots at the Te K-edge of recycled CdTe@TiO_2_ NTs and reference 24](#_Toc11097)

[Figure S17. Raman spectra of as-synthesized TiO_2_ NTs, CdTe@TiO_2_ NTs and recycled CdTe@TiO_2_ NTs with various repeating times at the Raman shift between (A) 100-200 cm^-1^ and (B) 240-320 cm^-1^. 25](#_Toc31908)

[Figure S18. FT-IR measurement of gaseous toluene in PC reaction on TiO_2_ NTs, CdTe@TiO_2_ NTs and recycled CdTe@TiO_2_ NTs under visible light illumination (λ=420nm) 26](#_Toc15088)

[Figure S19. In-situ DRIFTS spectrum on CdTe@TiO_2_ NTs under visible light illumination (λ=420 nm) at gaseous toluene atmosphere at 2800-3100 cm^-1^ 27](#_Toc7221)

[Figure S20. TD-GC/MS results of removal of toluene on (A) CdTe@TiO_2_ NTs and (B) 5^th^ CdTe@TiO_2_ NTs 28](#_Toc19419)

[Figure S21. UV-vis absorption spectra for as-synthesized TiO_2_ NTs, CdTe@TiO_2_ NTs and recycled CdTe@TiO_2_ NTs with various repeating times 29](#_Toc2748)

[Figure S22. Transient photocurrent density for as-synthesized TiO_2_ NTs, CdTe@TiO_2_ NTs and recycled CdTe@TiO_2_ NTs 30](#_Toc19470)

[Figure S23. Mott−Schottky plots for as-synthesized fresh CdTe@TiO_2_ NTs and 5^th^ CdTe@TiO_2_ NTs 31](#_Toc6118)

[Figure S24. The d-band center absolute value of the catalysts calculated from high-resolution valence band XPS 32](#_Toc10217)

[Figure S25. The AFM of topography on of (A) CdTe@TiO_2_ NTs, (B) 1^st^ CdTe@TiO_2_ NTs, (C) 3^rd^ CdTe@TiO_2_ NTs (D) and 5^th^ CdTe@TiO_2_ NTs film electrode 33](#_Toc29805)

[Figure S26. Extractive surface potentials for as-synthesized TiO_2_ NTs, CdTe@TiO_2_ NTs and recycled CdTe@TiO_2_ NTs 34](#_Toc21421)

[Figure S27. The surface potentials of (A) CdTe@TiO_2_ NTs, (B) 1^st^ CdTe@TiO_2_ NTs, (C) 3^rd^ CdTe@TiO_2_ NTs (D) and 5^th^ CdTe@TiO_2_ NTs film electrode measured with KPFM 35](#_Toc30775)

[Figure S28. The estimated surface potential data extracted from the potential distribution: (A) CdTe@TiO_2_ NTs, (B) 1^st^ CdTe@TiO_2_ NTs, (C) 3^rd^ CdTe@TiO_2_ NTs and (D)5^th^ CdTe@TiO_2_ NTs measured with KPFM technology 36](#_Toc20905)

[Figure S29. The optimized structure model in side view of (A) TiO_2_ NTs, (B) Cd_1_Te_1_@TiO_2_ NTs and (C) Cd_3_Te_3_@TiO_2_ NTs (Blue ball represents Ti atom; Red ball represents O atom; Purple ball represents Cd atom; Brown ball represents Te atom) (Cd_1_Te_1_@TiO_2_ NTs indicates that one Cd and Te atom is introduced in CdTe@TiO_2_ NTs; Cd_3_Te_3_@TiO_2_ NTs indicates that three Cd and Te atom are introduced in CdTe@TiO_2_ NTs) 37](#_Toc1620)

[Figure S30. The optimized structure model in top view of (A) TiO_2_ NTs, (B) Cd_1_Te_1_@TiO_2_ NTs and (C) Cd_3_Te_3_@TiO_2_ NTs 38](#_Toc8896)

[Figure S31. The optimized structure model of benzoic acid adsorbed in TiO_2_ NTs in side view for (A) IS, (B) TS and (C) FS; The optimized structure model of benzoic acid adsorbed in TiO_2_ NTs in top view for (D) IS, (E) TS and (F) FS (Blue ball represents Ti atom; Red ball represents O atom; Black ball represents C atom; White ball represents O atom) 39](#_Toc26083)

[Figure S32. The optimized structure model of benzoic acid adsorbed in Cd_1_Te_1_@TiO_2_ NTs in side view for (A) IS, (B) TS and (C) FS; The optimized structure model of benzoic acid adsorbed in TiO_2_ NTs in top view for (D) IS, (E) TS and (F) FS 40](#_Toc15417)

[Figure S33. The optimized structure model of benzoic acid adsorbed in Cd_3_Te_3_@TiO_2_ NTs in side view for (A) IS, (B) TS and (C) FS; The optimized structure model of benzoic acid adsorbed in TiO_2_ NTs in top view for (D) IS, (E) TS and (F) FS 41](#_Toc24359)

[Figure S34. Charge density distribution of Cd_1_Te_1_@TiO_2_ NTs in top view with 0.002 e Å^-3^ (Yellow and cyan isosurfaces denote electron accumulation and depletion regions, respectively) 42](#_Toc13563)

[Figure S35. Charge density distribution of Cd_1_Te_1_@TiO_2_ NTs in side view with 0.002 e Å^-3^ 43](#_Toc9818)

[Figure S36. Charge density distribution of Cd_3_Te_3_@TiO_2_ NTs in top view with 0.002 e Å^-3^ 44](#_Toc11671)

[Figure S37. Charge density distribution of Cd_3_Te_3_@TiO_2_ NTs in side view with 0.002 e Å^-3^ 45](#_Toc10752)

[Figure S38. Bader charge distribution analysis on (A) Cd_1_Te_1_@TiO_2_ NTs and (B) Cd_3_Te_3_@TiO_2_ NTs 46](#_Toc20954)

[Figure S39. Projected crystal orbital Hamilton population (COHP) for (A) the Cd_2_-O_2_ and (B) Te_2_-O_2_ bond in Cd_3_Te_3_@TiO_2_ NTs 47](#_Toc18618)

[Figure S40. Photocatalytic performance test device 48](#_Toc1982)

[Table S1. Charge transfer resistances 49](#_Toc21240)

**Methods**

**Reagents** Sodium sulfate (Na_2_SO_4_), hydrofluoric acid (HF), nitrate acid (HNO_3_), sulfuric acid (H_2_SO_4_), anhydrous ethanol, acetone, methanol, sodium fluoride (NaF), antimony oxides (TeO_2_), cadmium sulfate (CdSO_4_) and cadmium chloride (CdCl_2_) were purchased from Sinopharm Chemical Regent Co., Ltd. (Shanghai, China). All reagents were of analytical grade. Ti foil (0.5 mm thick, 99% purity) was obtained from Baoji Titanium Industry Co., Ltd., China. All experiments were prepared with deionized water.

**Preparation of TiO_2_ NTs** The Ti foil (1 × 1.5 cm) used in the preparation of TiO_2_ NTs were first cleaned in DI water and ethanol, followed by chemical polishing with a solution consisting of HF: HNO_3_: H_2_O = 1:3:6 (volume ratio), and rinsed with DI water and dried by nitrogen gas. Then, the cleaned Ti foil was vertical placed in a two-electrode electrochemical system with a Pt foil as cathode supplied a constant potential (60 V) by using a direct current power equipment. The electrolyte was prepared by mixing deionized water and glycerin with a volume ratio of 1:9 containing 0.27 M NH_4_F. Then, the as-formed films were sintered at desired temperature for 2 h to obtain TiO_2_ NTs.

**Preparation of CdTe****@TiO_2_ NTs** CdTe*@*TiO_2_ NTs were fabricated via electrochemical deposition in a three-electrode cell. The obtained TiO_2_ NTs, Pt foil and saturated calomel electrode (SCE) worked as working electrode, counter electrode and reference electrode, respectively. To fabricate CdTe*@*TiO_2_ NTs, the TiO_2_ NTs were immersed in 50 mL electrolyte which was composite of CdSO_4_ (0.1M), TeO_2_ (0.1mM), H_2_SO_4_ and water by supplying -0.4 V (vs. SCE) potential under water bath condition (80 ^o^C) lasting for desired time. Then, Cd/Te was formed on the TiO_2_ NTs according to equation as follows:

$\mathbf{3} \mathbf{H}^{\boldsymbol{+}}\mathbf{+}\mathbf{Cd}^{\boldsymbol{2}\boldsymbol{+}}\mathbf{+}\mathbf{HTeO}_{\boldsymbol{2}}^{\boldsymbol{+}}\mathbf{+}\mathbf{6}\mathbf{e}^{\boldsymbol{-}}\boldsymbol{\to}\mathbf{CdT}\mathbf{e}\mathbf{+}\mathbf{2}\mathbf{H}_{\boldsymbol{2}}\mathbf{O}$ (2)

Then, the as-prepared CdTe*@*TiO_2_ NTs were further washed with water and immersed in CdCl_2_ (0.1 mM)/methanol solution for 2 min, then followed treated at 300 ℃ for 1 h to obtain final films.

**Photocatalytic degradation of gaseous toluene** The photocatalytic degradation of VOC was conducted in a special sealed reactor made by ourselves equipped with a cooling system. The concentration of gaseous toluene in the reactor was about 100 ppm. The lamp was placed on top of the quartz reactor. And, the fabricated film was placed at the bottom of reactor facing the lamp. The light source was a 300 W arc-xenon lamp (Abet Technologies, Inc., Milford, CT). Photocatalytic performance test device was shown in Figure S40. The power density was controlled by adjusting the distance between the lamp and the reactor. And the incident light intensity was measured with thermopile head (FA, Beijing Normal University) connected to an optical power meter and it was determined to be about 5 mW/cm^2^. All PC experiments were conducted at room temperature.

**Material Characterization** The crystal structure of the samples was examined by X-ray spectroscopy (XRD, 2θ ranges from 10° to 80°, using a diffractometer at 40 kV and 40 mA). The morphology and element distribution were characterized by transmission electron microscopy (TEM, JEM-2100F electron microscope operating at 200 kV) and HAADF-STEM imaging (FEI Tecnai G2 F30 microscope equipped with an EDAX energy-dispersive X-ray analysis system). The chemical compositions were analyzed by X-ray photoelectron spectroscopy (XPS, conducted on a 5300 ESCA instrument (Perkin-Elmer PHI Co., USA) using an Al Kα X-ray source at a power of 250 W). The UV-Vis diffuse reflection spectra (UV-Vis DRS) was obtained by the Scan UV-Vis spectrophotometer (UV-2600, Shimadzu) with 100% BaSO_4_ as a reference. The photoluminescence (PL) spectra were acquired using an Edinburgh Analytical Instrument (FLS1000) at room temperature with a xenon lamp as the excitation source at the excitation wavelength of 325 nm of as-prepared catalyst. Time-resolved PL spectra were determined on a HORIBA Delta Pro under 325 nm excitation. The pump laser of 325 nm light was generated by a Q-switched Nd: YAG laser system using a third harmonic process on its fundamental output. The signal was collected by a monochromator and then recorded by a sensitive photomultiplier and traced by a TDS 3012C digital signal analyzer. Time-resolved PL experiment was carried out in air-saturated aqueous solutions. At the sample position, the average power was around 80 mW for the pump beam. Time resolved fluorescence decay traces were deconvoluted from the signal and fitted using the FluoFit 4.4 package (Picoquant GmbH). The experimental decay traces were fitted to multi-exponential functions via a Levenberg-Marquardt algorithm-based nonlinear least-squares error minimization deconvolution method. Usually, up to four different exponential terms were used to fit the experimental decay traces. The quality of the fits was judged by the reduced chi-squared method, the weighted residuals and the correlation functions. The latter two were checked for random distributions. Subsequently, the average photoluminescence lifetime was calculated by the following equation:


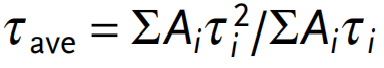
  (1)

Where A_i_ are the pre-exponential factors and τ_i_ are the lifetimes obtained in the multiexponential fitting of the decay kinetics.

The d-band centers were estimated utilizing X-ray photoelectron spectroscopy (XPS, JP- 9010 MC, JEOL) equipped with the Mg Kα X-ray source at a bias voltage of −5V. The XPS spectra of CdTe@TiO_2_ NTs in the valence band region were measured. And the d-band center values were determined through integrating the projected d-band density of state (DOS) up to the Fermi level, shown as the following formula:

$\text{d-band center = }\frac{\int_{\text{-}\infty}^{\text{Ef}} \text{R(E)EdE}}{\int_{\text{-}\infty}^{\text{Ef}} \text{R(E)dE}}$ (2)

Where R(E) was the XPS intensity after background subtraction, E was the binding energy, E_f_ was the Fermi energy level, and the calibration of the XPS revealed that the E_f_ of all samples was approximately 0. The background-corrected spectra of catalysts were obtained by subtracting the background spectra (obtained for the TiO_2_) from the XPS spectra of catalysts, and was used to calculate the values of d-band centers of Cd/Te deposited on TiO_2_ NTs.

The apparent quantum efficiency (AQE) was calculated according to the equations mentioned below:

$$AQE=\frac{N_{E}}{N}\times100\%$$

N_E_ is the total number of electrons transferred during the reaction.

N_E_ = K × the number of generated CO_2_ molecules × N_A_

K is the number of electrons transferred per mole of CO_2_ produced for toluene. For toluene:

$$\begin{matrix} N_{E}=\text{ }K\times thenumberofgeneratedCO_{2}molecules\text{ }\times\text{ }N_{A} \\ \text{ }=\text{ }\frac{(36/7)\times723.59\times25\times10^{-9}\times6.02\times10^{23}}{44}\text{ }=\text{ }1.27\times10^{18} \end{matrix}$$

$$AQE=\frac{N_{E}}{N}\times100\%=\frac{1.27\times10^{18}}{6.37\times10^{19}}\times100\%=1.99\%$$

The surface potential was monitored by KPFM in dark using AFM system (SPA-300HV) equipped with a SKPM module. X-ray absorption spectroscopy (XAS) studies were conducted at the BL08B2* of SPring-8 (8 GeV, 100 mA) in Japan. The X-ray beam was mono-chromatized using a water-cooled Si (111) double-crystal monochromator and focused with Rh-coated focusing mirrors with the beam size of 2.0 mm in the horizontal direction and 0.5 mm in the vertical direction around sample position, to obtain XAS spectra both in near and extended edge. Cd and Te foil samples were used as references. EXAFS fitting was applied through Athena and Artemis software. Wavelet transformation (WT) was employed using the software package developed by Funke and Chukalina using Morlet wavelet with k = 12, σ = 1. In-situ Fourier transform infrared (FT-IR) spectroscopy was conducted using the diffuse reflectance mode of a Bruker INVENIO S FT-IR spectrometer from Germany. The spectral scanning range was 4000-800 cm^-1^ with a resolution of 4 cm^-1^.Electron spin resonance (ESR) spectra were obtained with a Bruker EPR A300 spectrometer at room temperature under visible light (λ>420nm). The detection method of DMPO-·OH and DMPO-·O_2_^-^ was as follows: 5 mg sample was dissolved in 5 mL solvent, 56 μL DMPO was mixed with the solvent, the solvent for detecting OH was water, and the solvent for detecting ·O_2_^-^ was methanol. Singlet oxygen (^1^O_2_) was measured by dispersing 5 mg samples in 150 mmol/L TEMP solution prepared with water as solvent, and then taking the mixture for 10 min and 15 min respectively under visible light irradiation for in-situ test. The excitation wavelength (WL) was set as 344 nm, the emission starting WL was 440 nm, the emission end WL was 480 nm and the slit width was 2.5 nm.

**Photoelectrochemical (PEC) Characterization** The PEC characterizations were conducted by an electrochemical workstation (CHI660E) using a conventional three electrode system, in which conductive glass coated with photocatalytic materials was used as the working electrode, Pt electrode was used as the counter electrode, saturated Ag/AgCl electrode was used as the reference electrode, and 0.1 mol/L Na_2_SO_4_ solution was used as the electrolyte solution. The frequency range and AC amplitude of EIS were set as 0.01 to 1×10^5^ Hz and 0.5 V, respectively. Mott-Schottky plots were examined with a scan rate of 5 mV/s at 1000 Hz. Fabrication procedures of preparing working electrode as follows: 2 mg of photocatalyst powders were dispersed in mixed solution containing 800 µL of deionized water, 150 µL of ethanol and 50 µL of Nafion with ultrasound treatment for 30 min. Then the mixed solution was dropped and dried several times on FTO substrate (1×1 cm^2^). In addition, the light source of photocurrent response was supplied by a 300W Xe lamp, whole operation should be conducted under visible light (>420nm) irradiation, and the photocurrent density is measured during five light-on and light-off cycles operation, turning on and off the light source every 20 seconds. All the collected potential versus Ag/AgCl (E_Ag/AgCl_) values were converted to normal hydrogen electrode (NHE) potential (E_NHE_) by the formula E_NHE_ = E_Ag/AgCl_ + 0.197.

**DFT Calculation Computations** were performed using the Vienna Ab initio Simulation Package (VASP) along with the Projector Augmented Wave (PAW) approach, employing first-principles density functional theory (DFT) and molecular dynamics simulations. The Generalized Gradient Approximation (GGA) framework was utilized to handle the exchange-correlation functional, specifically employing the Perdew-Burke-Ernzerhof (PBE) functional. The long-range van der Waals interactions are accounted for through the DFT-D3 approach. We utilized a plane wave basis set with an energy cutoff of 500 eV, and performed geometric relaxation until the atomic forces reached a threshold below 0.03 eV/Å. The Brillouin zone was sampled using a k-point grid with dimensions of 2 × 2 × 1. To ensure strict uniformity, computations were conducted until the energy convergence threshold reached a value lower than 10^-5^ eV. To prevent the interaction between periodic structures and ensure their effective isolation, a vacuum buffer with a length of 15 Å was introduced along the z-axis. The activation energy barrier is calculated by nudged elastic band method (CINEB).

 The free energy of the intermediates is calculated：

ΔG = ΔE_DFT_ + ΔZPE − TΔS (3)

Where ΔE_DFT_, ΔZPE and ΔS are the changes of the reaction energy obtained from DFT calculations, zero-point energy, and the changes of entropy from the initial state to the final state, respectively. T is temperature and the T of 298.15 K was used in all computations.


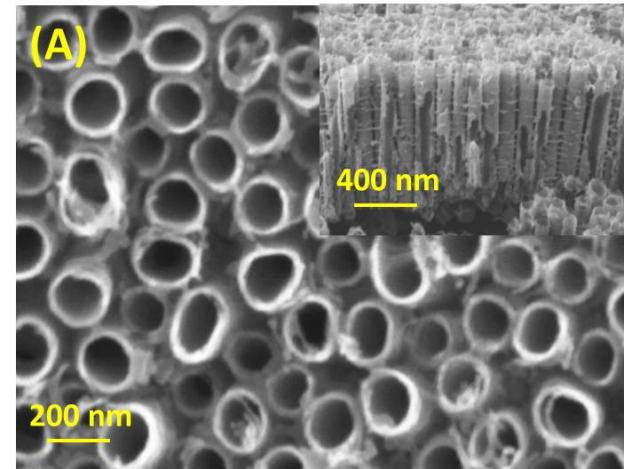


**Figure S1.** SEM images of TiO_2_ NTs (the inset is the cross of TiO_2_ NTs)


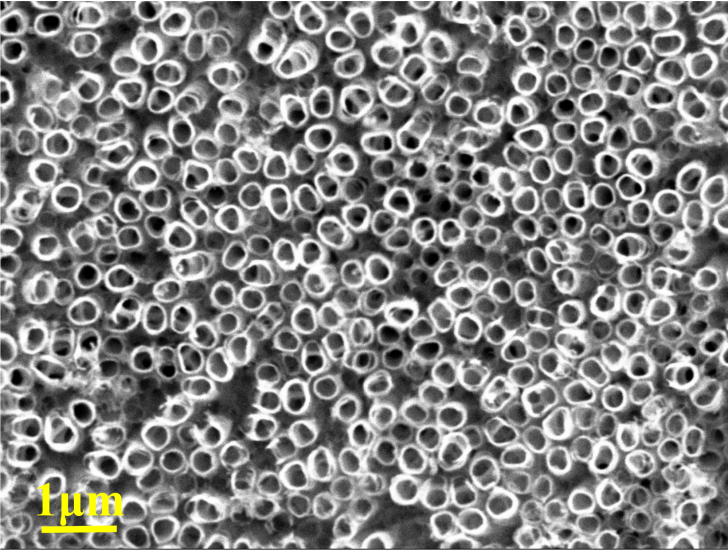

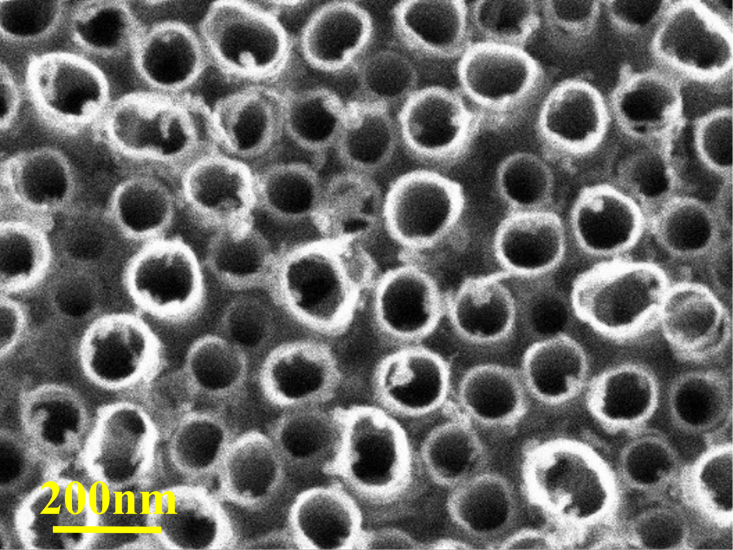


**(B)**

**(A)**

**Figure S2.** SEM images of the CdTe@TiO_2_ NTs (A) in 1um and (B) in 200 nm scale


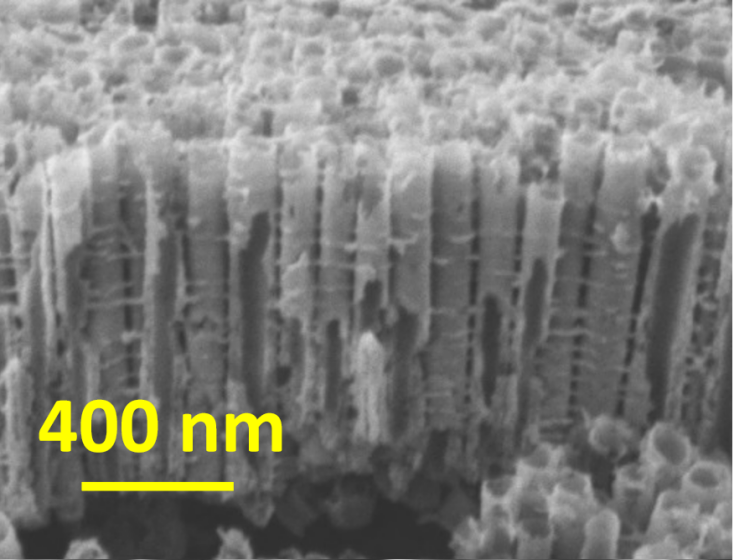


**Figure S3.** FSEM images of cross of CdTe@TiO_2_ NTs


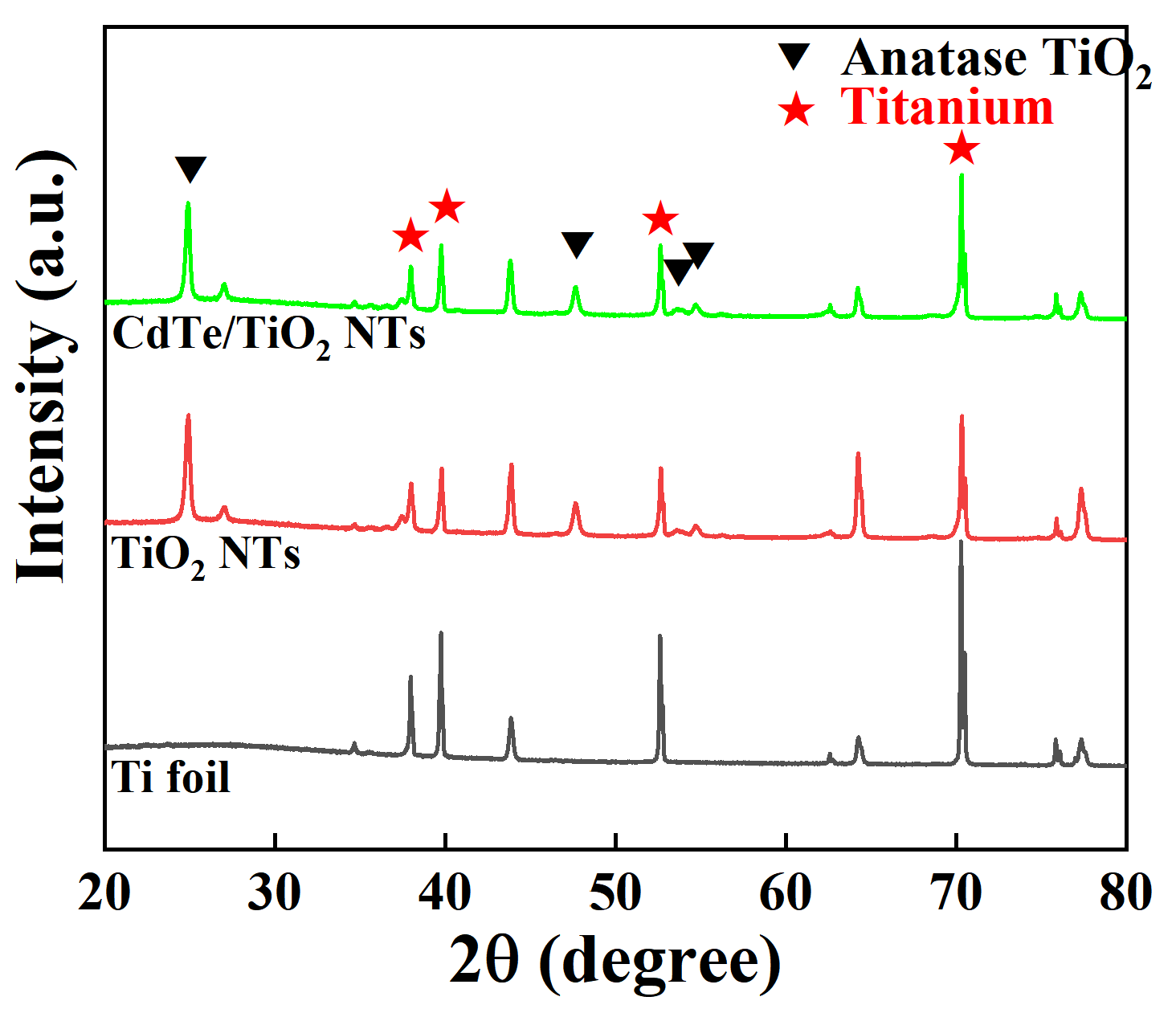


**Figure S4.** XRD pattern of as-synthesized TiO_2_ NTs and CdTe@TiO_2_ NTs


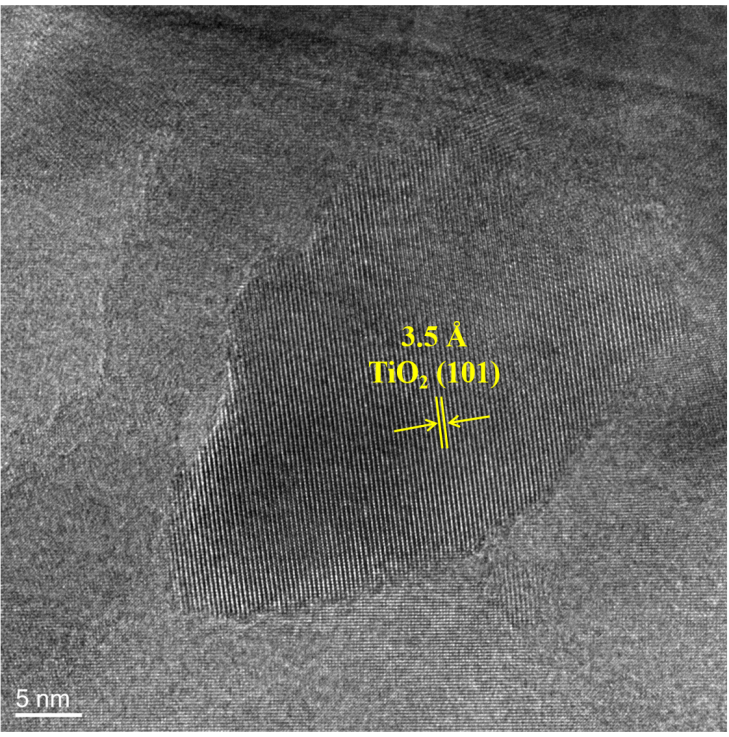

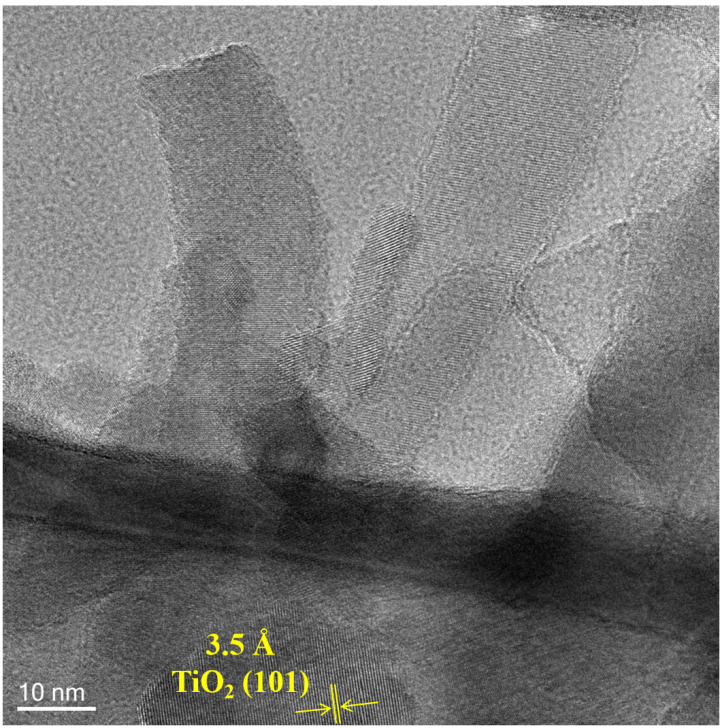


**(B)**

**(A)**

**Figure S5.** HRTEM images observation (A) in 5 nm and (B) in 10 nm for CdTe@TiO_2_ NTs


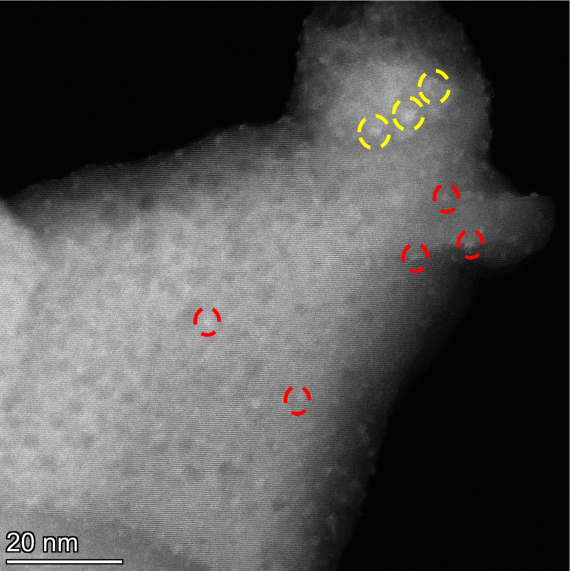

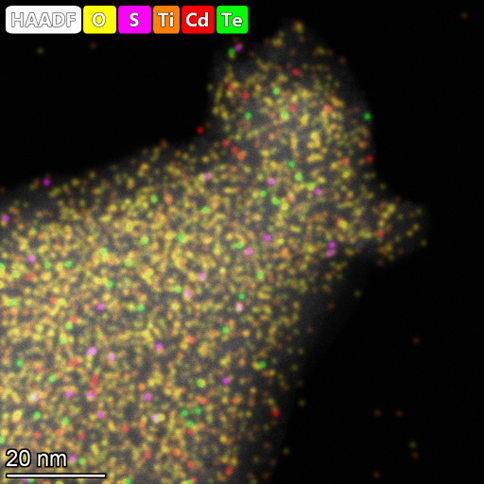


**(B)**

**(A)**


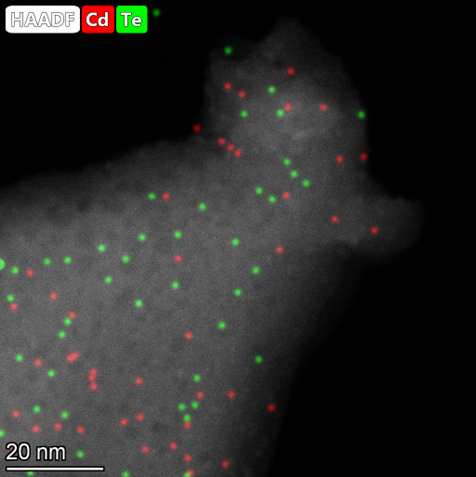


**(C)**

**Figure S6.** (A) HAADF-STEM, (B) Energy dispersive X-ray spectra of fresh CdTe@TiO_2_ NTs (C) EDX spectra of individual Cd and Te elements (single atomic Cd/Te sites high-lighted by red circles; Cd/Te clusters high-lighted by yellow circles)


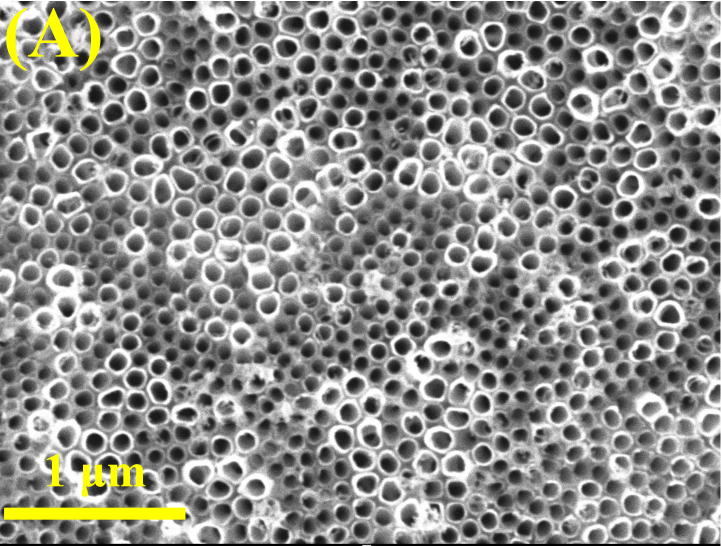

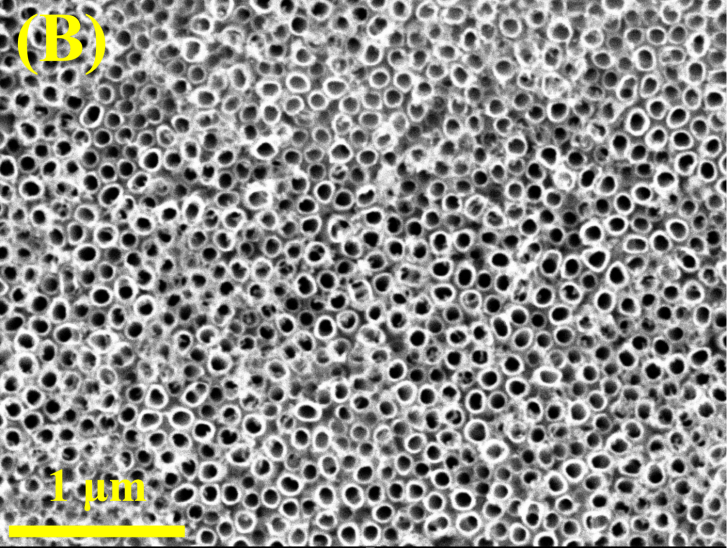


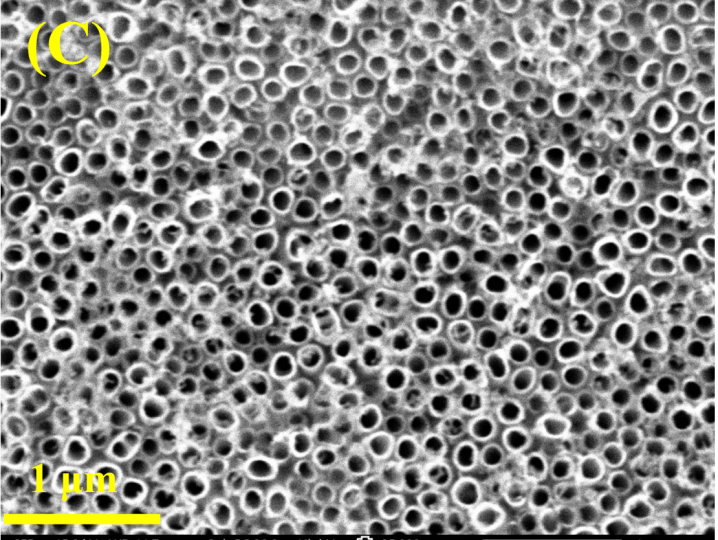

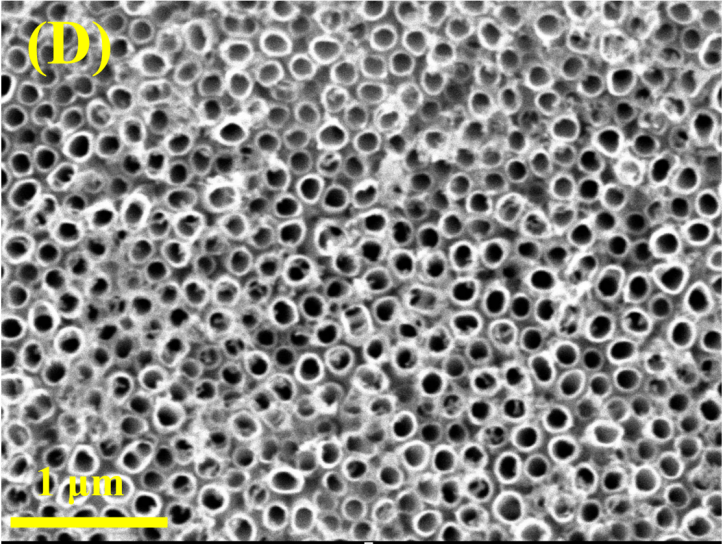


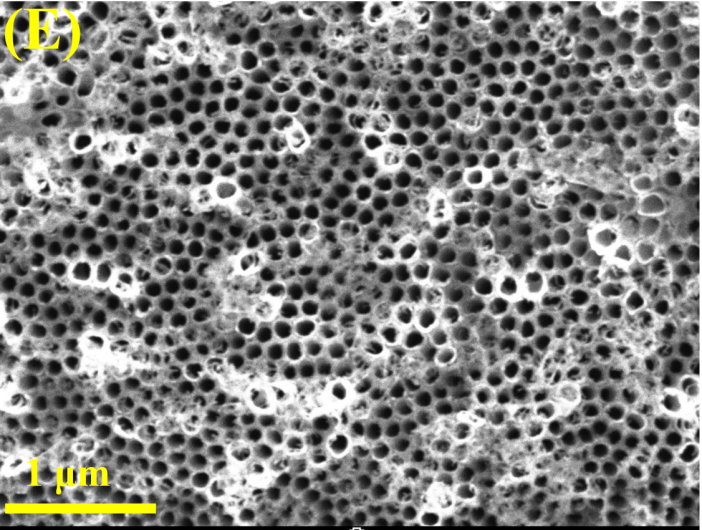


**Figure S7.** The SEM for (A) 1^st^ CdTe@TiO_2_ NTs, (B) 2^nd^ CdTe@TiO_2_ NTs, (C) 3^rd^ CdTe@TiO_2_ NTs, (D) 4^th^ CdTe@TiO_2_ NTs and (E) and 5^th^ CdTe@TiO_2_ NTs


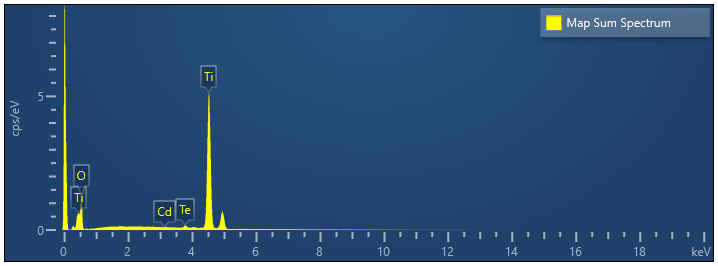


**(A)**


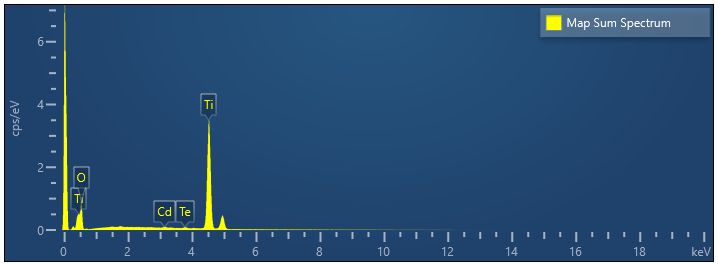

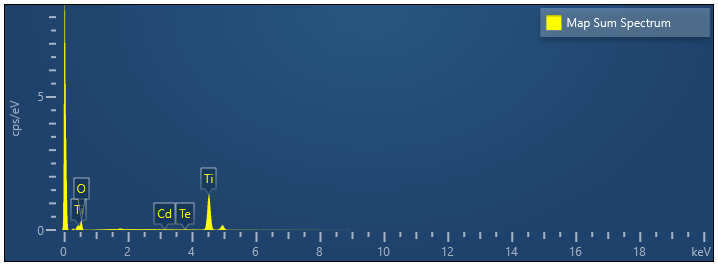


**(C)**

**(B)**


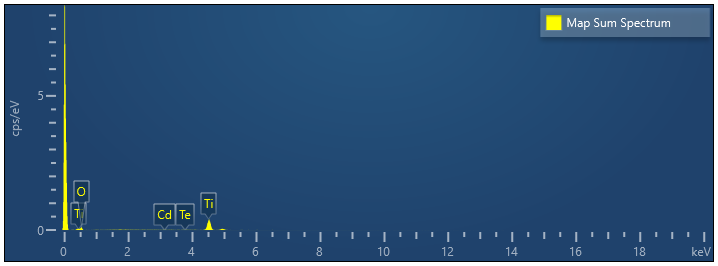


**(D)**

**Figure S8.** The corresponding EDS spectrum for (A) CdTe@TiO_2_ NTs, (B) 1^st^ CdTe@TiO_2_ NTs, (C) 3^rd^ CdTe@TiO_2_ NTs, (D) 5^th^ CdTe@TiO_2_ NTs


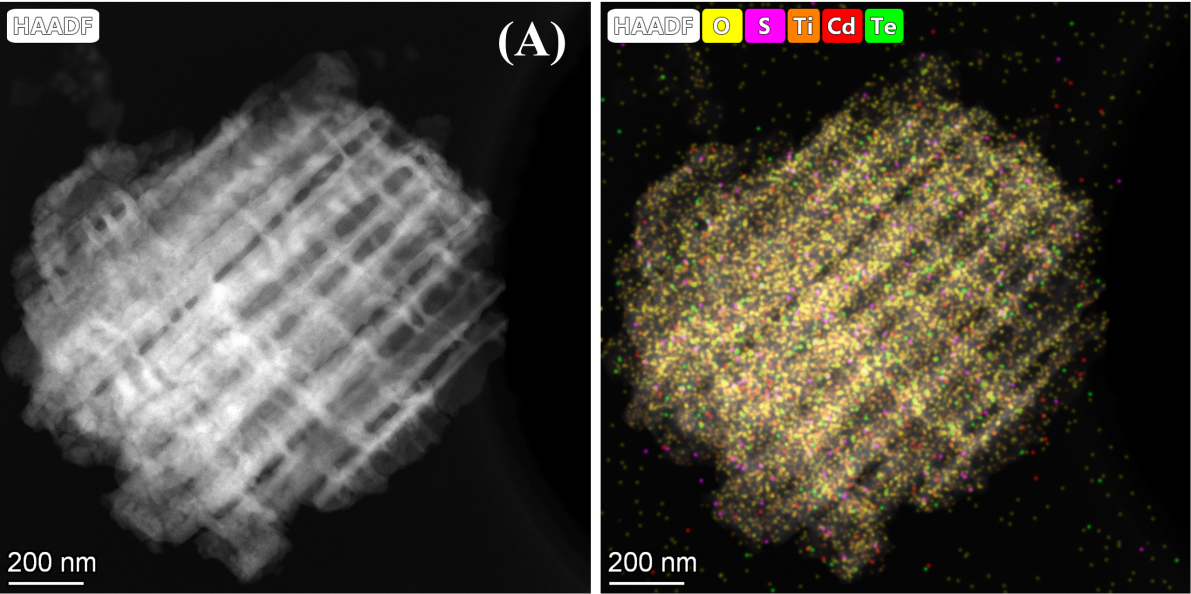


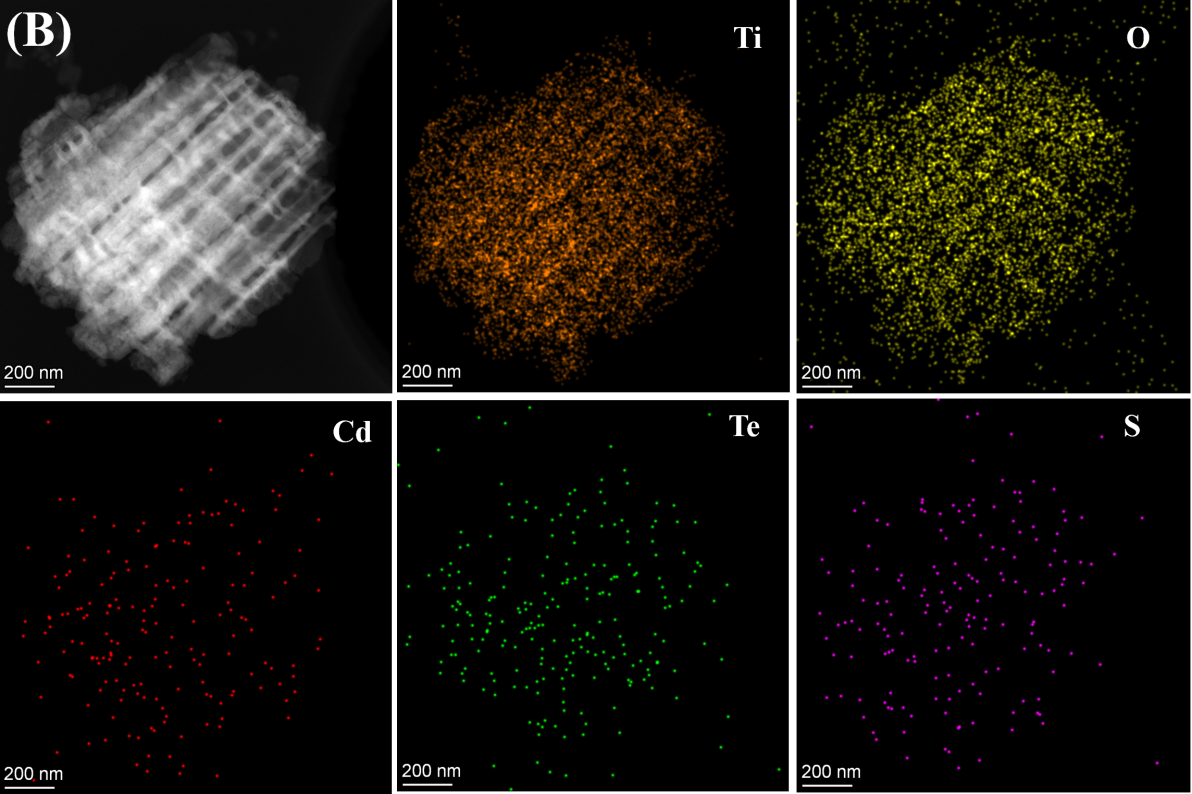


**Figure S9.** HAADF-STEM with energy dispersive X-ray spectra of (A) 5^th^ CdTe@TiO_2_ NTs; (B) Ti, O, Cd, Te elements in 5^th^ CdTe@TiO_2_ NTs

**(C)**

**(B)**

**(A)**


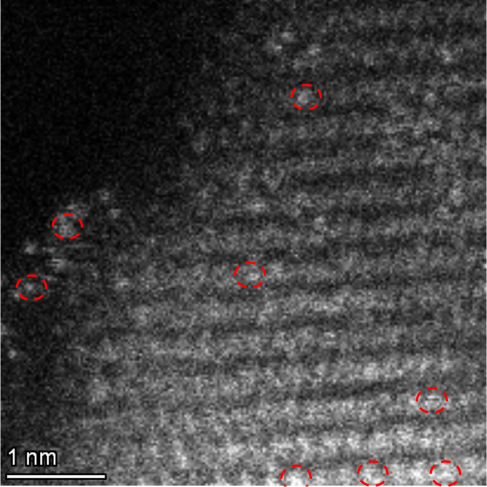

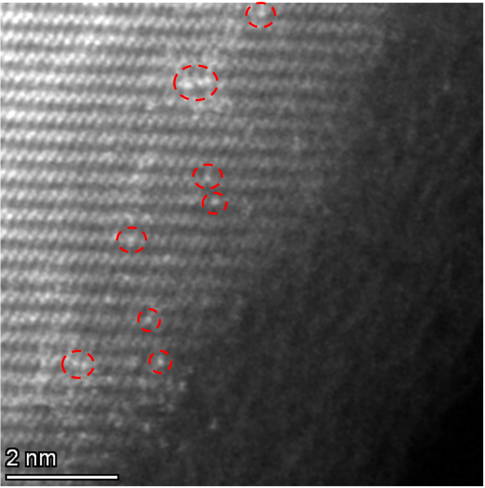

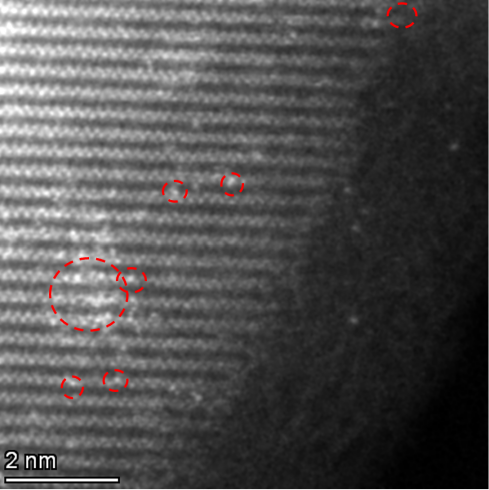


**(E)**

**(D)**


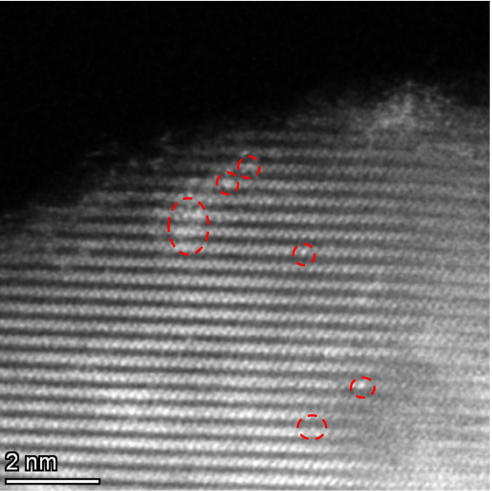

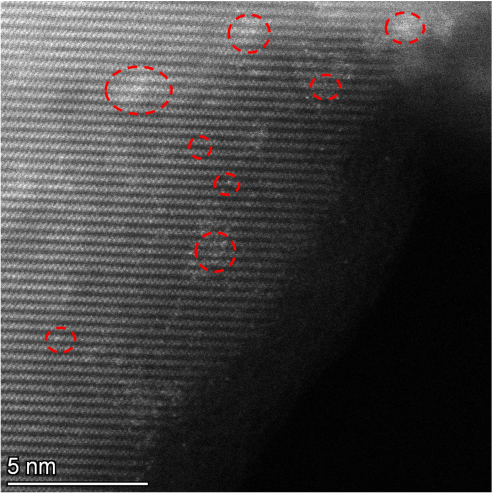


**Figure S10.** HAADF-STEM images of 5^th^ CdTe@TiO_2_ NTs (A) in 1nm (B-D) in 2nm (E) in 5 nm (single atomic Cd sites high-lighted by red circles; Cd clusters high-lighted by yellow circles)


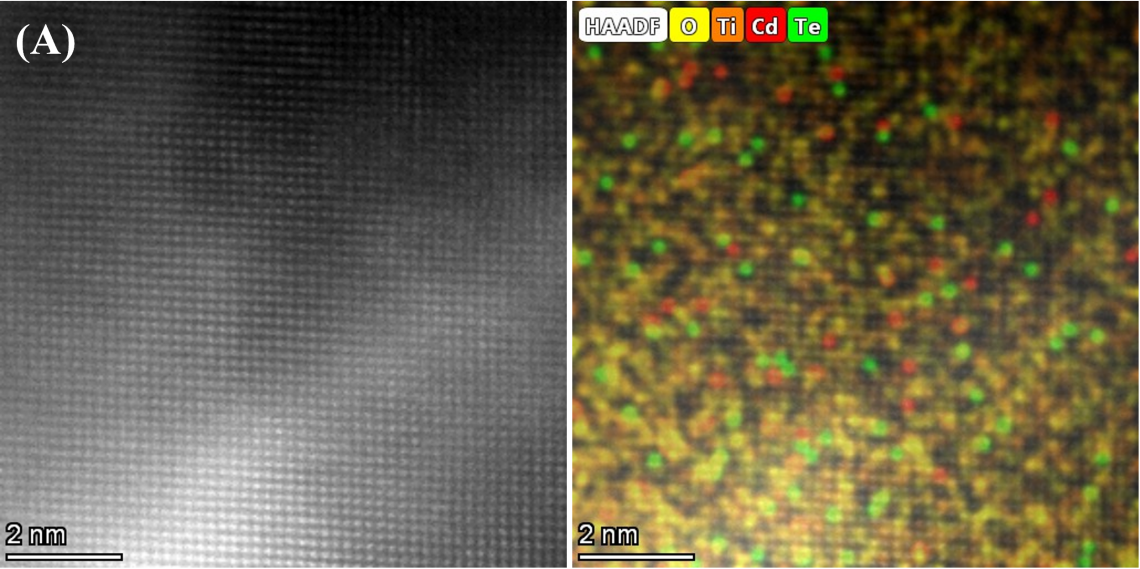


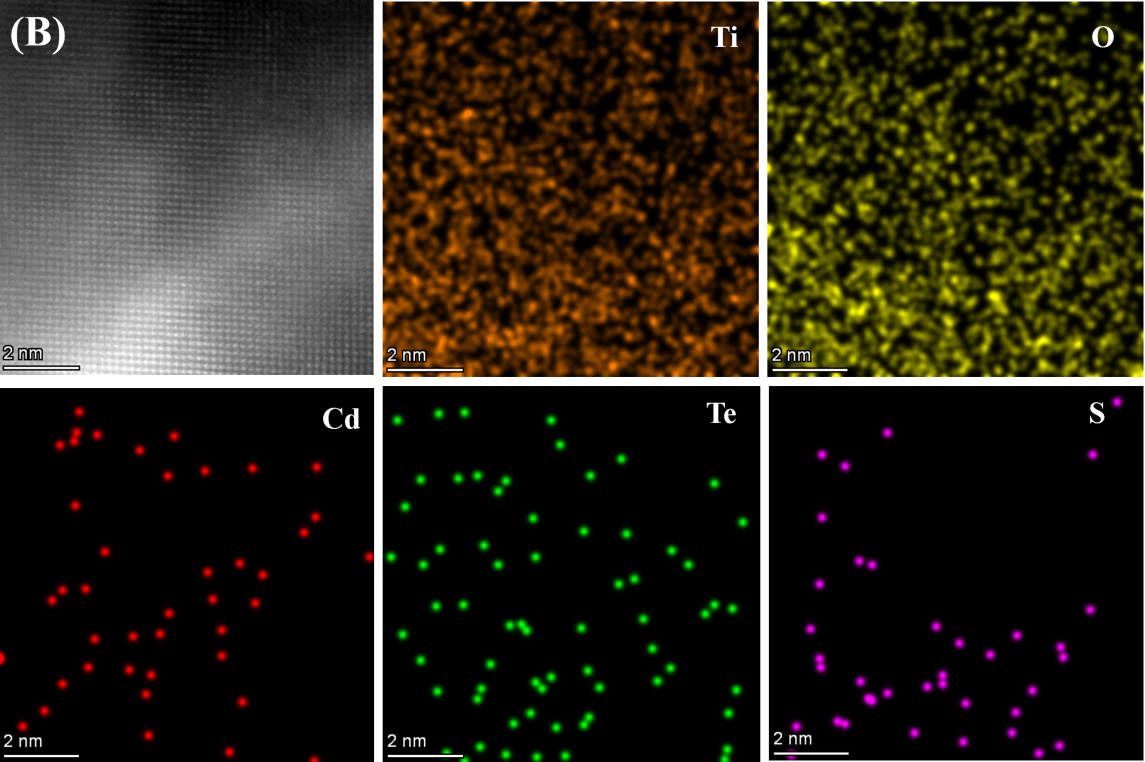


**Figure S11.** HAADF-STEM with energy dispersive X-ray spectra of (A) 5^th^ CdTe@TiO_2_ NTs; (B) Ti, O, Cd, Te elements in 5^th^ CdTe@TiO_2_ NTs


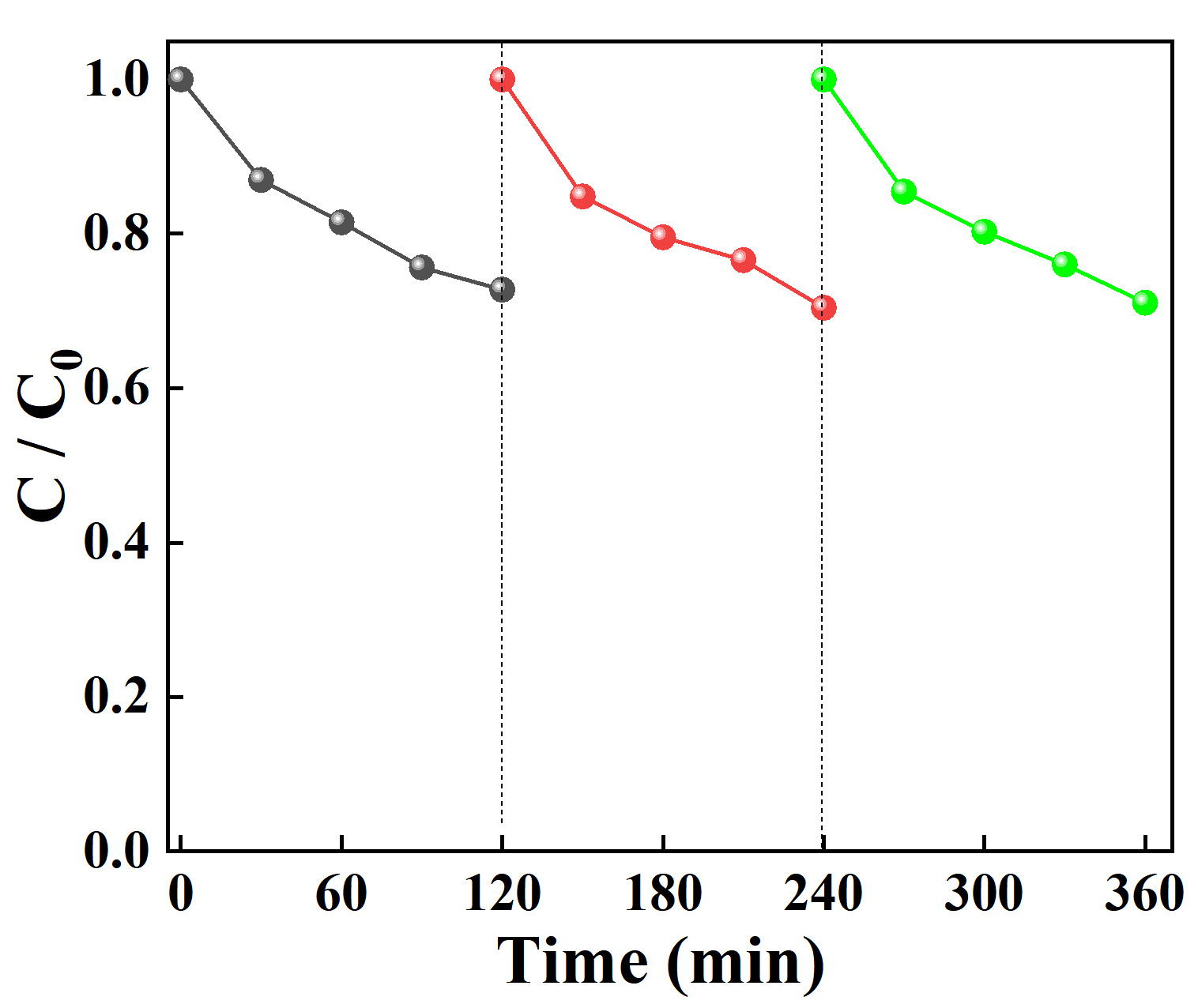


**Figure S12.** Recycling test for toluene degradation curve on TiO_2_ NTs


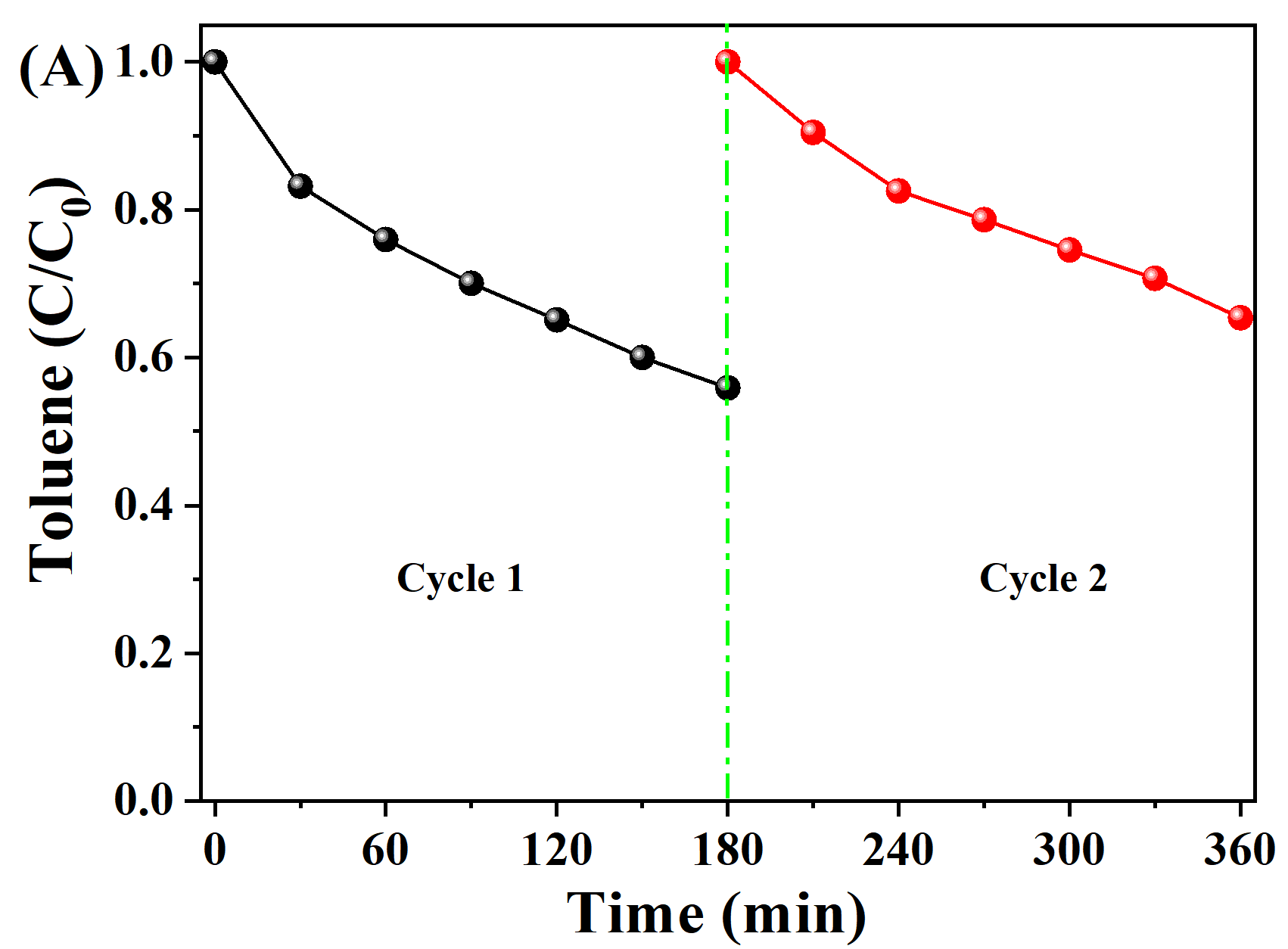


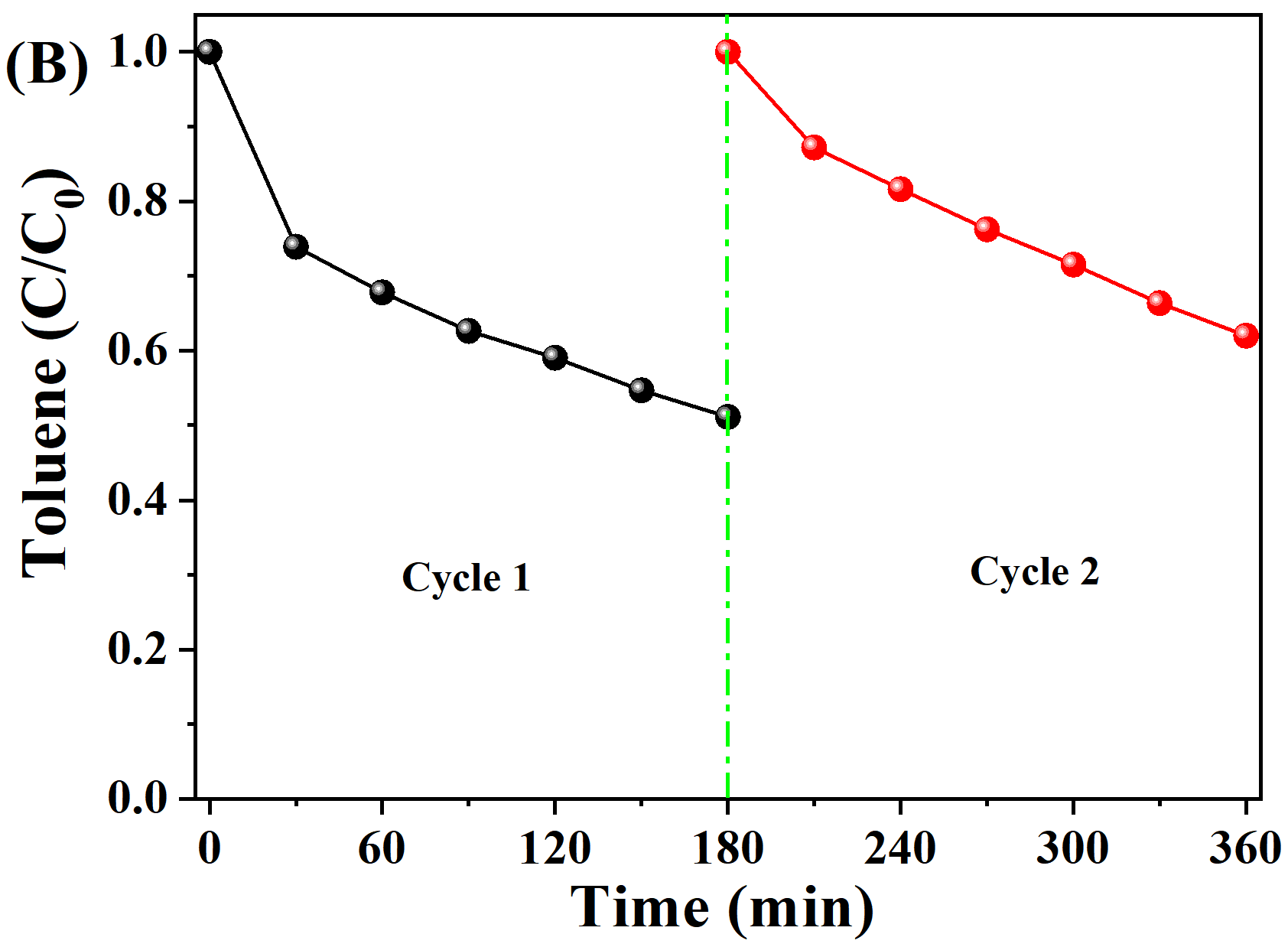


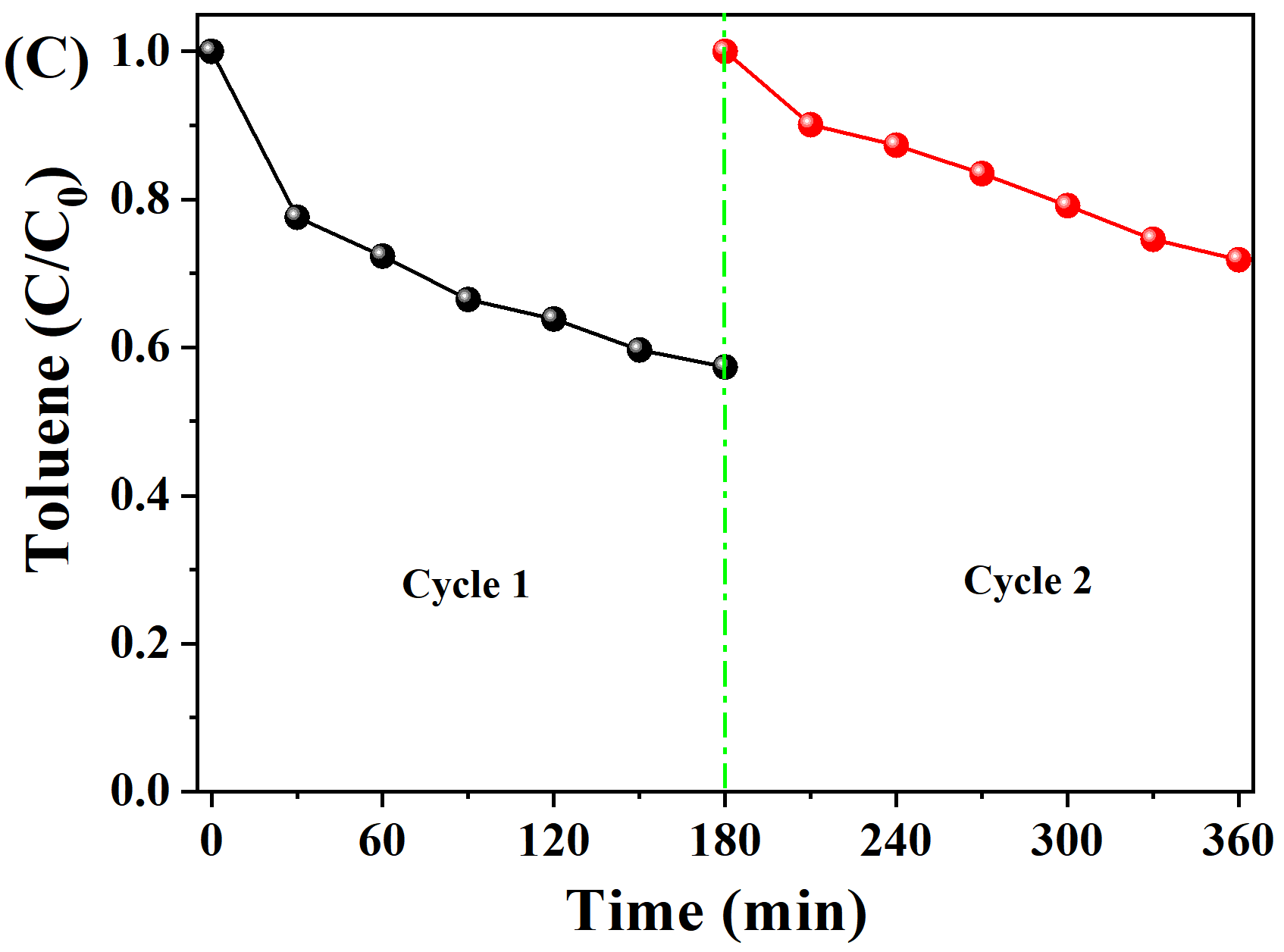


**Figure S13.** Recycling test for toluene degradation curve on (A) CdTe@TiO_2_ NTs (HNO_3_ served as pH value regulators) (B) TiO_2_ NTs (H_2_SO_4_ served as pH value regulators without Cd/Te during electrochemical deposition process), (C) CdTe@TiO_2_ NTs (pH value in electrolyte did not be regulated)


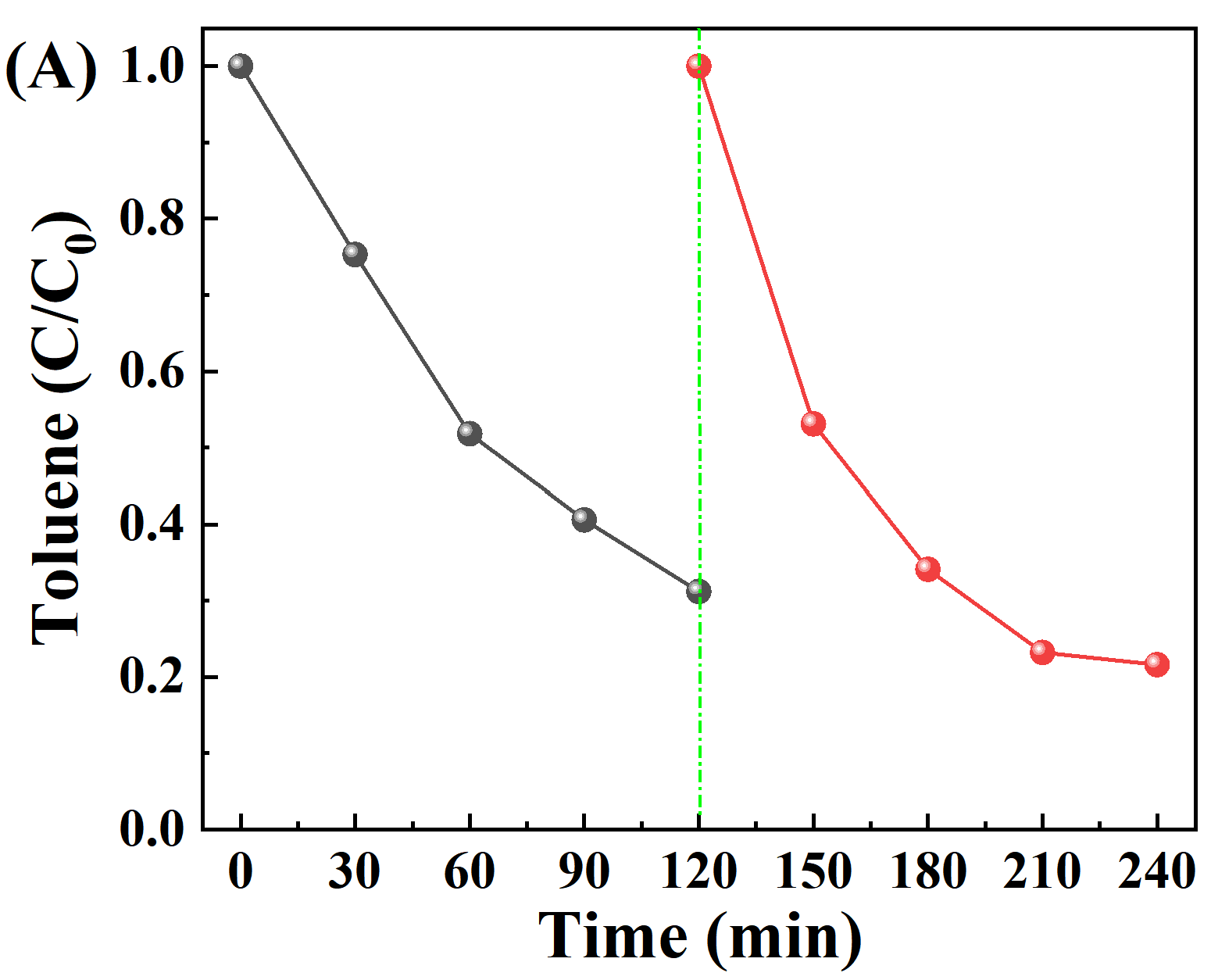


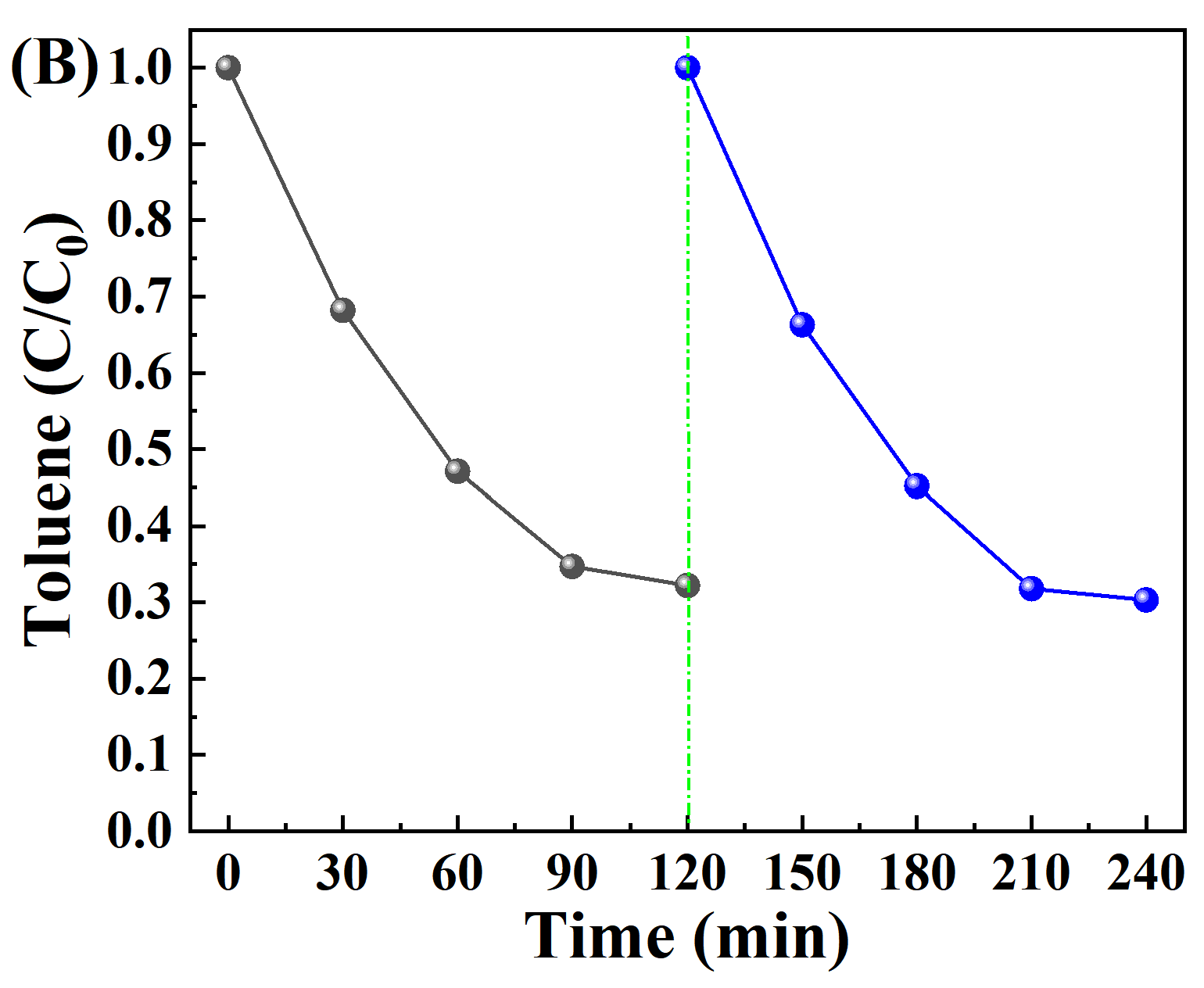


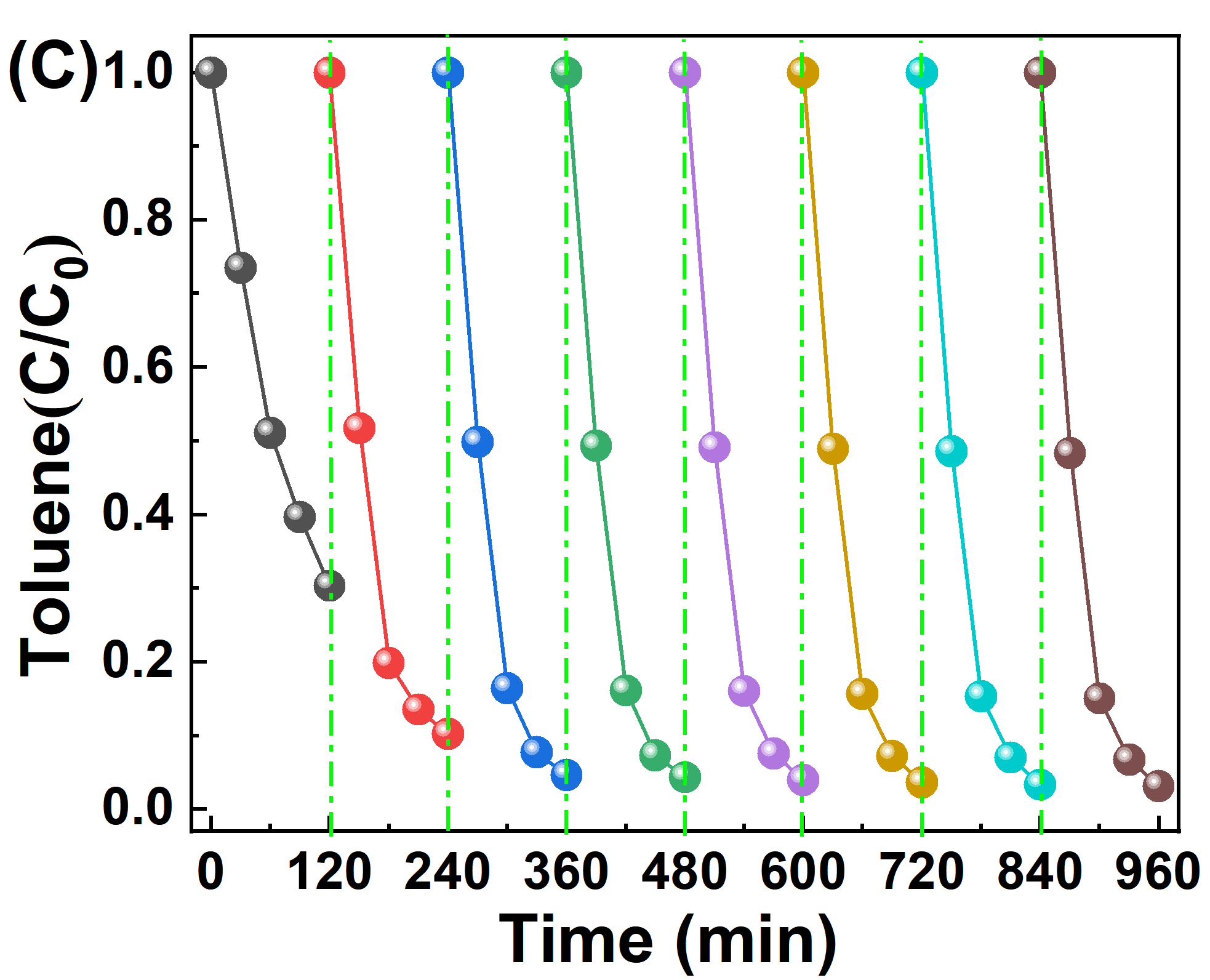


**Figure S14.** Recycling test for toluene degradation curve on (A) Cd@TiO_2_ NTs and (B) Te@TiO_2_ NTs; (C) Photocatalytic performance for 8^th^ recycling test of toluene degradation curve on the CdTe@TiO_2_ NTs


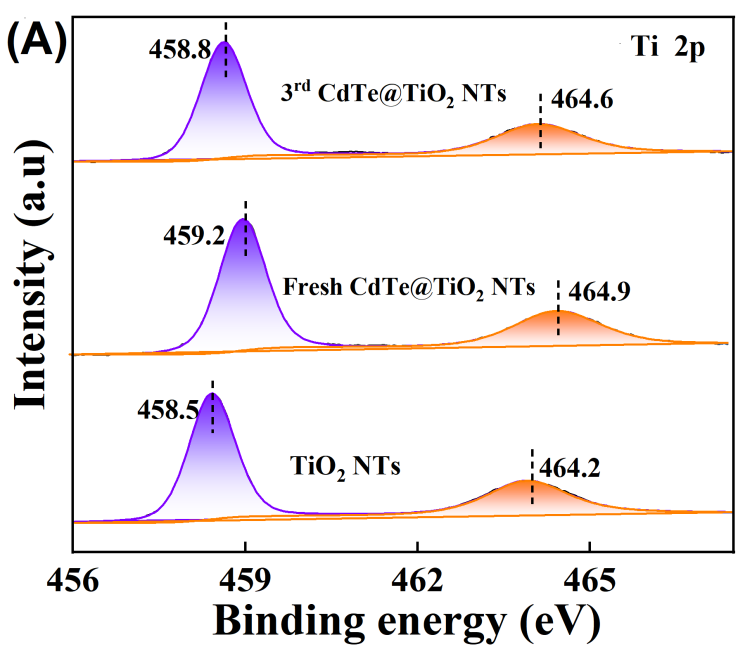

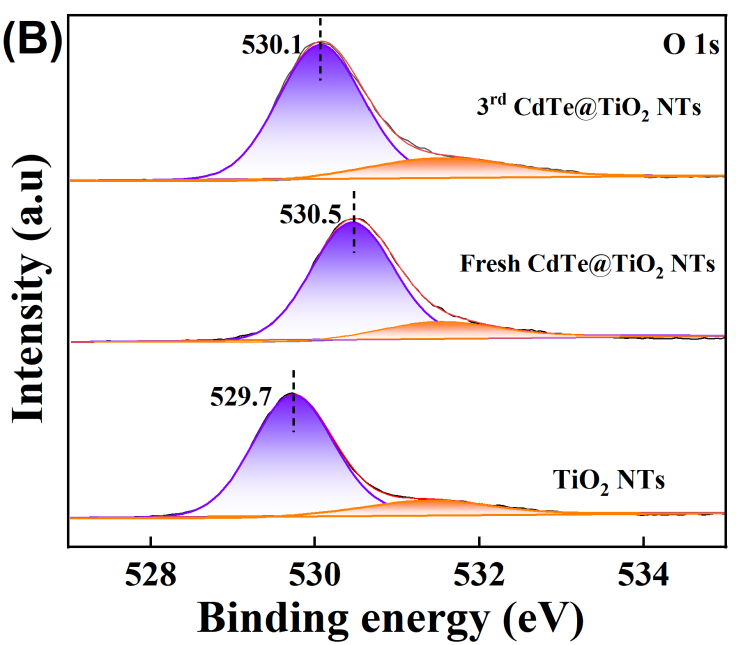


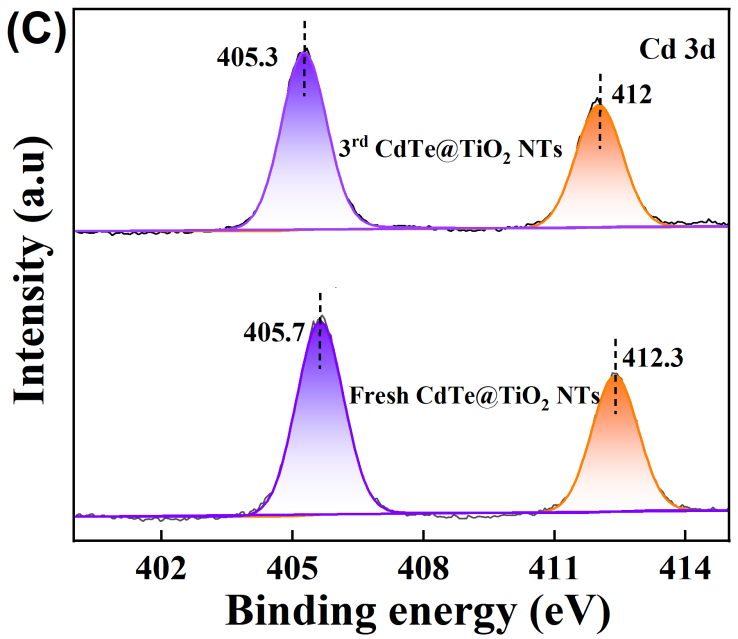

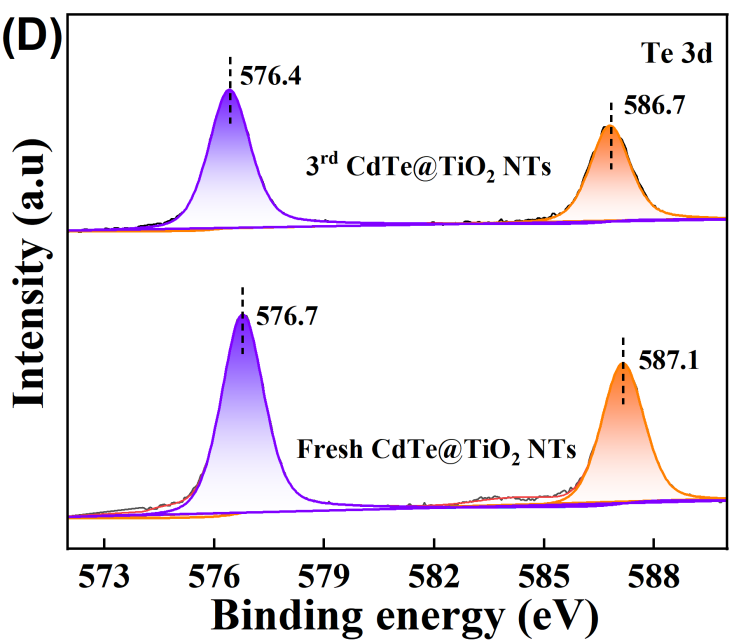


**Figure S15.** XPS peak position spectra of photocatalysis: (A) Ti 2p, (B) O 1s, (C) Cd 3d, (D) Te 3d of as-synthesized TiO_2_ NTs, CdTe@TiO_2_ NTs and recycled CdTe@TiO_2_ NTs with various repeating times


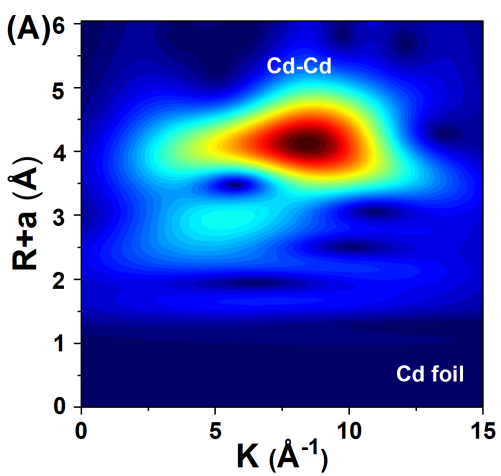

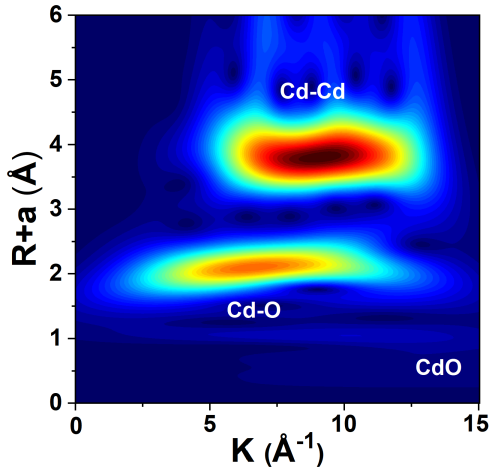

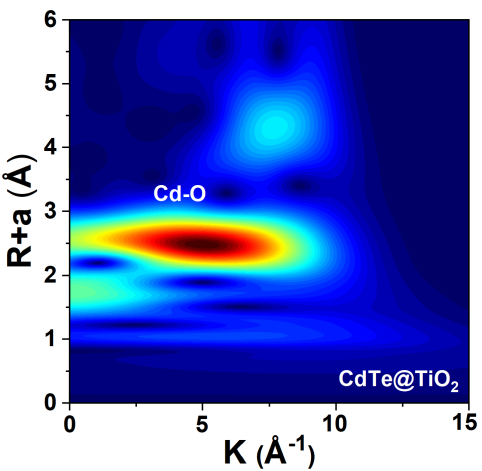


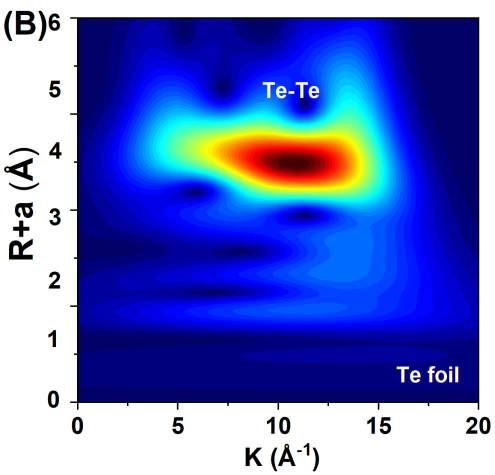

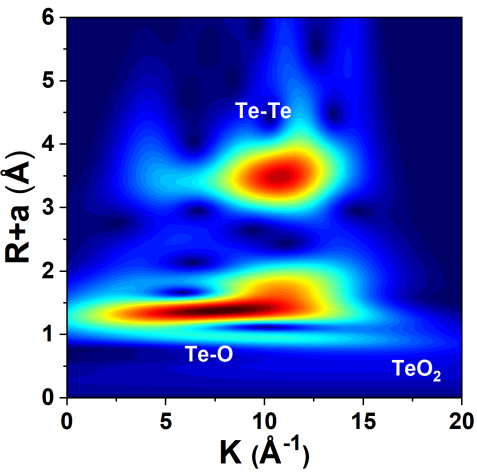

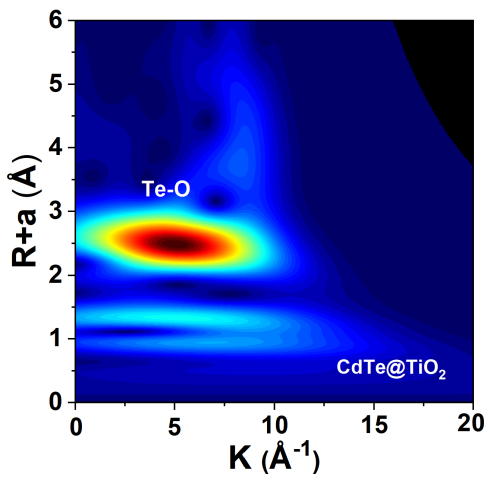


**Figure S16.** Atomic structure and coordination state investigation. (A) WT-EXAFS contour plots at the Cd K-edge of recycled CdTe@TiO_2_ NTs and reference; (B) WT-EXAFS contour plots at the Te K-edge of recycled CdTe@TiO_2_ NTs and reference


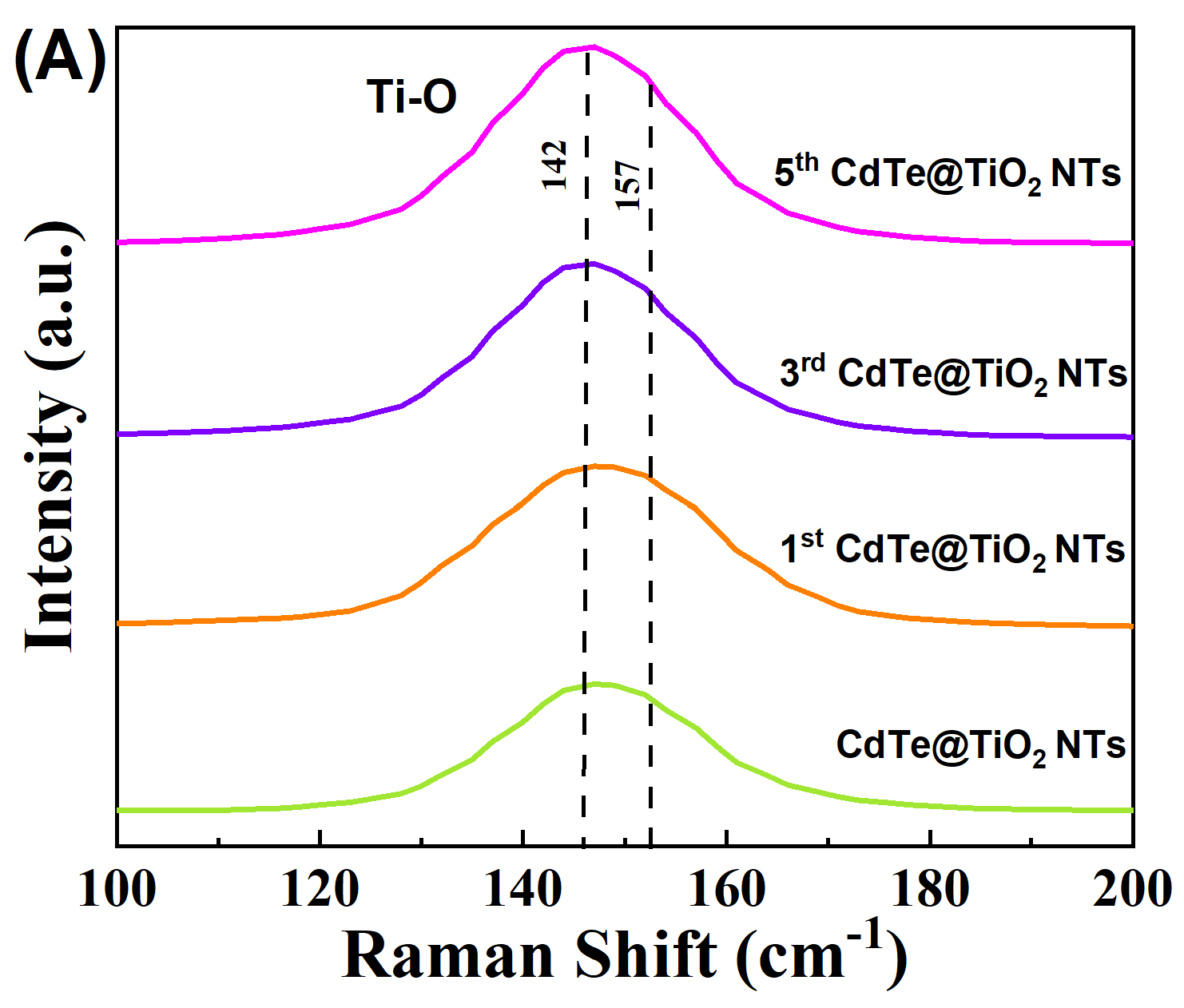


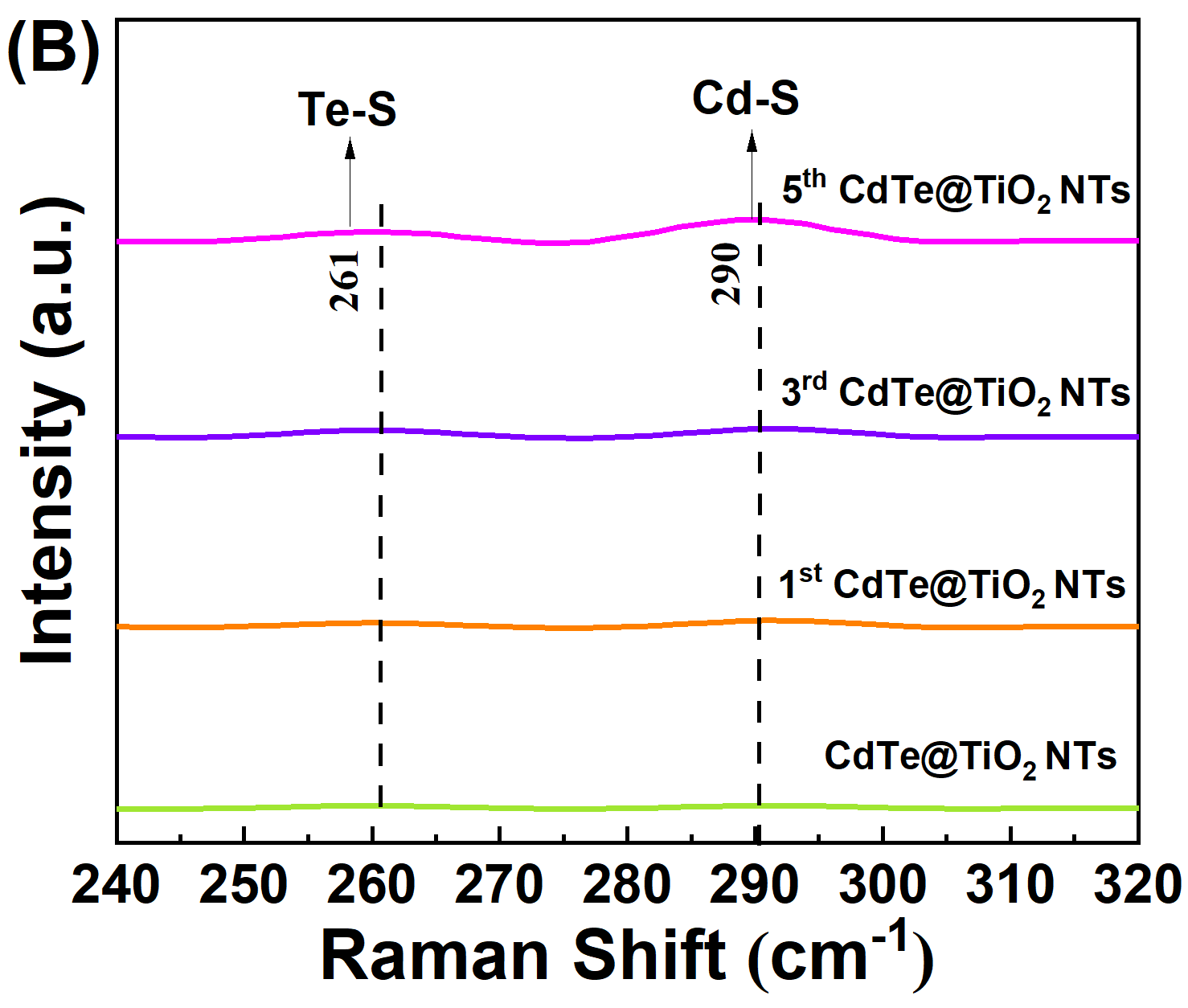


**Figure S17.** Raman spectra of as-synthesized TiO_2_ NTs, CdTe@TiO_2_ NTs and recycled CdTe@TiO_2_ NTs with various repeating times at the Raman shift between (A) 100-200 cm^-1^ and (B) 240-320 cm^-1^.


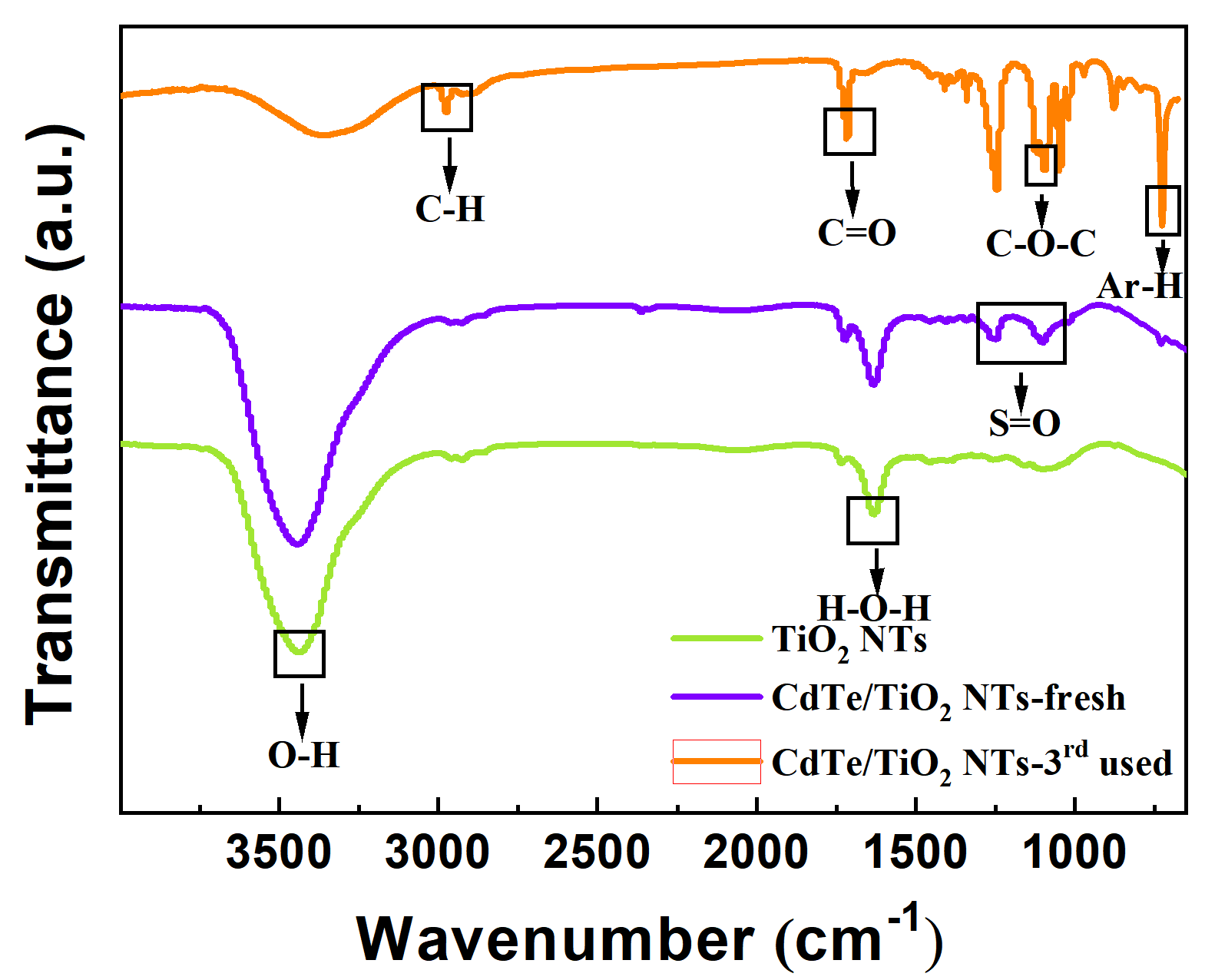


**Figure S18.** FT-IR measurement of gaseous toluene in PC reaction on TiO_2_ NTs, CdTe@TiO_2_ NTs and recycled CdTe@TiO_2_ NTs under visible light illumination (λ=420nm)


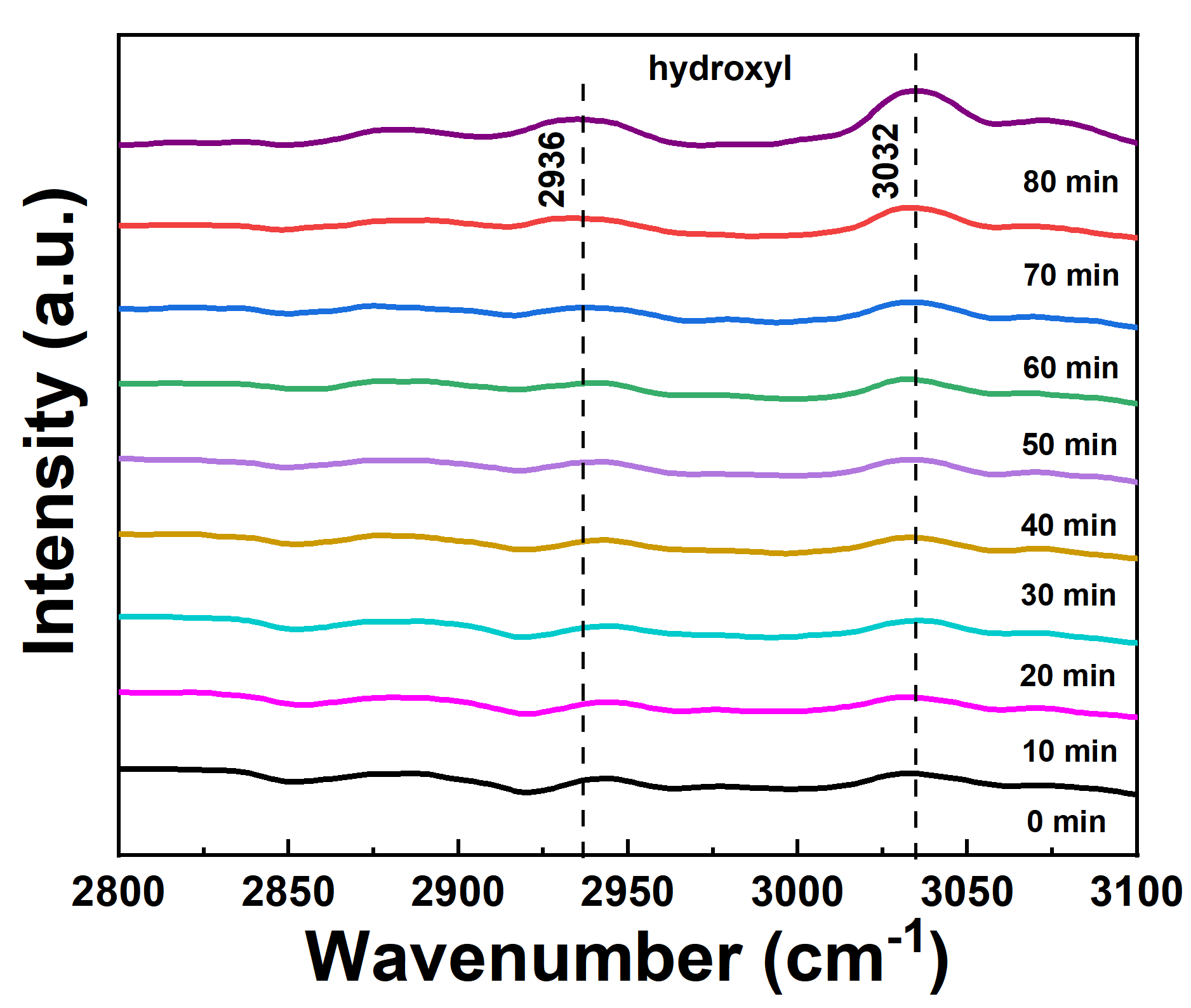


**Figure S19.** In-situ DRIFTS spectrum on CdTe@TiO_2_ NTs under visible light illumination (λ=420 nm) at gaseous toluene atmosphere at 2800-3100 cm^-1^


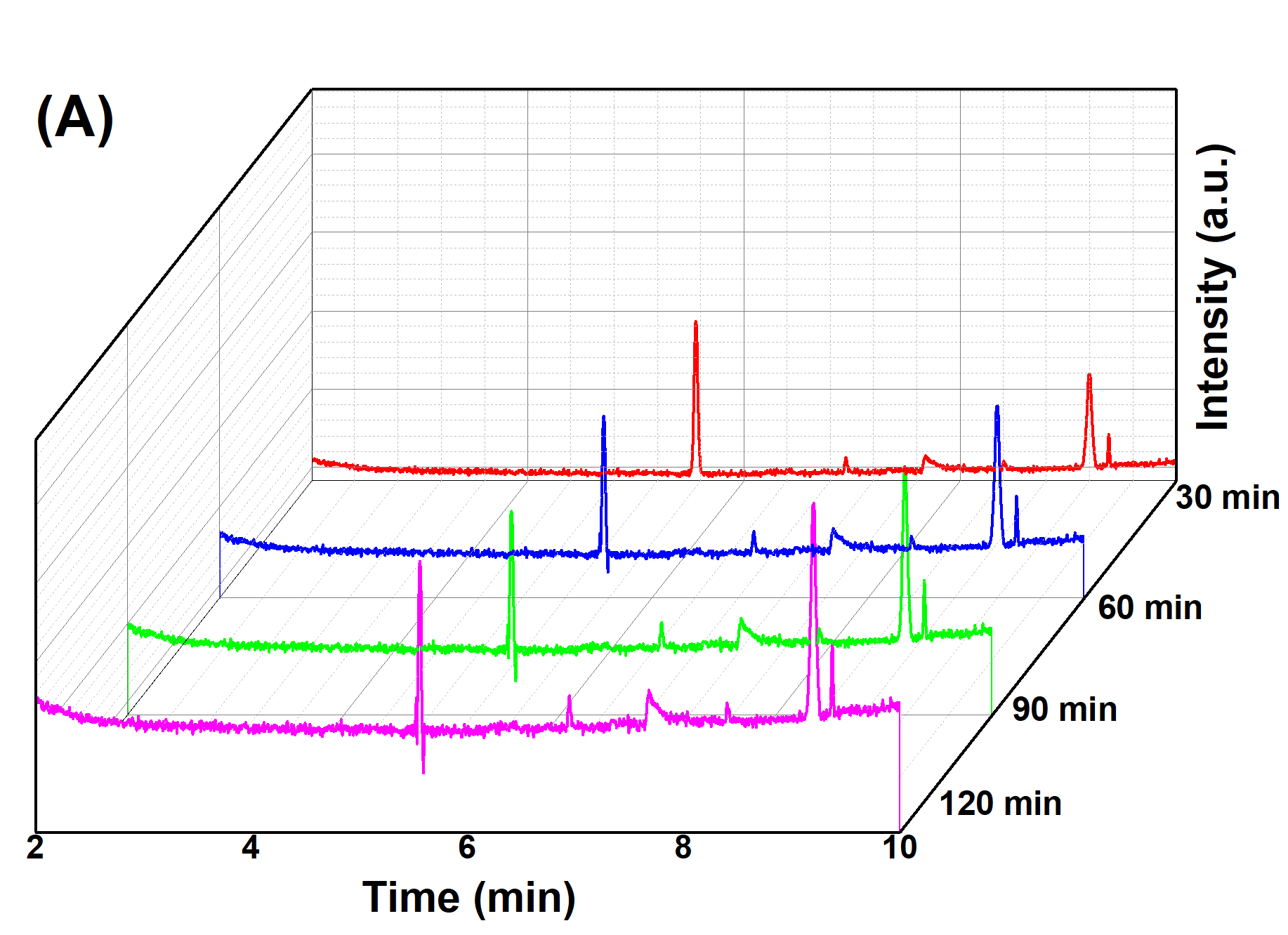


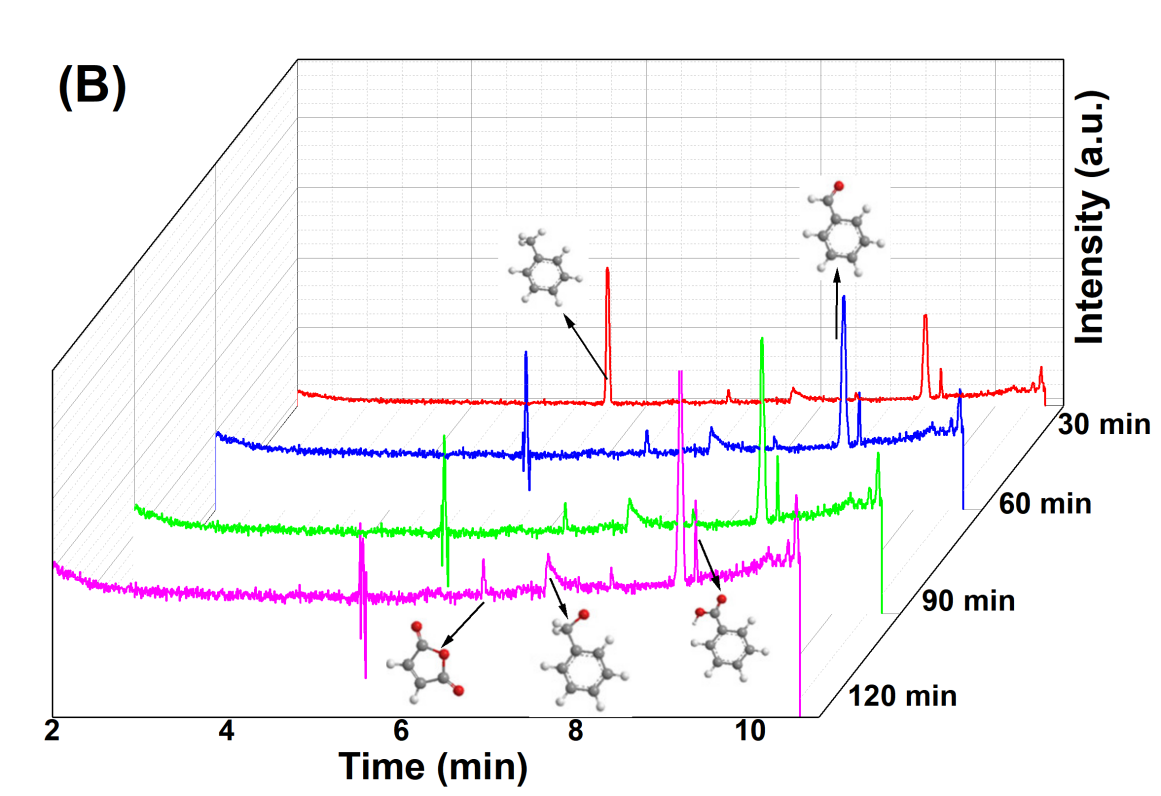


**Figure S20.** TD-GC/MS results of removal of toluene on (A) CdTe@TiO_2_ NTs and (B) 5^th^ CdTe@TiO_2_ NTs


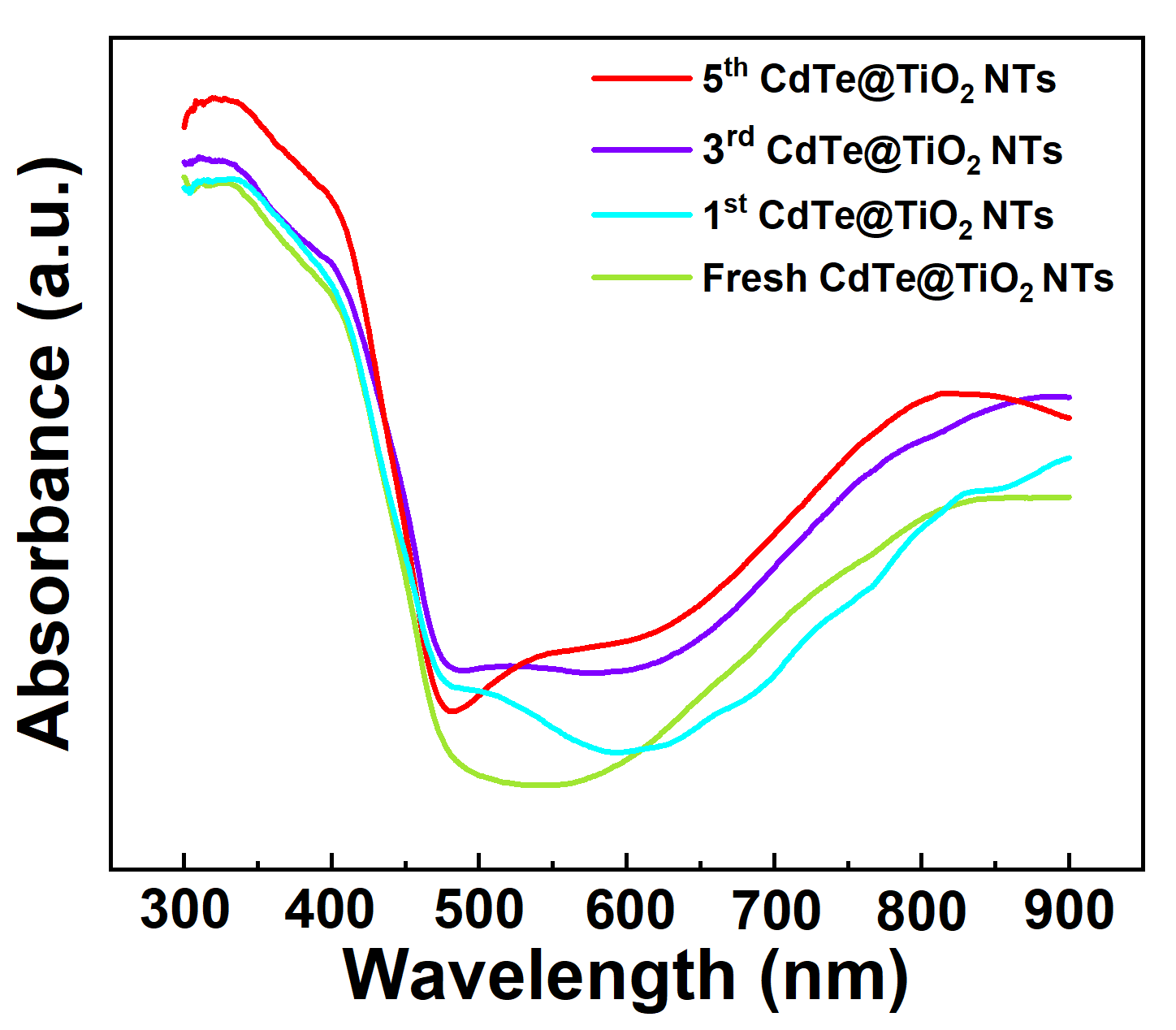


**Figure S21.** UV-vis absorption spectra for as-synthesized TiO_2_ NTs, CdTe@TiO_2_ NTs and recycled CdTe@TiO_2_ NTs with various repeating times


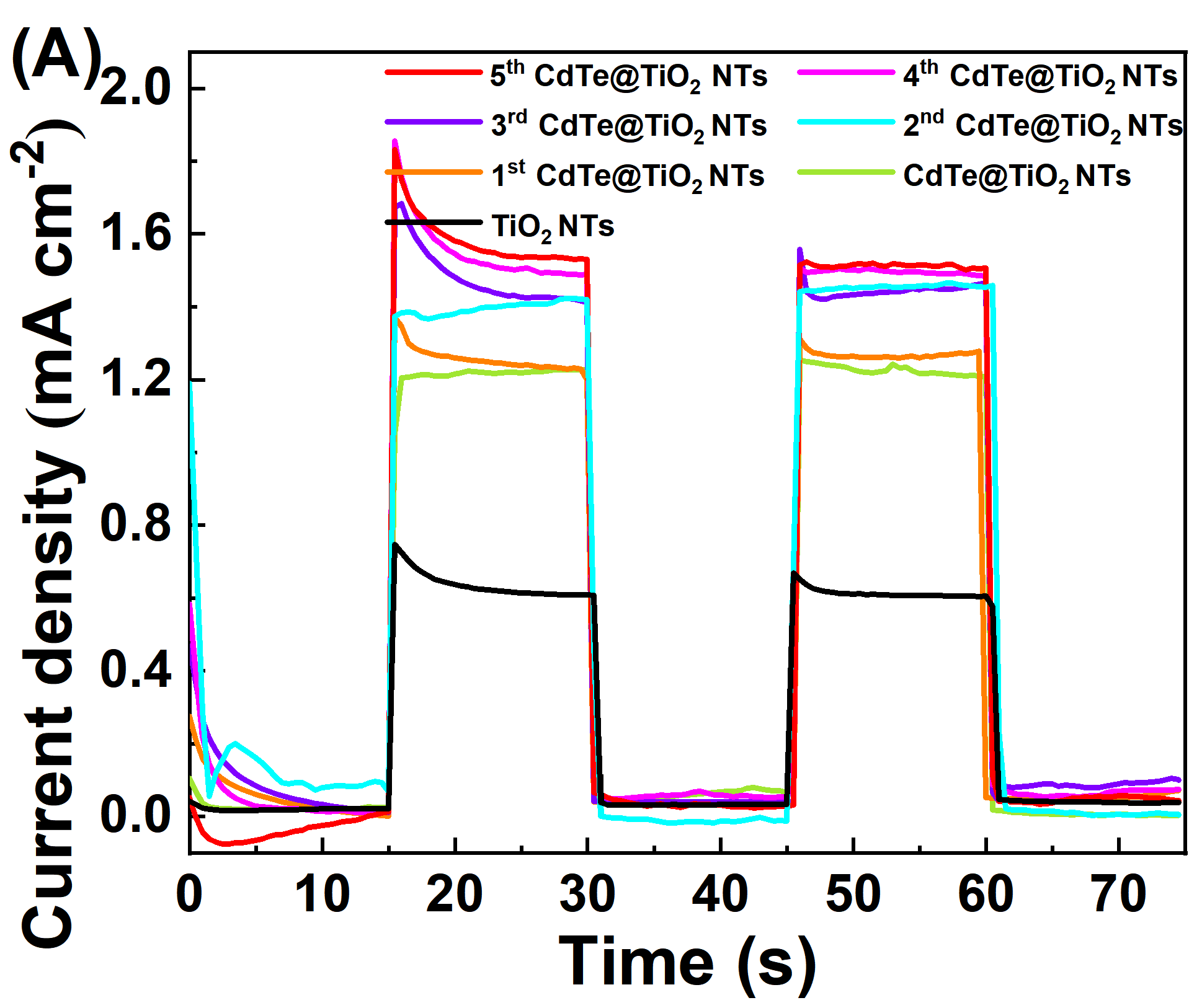


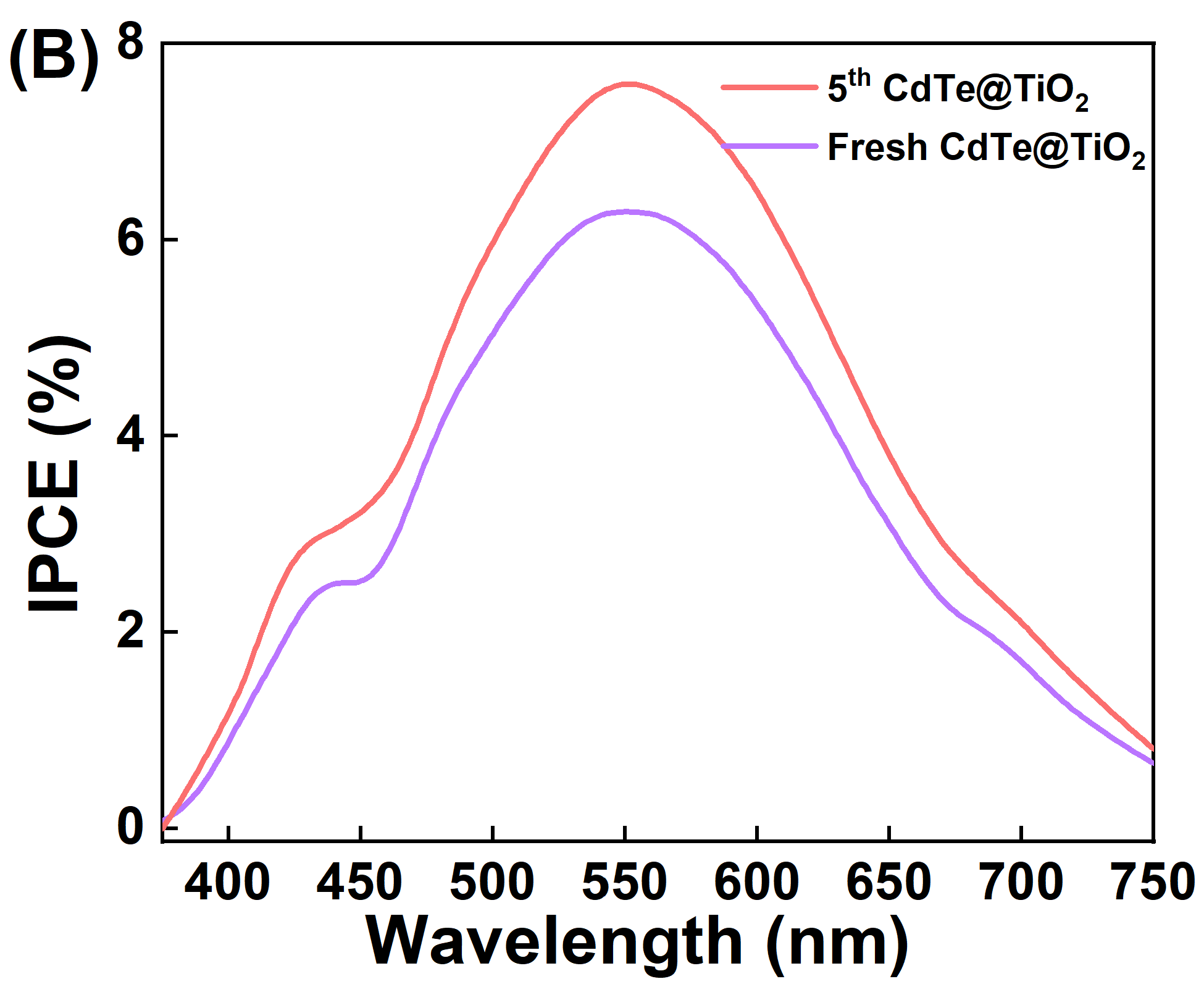


**Figure S22.** (A) Transient photocurrent density for the as-synthesized TiO_2_ NTs, CdTe@TiO_2_ NTs and recycled CdTe@TiO_2_ NTs; (B) The incident photon-to-electron conversion efficiency (IPCE) of samples


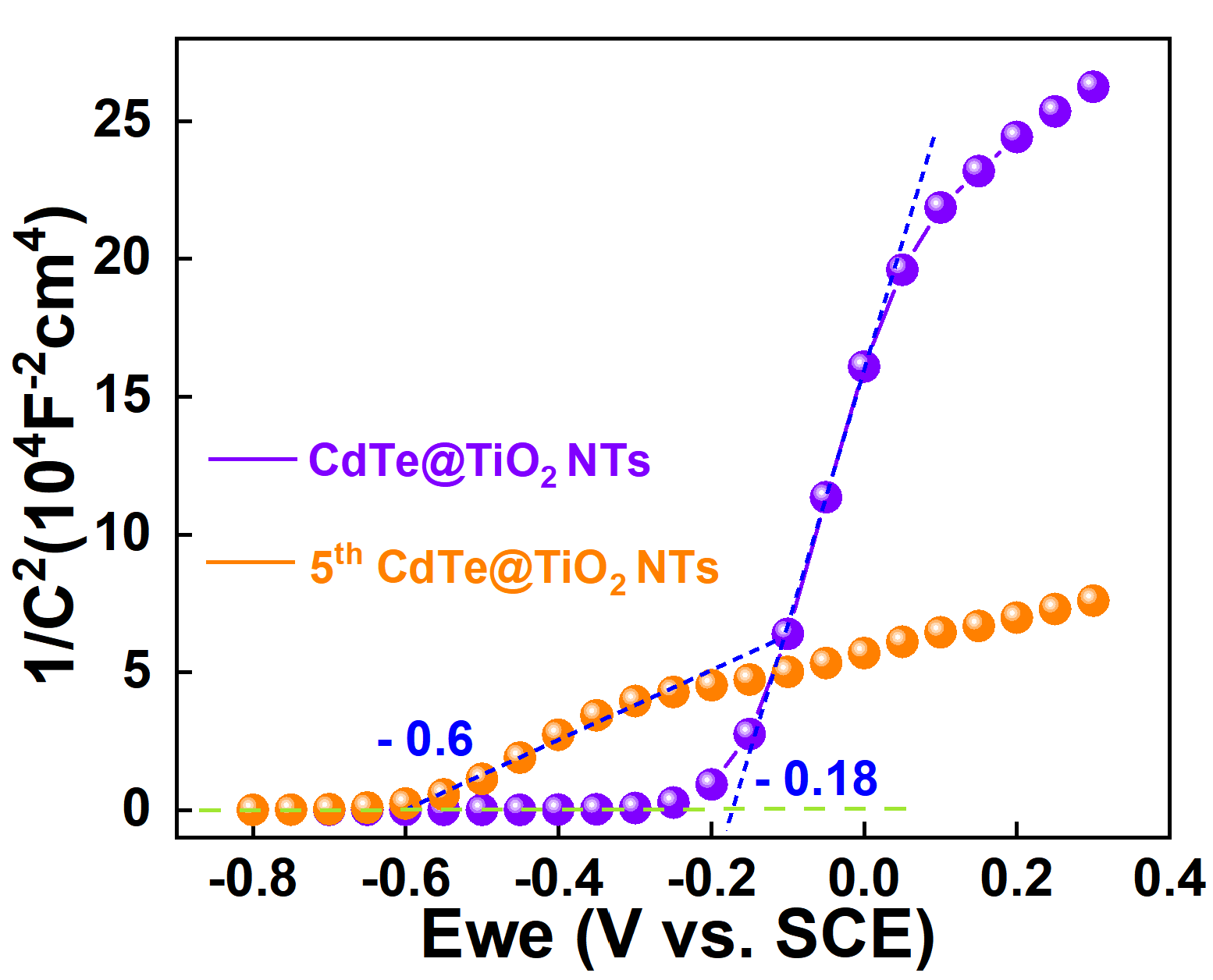


**Figure S23.** Mott−Schottky plots for as-synthesized fresh CdTe@TiO_2_ NTs and 5^th^ CdTe@TiO_2_ NTs


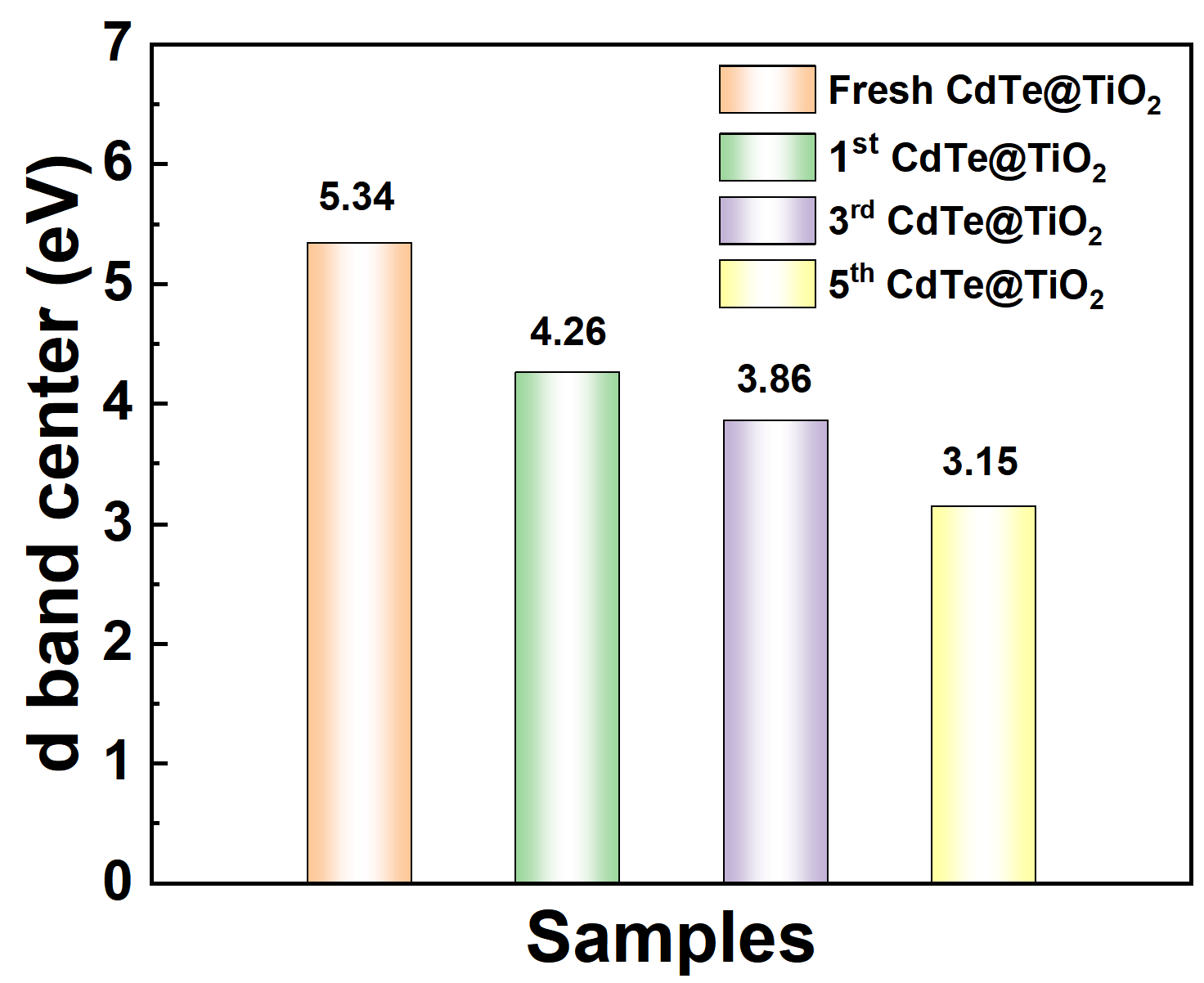


**Figure S24.** The d-band center absolute value of the catalysts calculated from high-resolution valence band XPS


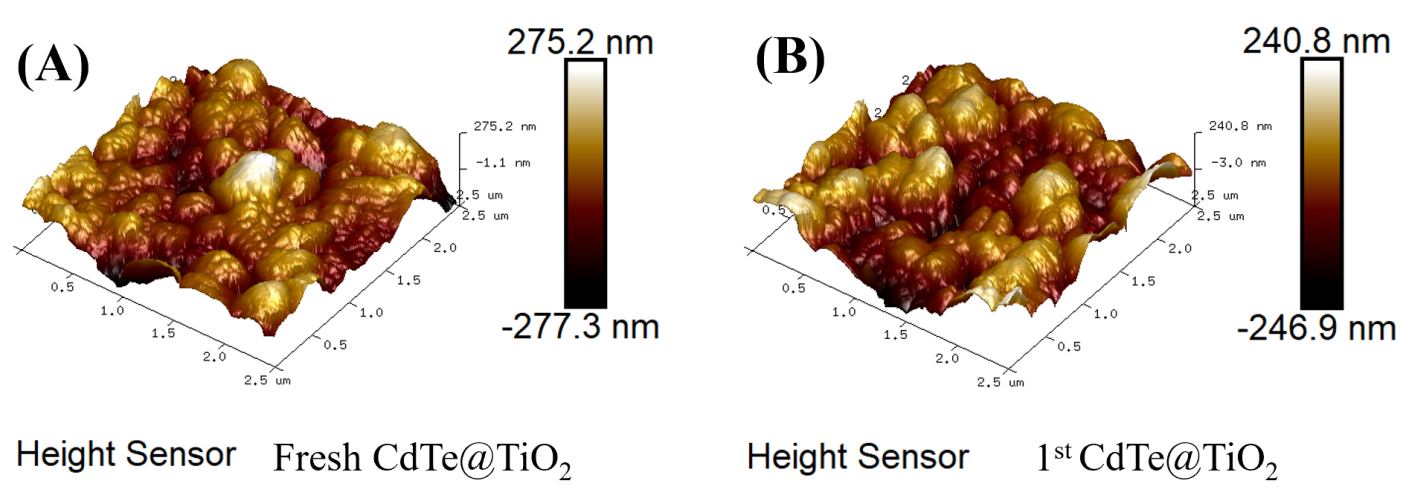


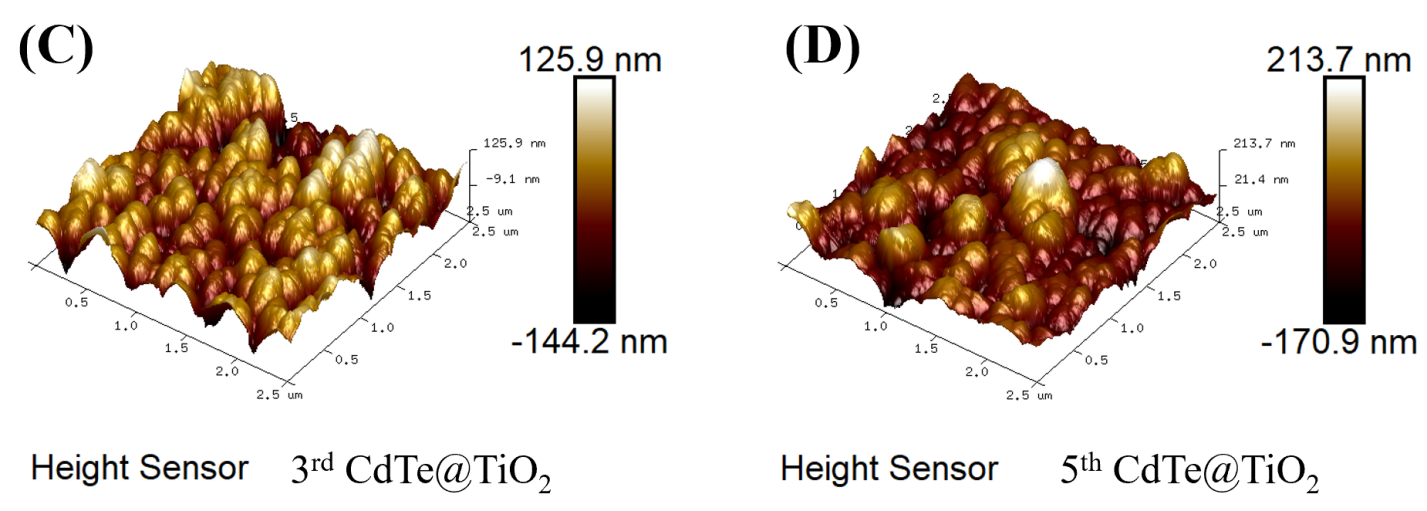


**Figure S25.** The AFM of topography on of (A) CdTe@TiO_2_ NTs, (B) 1^st^ CdTe@TiO_2_ NTs, (C) 3^rd^ CdTe@TiO_2_ NTs (D) and 5^th^ CdTe@TiO_2_ NTs film electrode


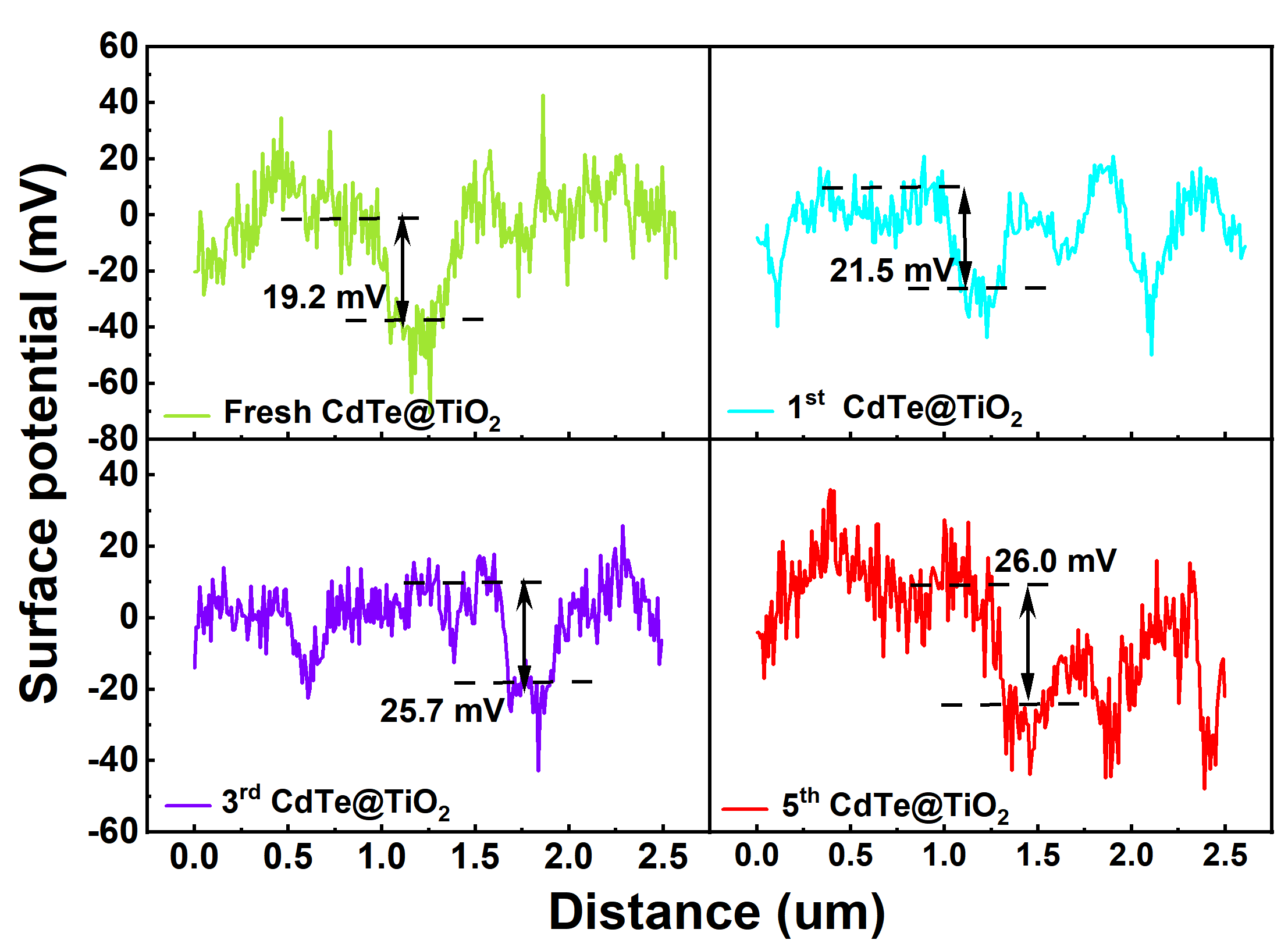


**Figure S26.** Extractive surface potentials for as-synthesized TiO_2_ NTs, CdTe@TiO_2_ NTs and recycled CdTe@TiO_2_ NTs


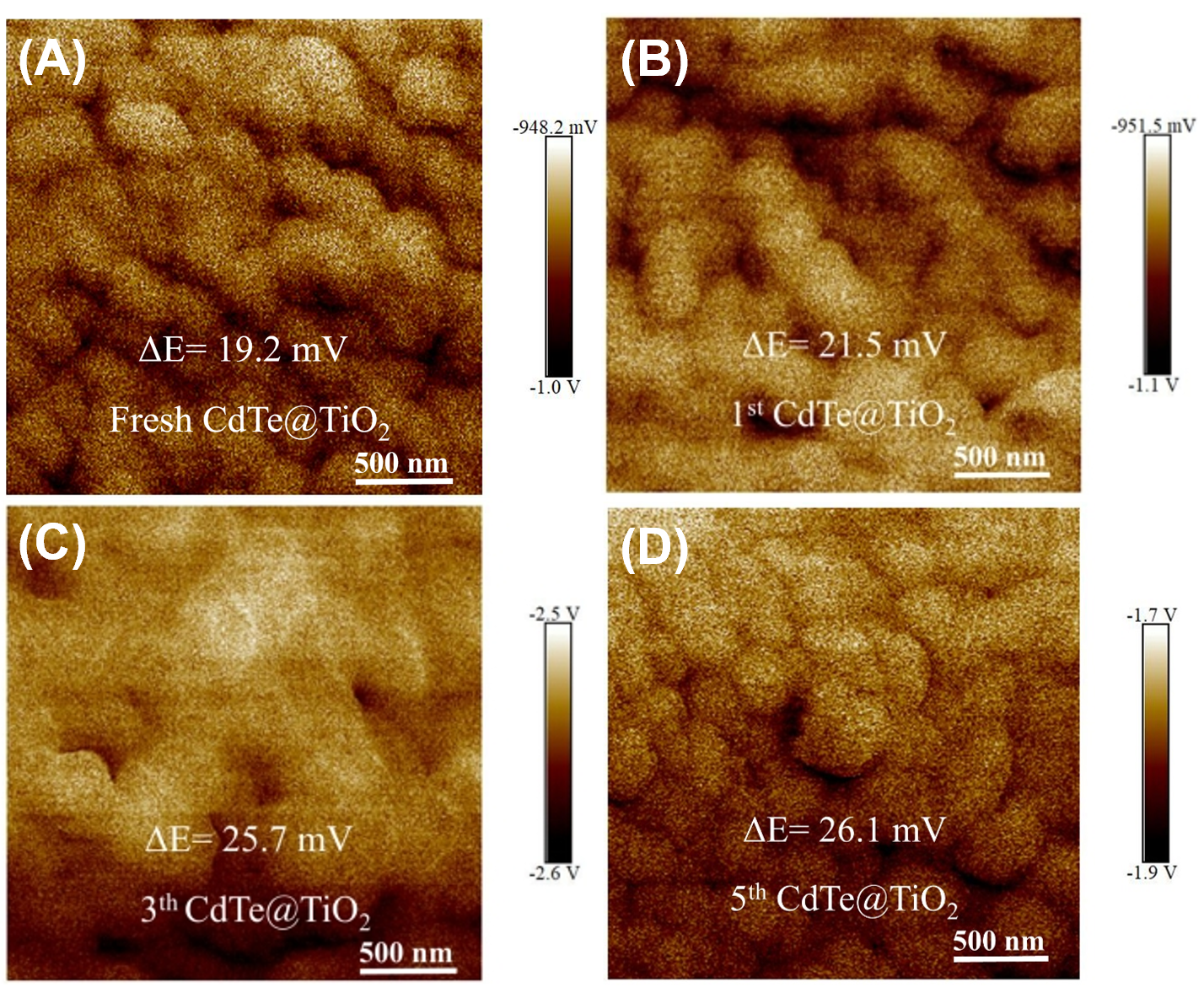


**Figure S27.** The surface potentials of (A) CdTe@TiO_2_ NTs, (B) 1^st^ CdTe@TiO_2_ NTs, (C) 3^rd^ CdTe@TiO_2_ NTs (D) and 5^th^ CdTe@TiO_2_ NTs film electrode measured with KPFM


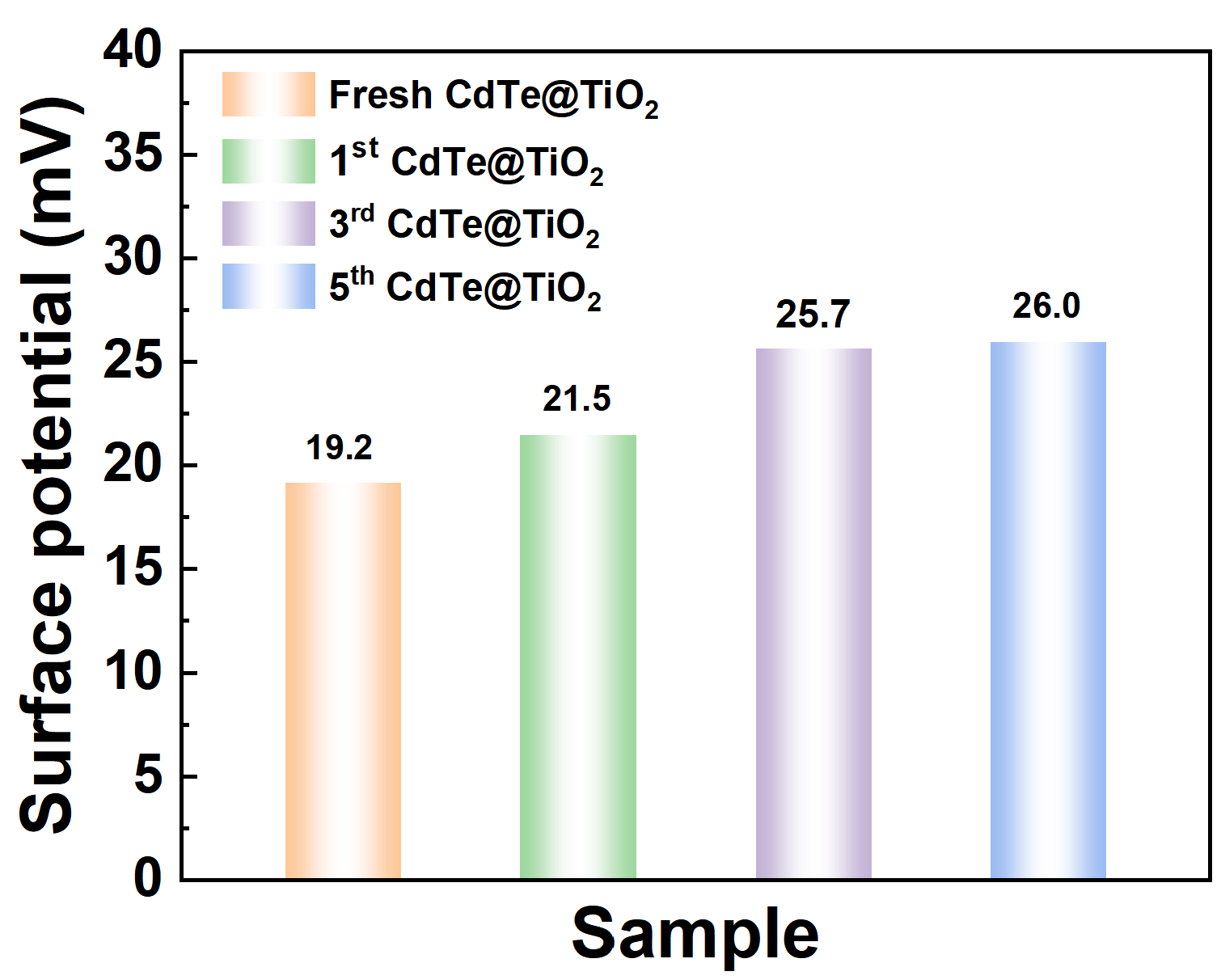


**Figure S28.** The estimated surface potential data extracted from the potential distribution: (A) CdTe@TiO_2_ NTs, (B) 1^st^ CdTe@TiO_2_ NTs, (C) 3^rd^ CdTe@TiO_2_ NTs and (D)5^th^ CdTe@TiO_2_ NTs measured with KPFM technology

**
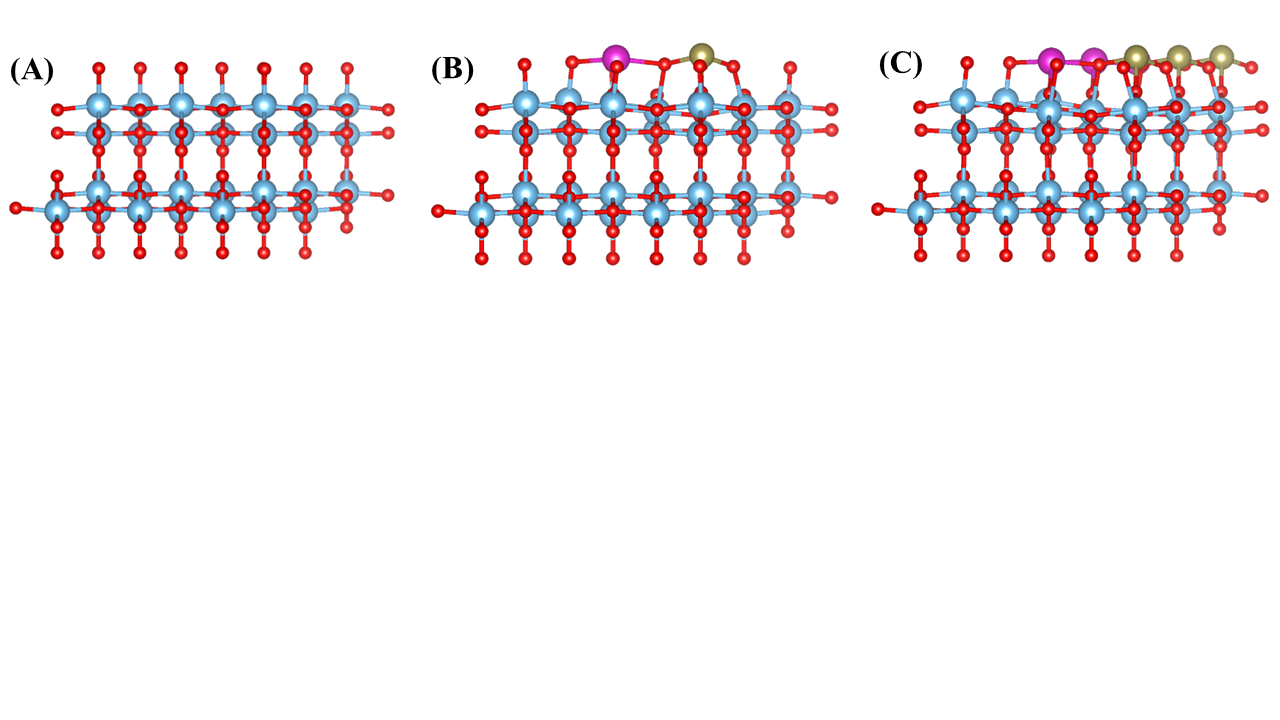
**

**Figure S29.** The optimized structure model in side view of (A) TiO_2_ NTs, (B) Cd_1_Te_1_@TiO_2_ NTs and (C) Cd_3_Te_3_@TiO_2_ NTs (Blue ball represents Ti atom; Red ball represents O atom; Purple ball represents Cd atom; Brown ball represents Te atom) (Cd_1_Te_1_@TiO_2_ NTs indicates that one Cd and Te atom is introduced in CdTe@TiO_2_ NTs; Cd_3_Te_3_@TiO_2_ NTs indicates that three Cd and Te atom are introduced in CdTe@TiO_2_ NTs)

_
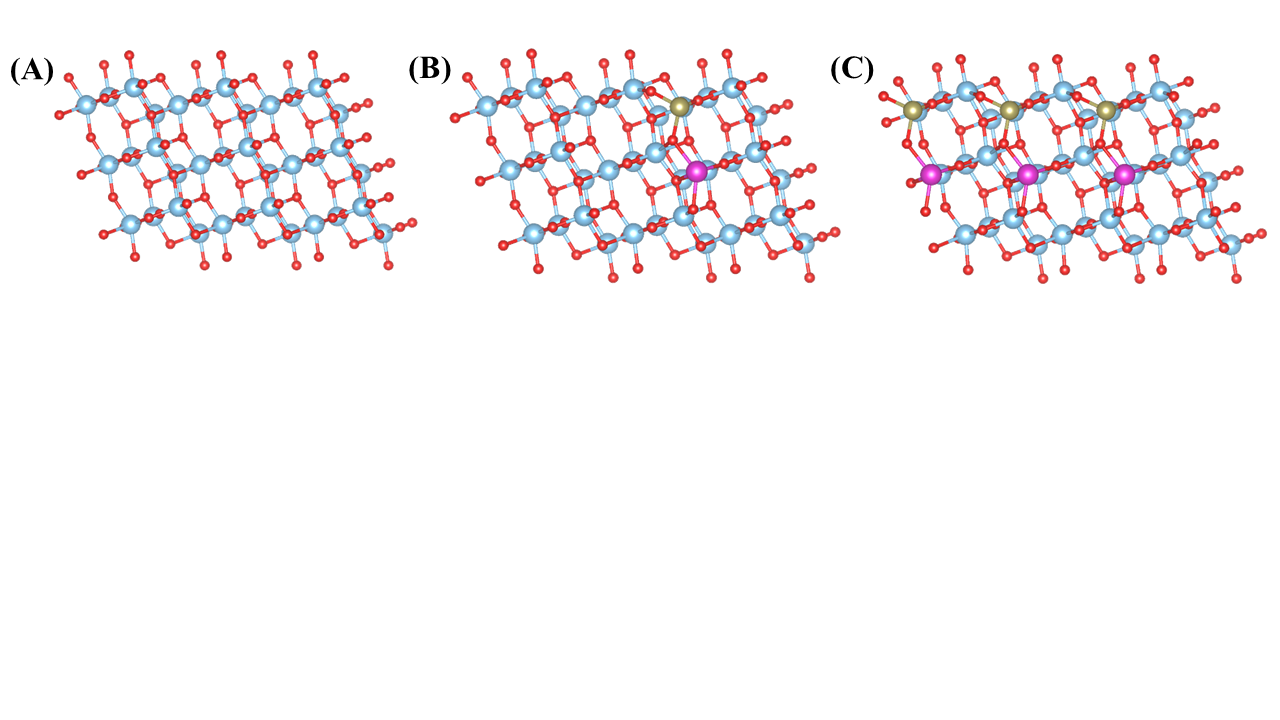
_

**Figure S30.** The optimized structure model in top view of (A) TiO_2_ NTs, (B) Cd_1_Te_1_@TiO_2_ NTs and (C) Cd_3_Te_3_@TiO_2_ NTs


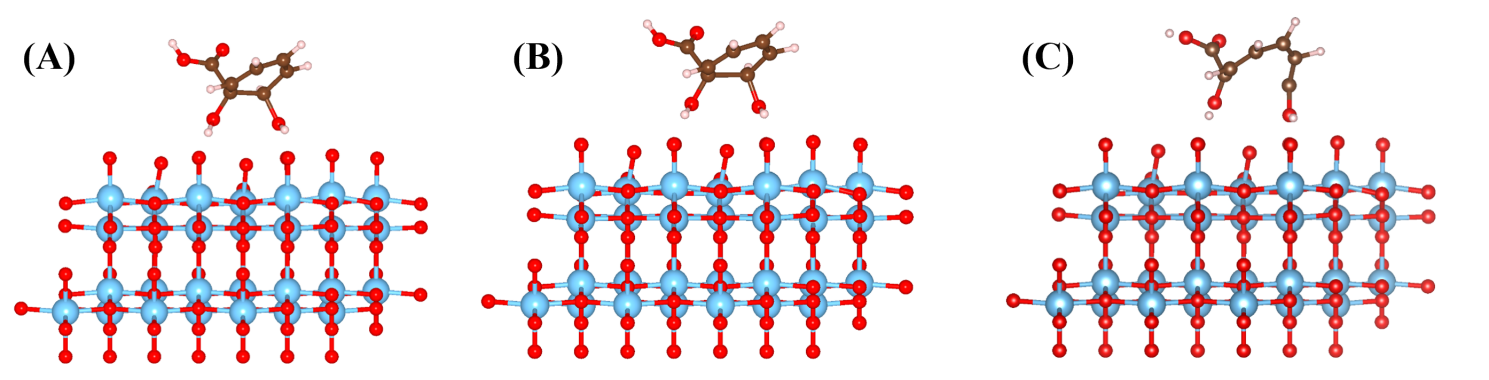


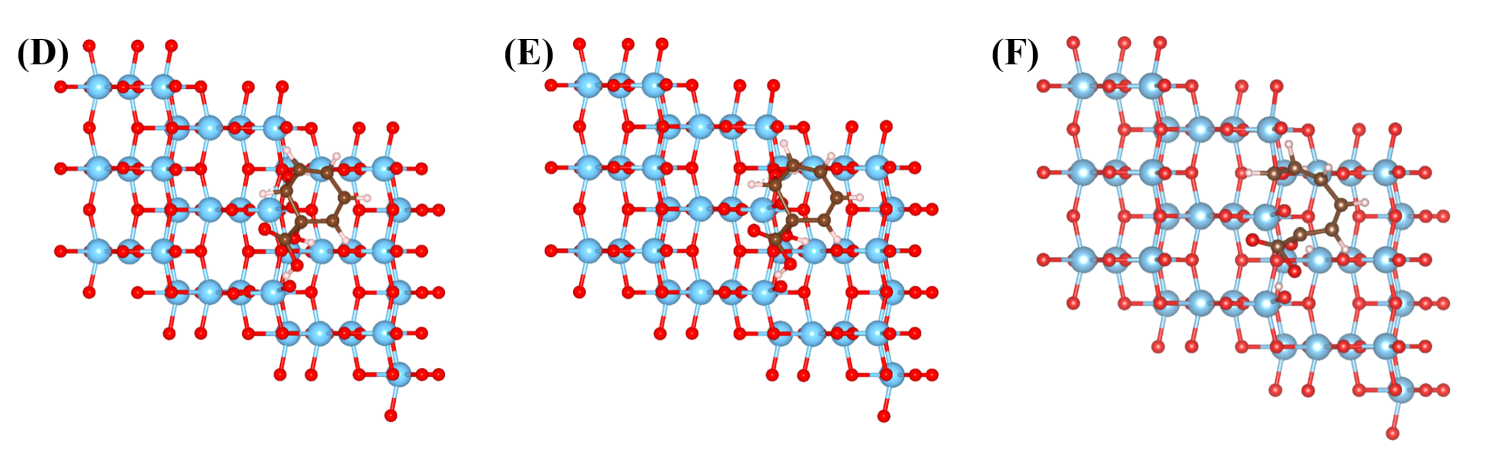


**Figure S31.** The optimized structure model of benzoic acid adsorbed in TiO_2_ NTs in side view for (A) IS, (B) TS and (C) FS; The optimized structure model of benzoic acid adsorbed in TiO_2_ NTs in top view for (D) IS, (E) TS and (F) FS (Blue ball represents Ti atom; Red ball represents O atom; Black ball represents C atom; White ball represents O atom)


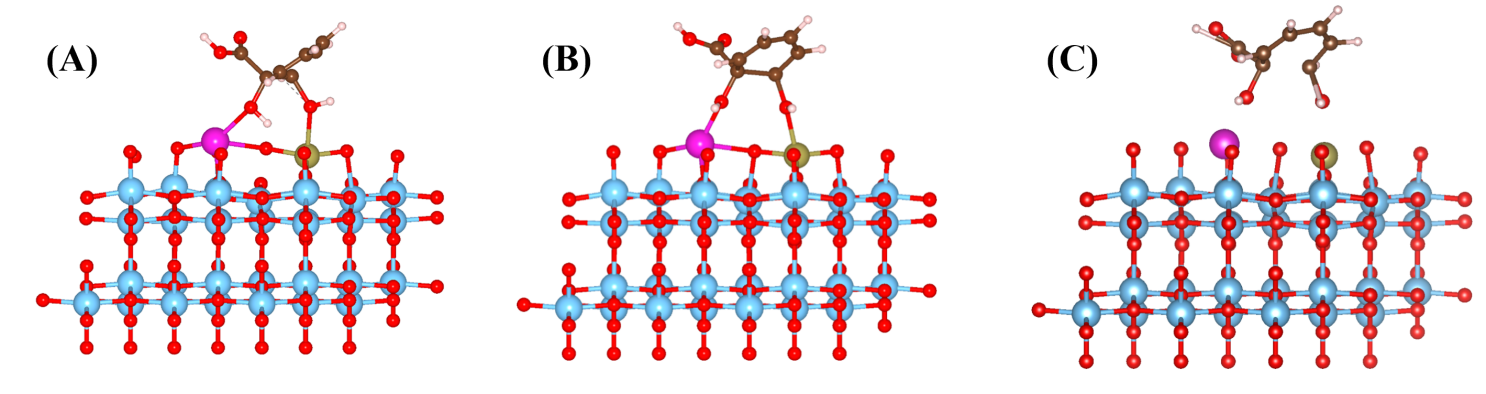


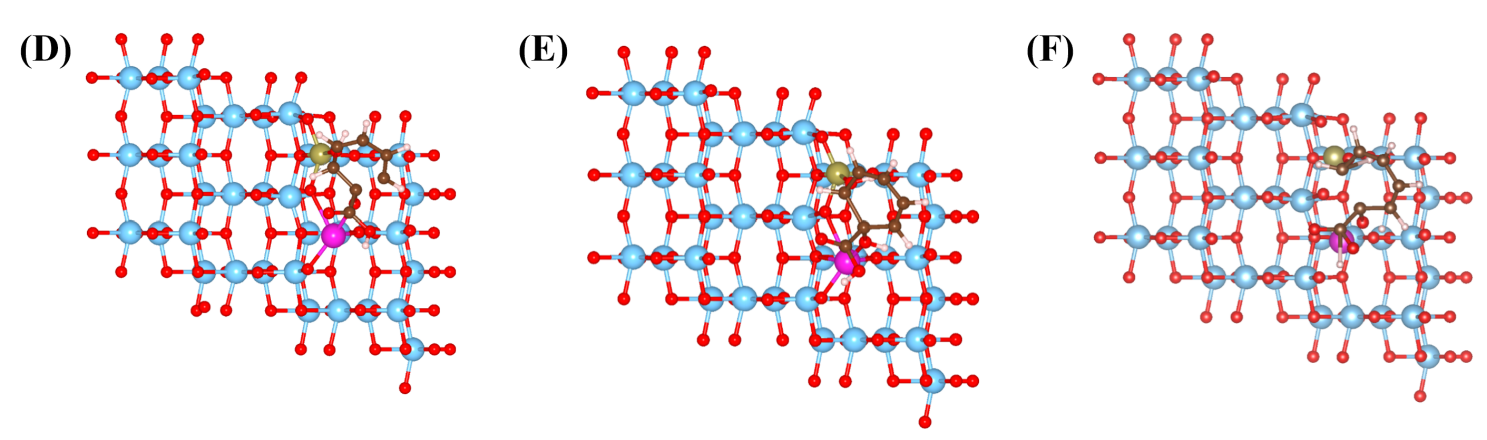


**Figure S32.** The optimized structure model of benzoic acid adsorbed in Cd_1_Te_1_@TiO_2_ NTs in side view for (A) IS, (B) TS and (C) FS; The optimized structure model of benzoic acid adsorbed in TiO_2_ NTs in top view for (D) IS, (E) TS and (F) FS


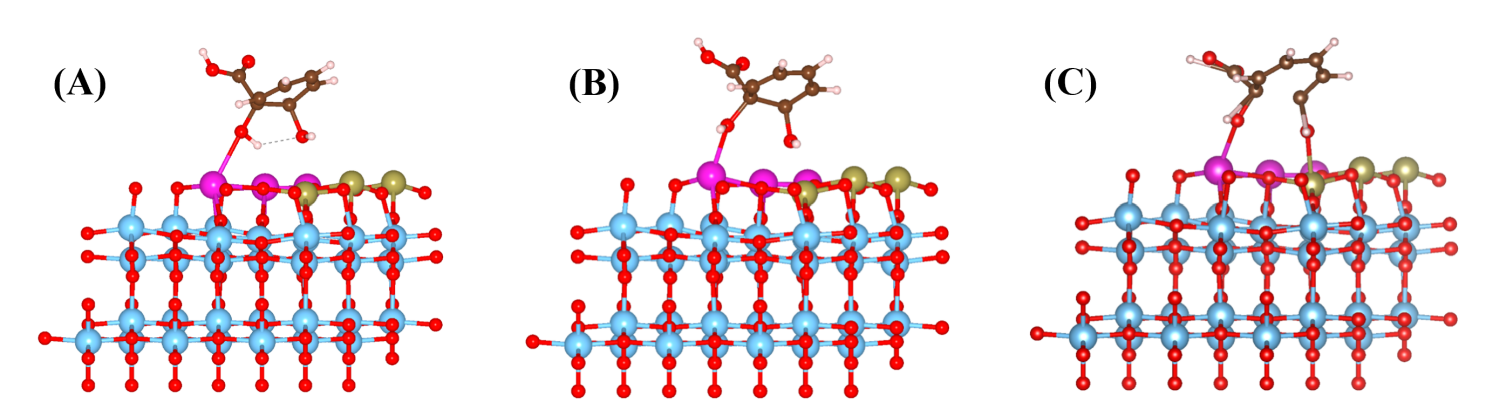


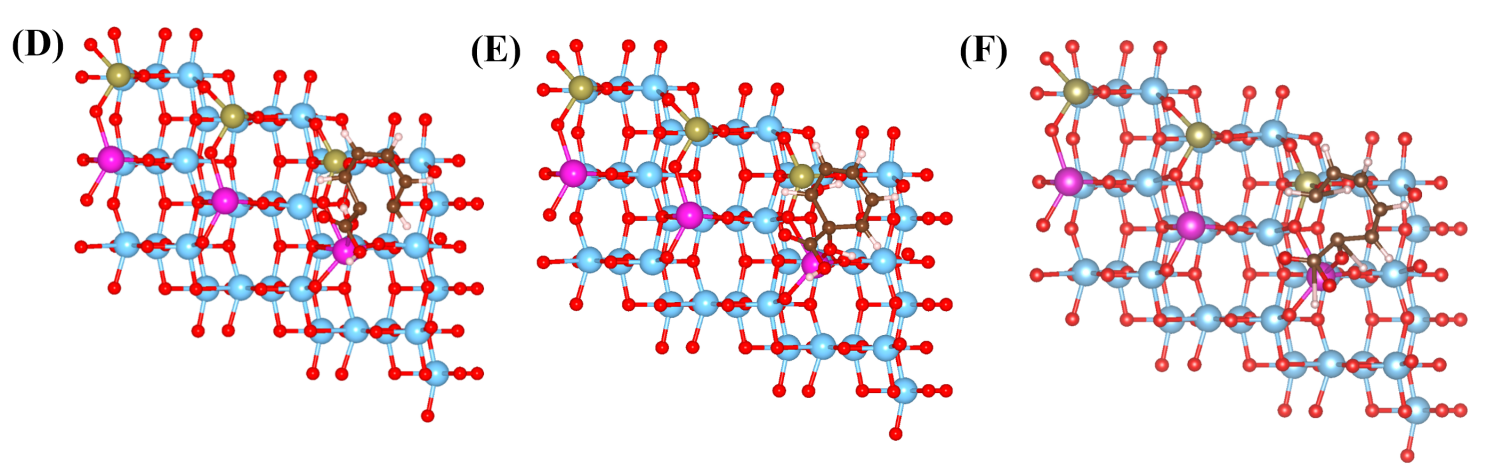


**Figure S33.** The optimized structure model of benzoic acid adsorbed in Cd_3_Te_3_@TiO_2_ NTs in side view for (A) IS, (B) TS and (C) FS; The optimized structure model of benzoic acid adsorbed in TiO_2_ NTs in top view for (D) IS, (E) TS and (F) FS


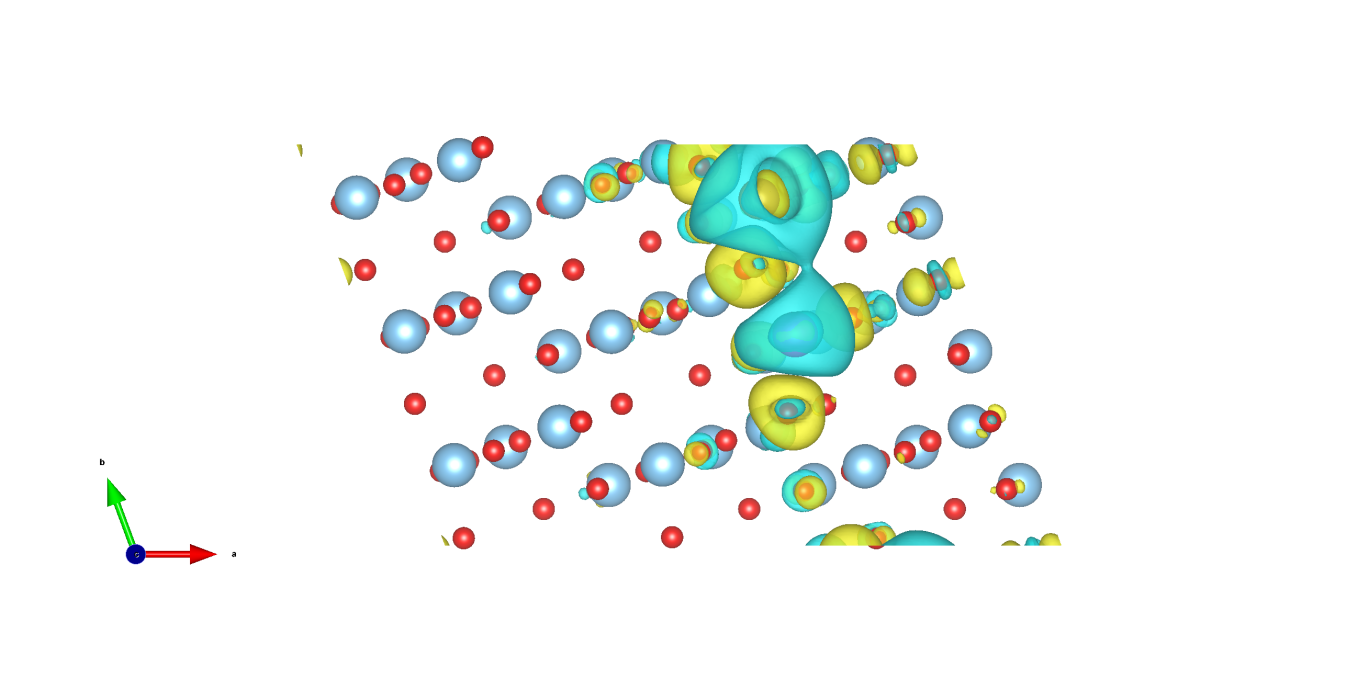


**Figure S34.** Charge density distribution of Cd_1_Te_1_@TiO_2_ NTs in top view with 0.002 e Å^-3^ (Yellow and cyan isosurfaces denote electron accumulation and depletion regions, respectively)


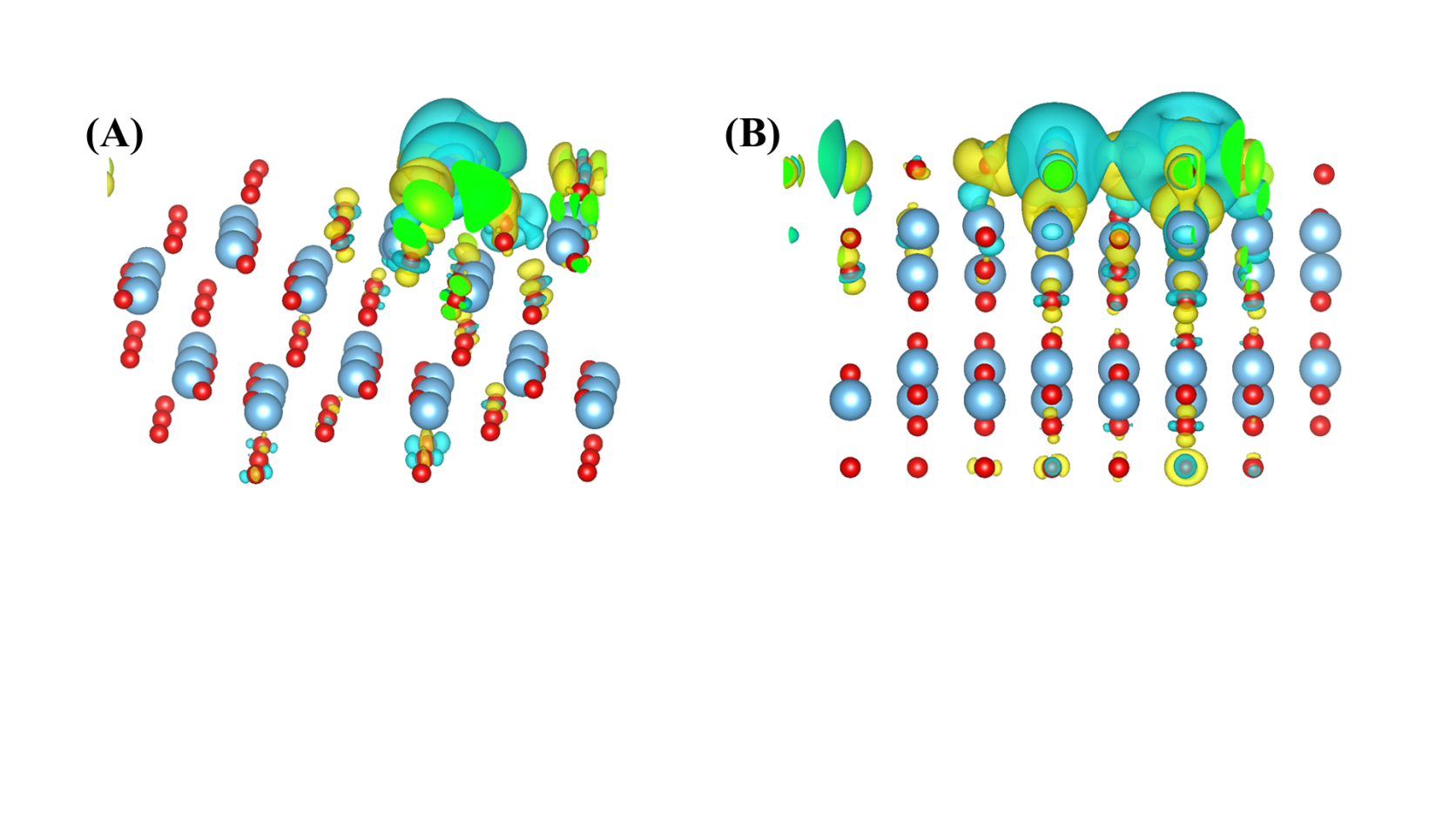


**Figure S35.** Charge density distribution of Cd_1_Te_1_@TiO_2_ NTs in side view with 0.002 e Å^-3^


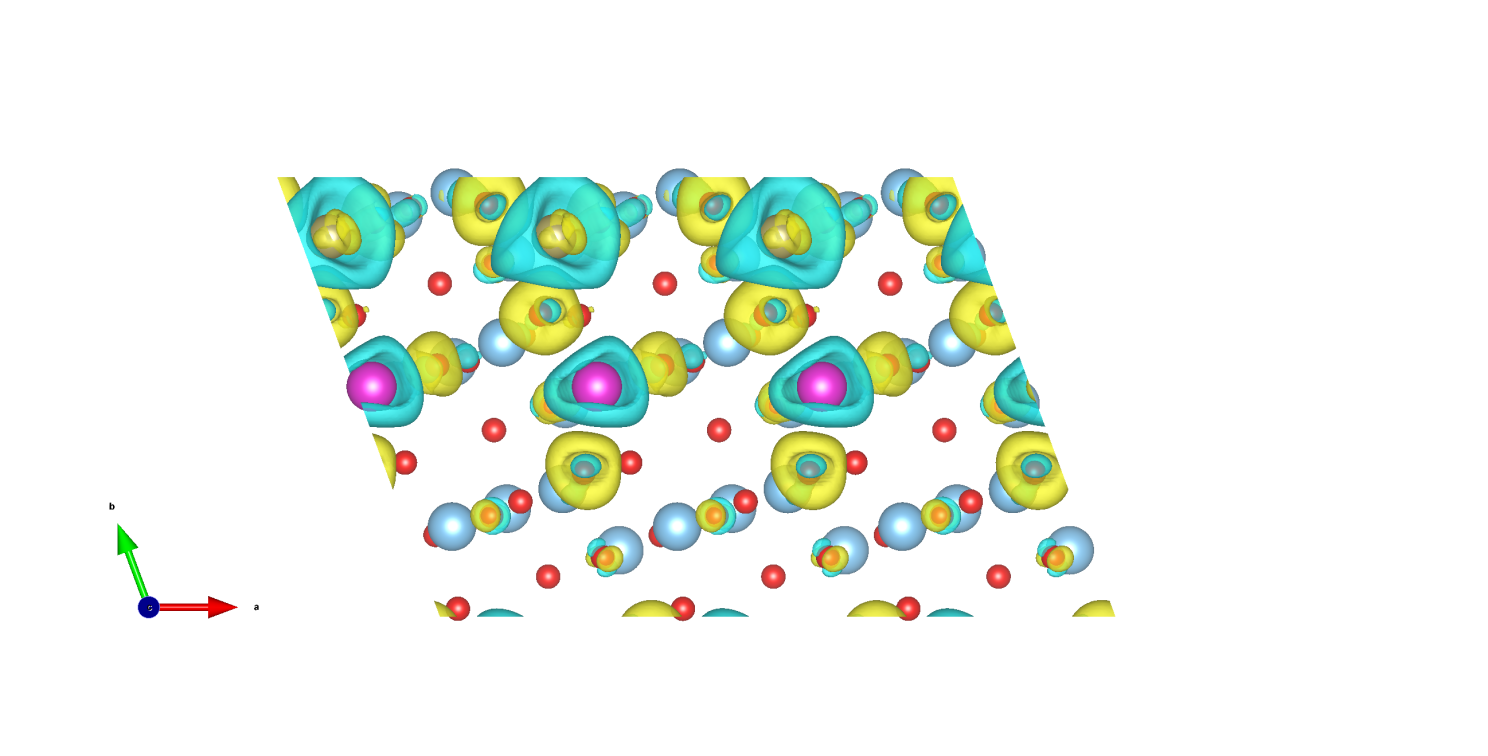


**Figure S36.** Charge density distribution of Cd_3_Te_3_@TiO_2_ NTs in top view with 0.002 e Å^-3^


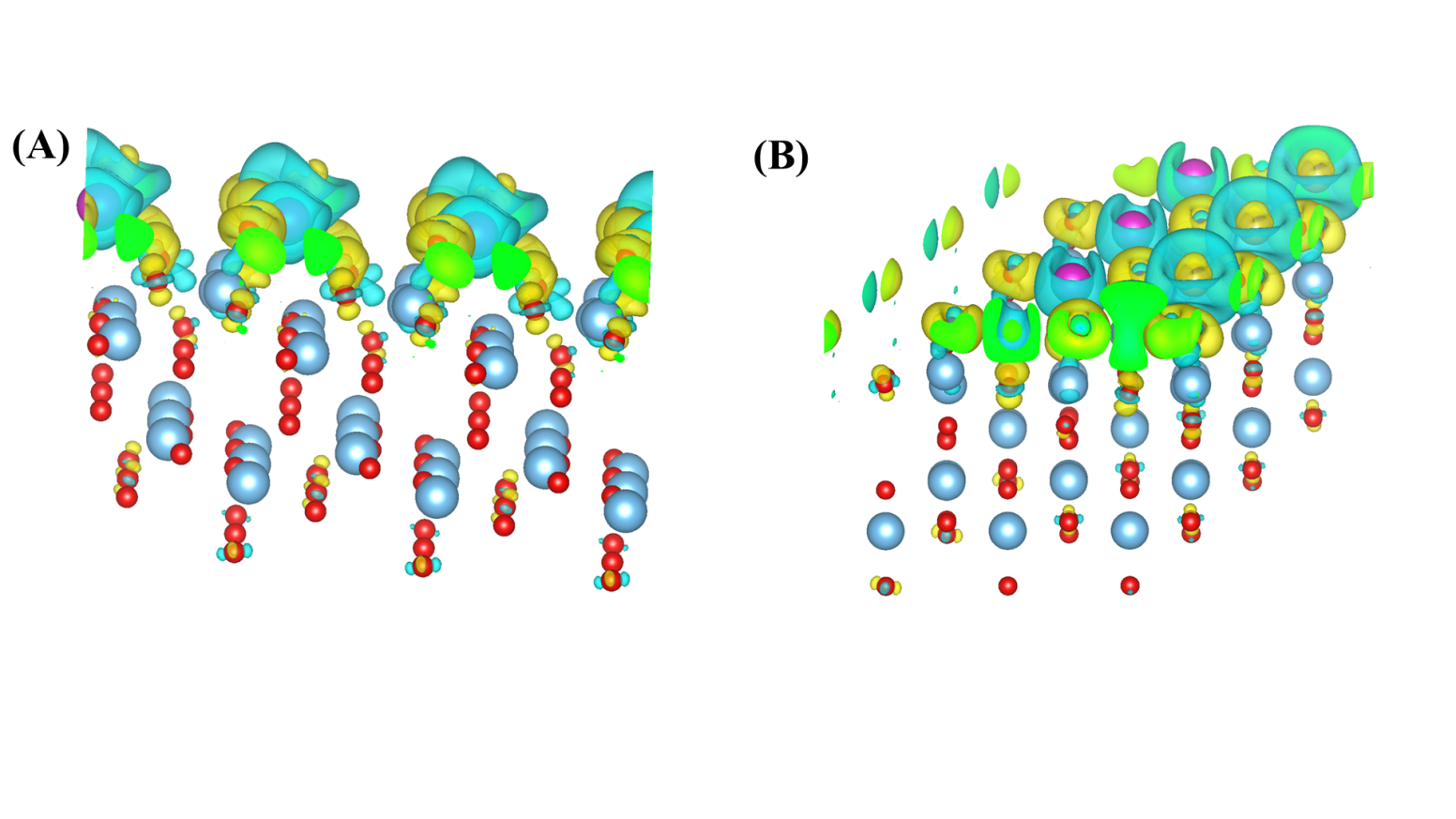


**Figure S37.** Charge density distribution of Cd_3_Te_3_@TiO_2_ NTs in side view with 0.002 e Å^-3^


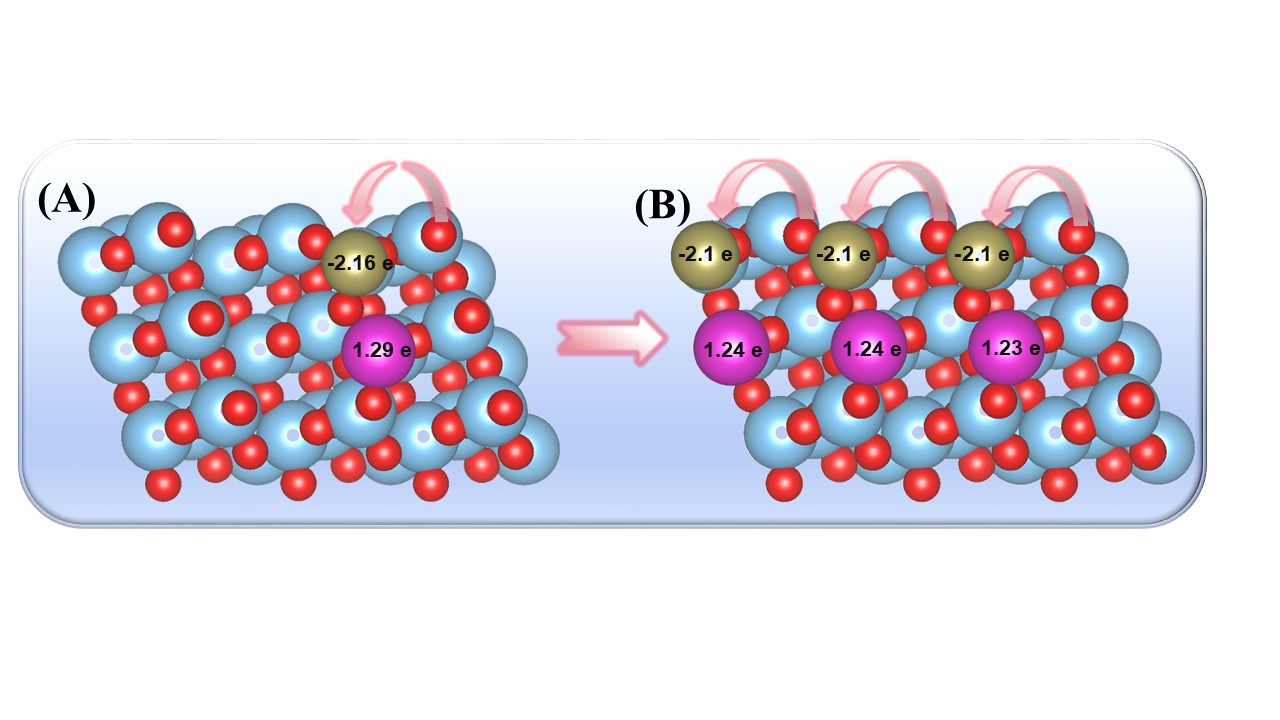


**Figure S38.** Bader charge distribution analysis on (A) Cd_1_Te_1_@TiO_2_ NTs and (B) Cd_3_Te_3_@TiO_2_ NTs


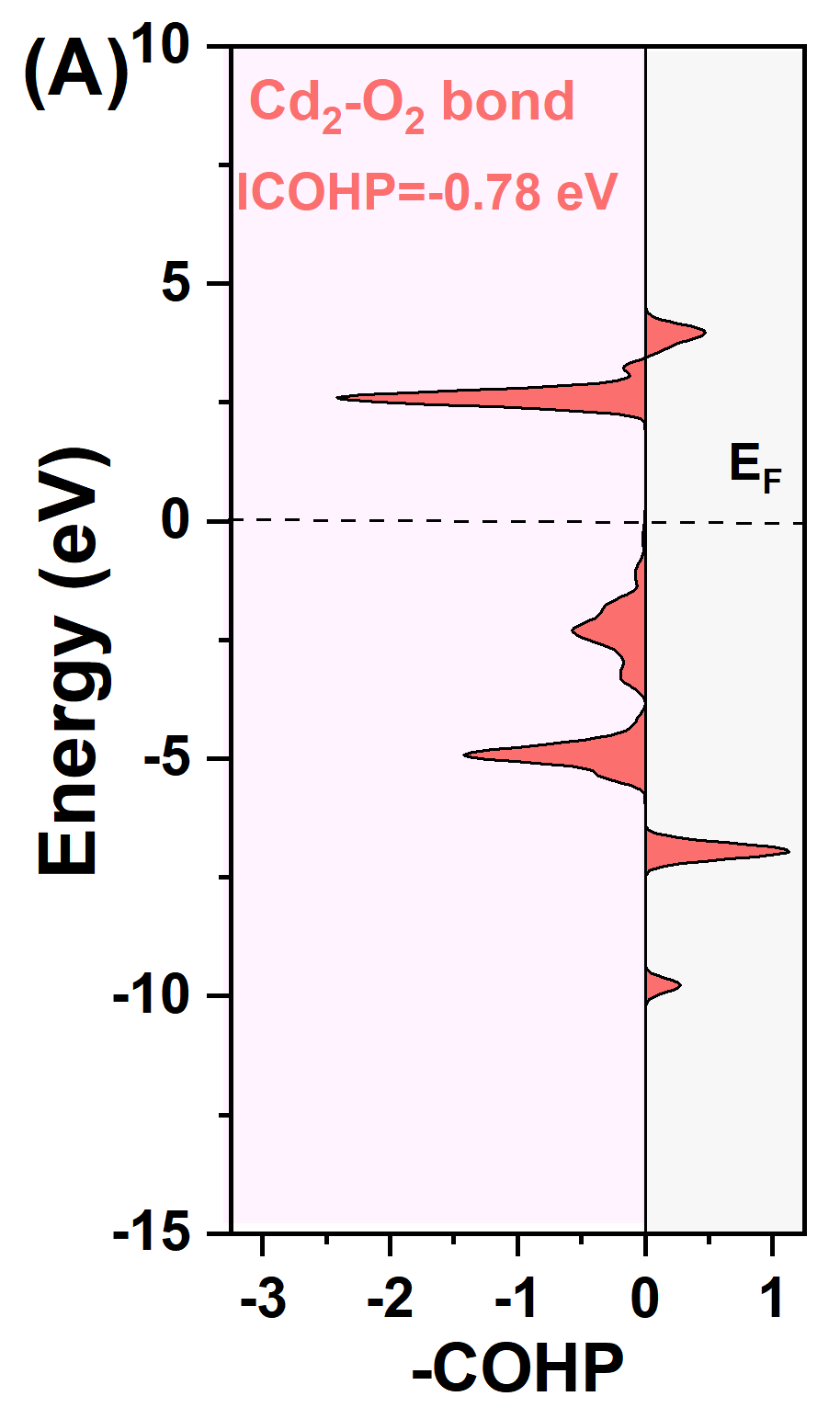

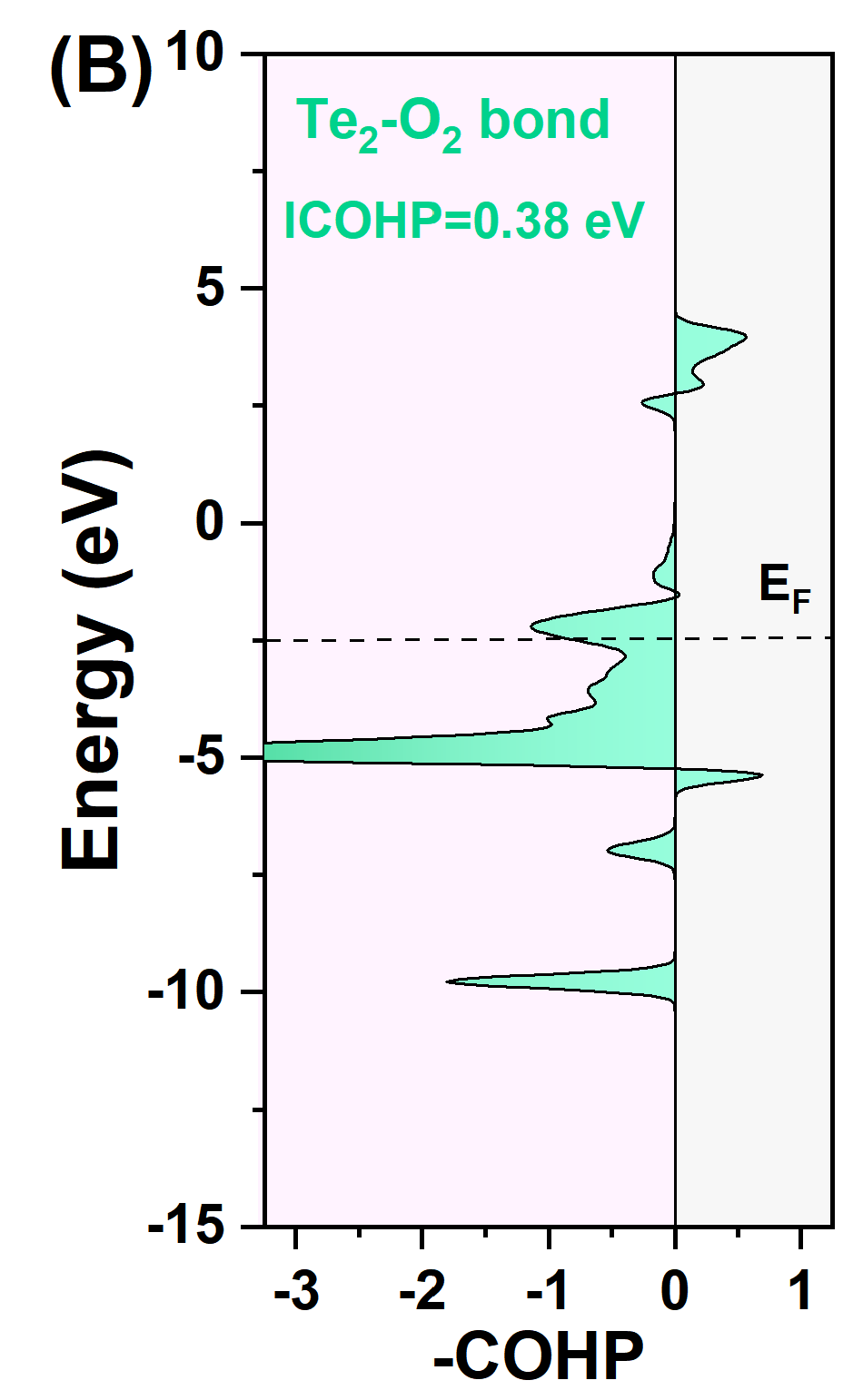


**Figure S39.** Projected crystal orbital Hamilton population (COHP) for (A) the Cd_2_-O_2_ and (B) Te_2_-O_2_ bond in Cd_3_Te_3_@TiO_2_ NTs


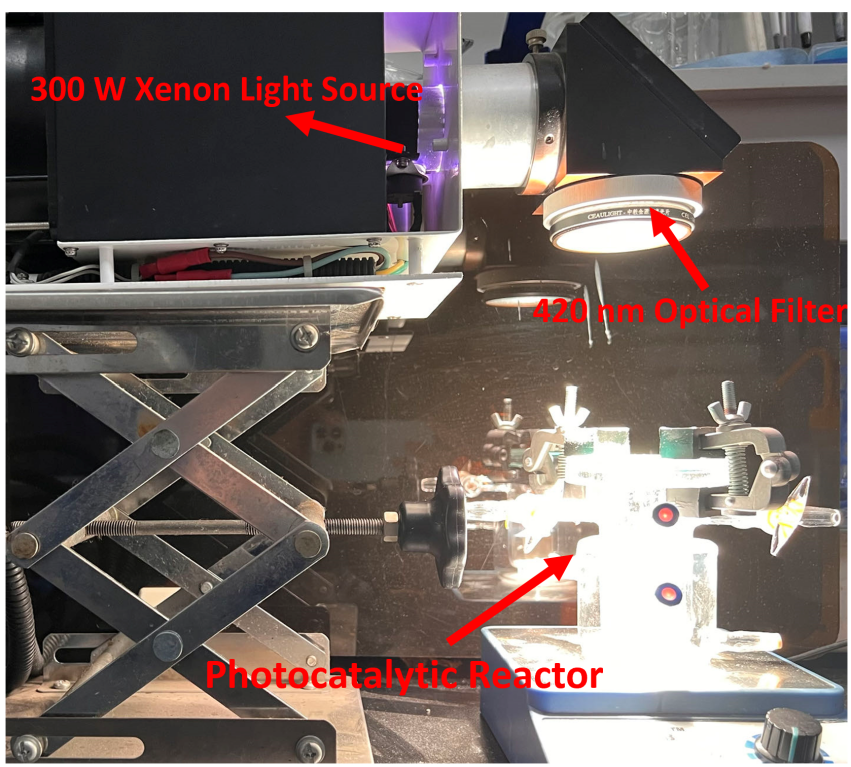


**Figure S40.** Photocatalytic performance test device

**Table S1.** Charge transfer resistances

| **Resistance** | **Fresh** | **1st** | **3rd** | **5th** |
| --- | --- | --- | --- | --- |
| **R_s_ (ohm.cm^2^)** | 25.68 | 23.23 | 18.98 | 18.06 |
| **R_ct_ (kohm.cm^2^)** | 32.16 | 26.89 | 23.56 | 21.18 |

**Reference:**

[S1] G. Kresse, J. Furthmüller, Efficiency of ab-initio total energy calculations for metals and semiconductors using a plane-wave basis set. Comp. Mater. Sci. **6**, 15-50, (1996).

[S2] P.E. Blo ̈chl, O. Jepsen, O.K. Andersen, Improved tetrahedron method for brillouin-zone integrations, Phys. Rev. B Condens. Matter. **49**, 16223-16233 (1994).

[S3] J.P. Perdew, J.A. Chevary, S.H. Vosko, K.A. Jackson, M.R. Pederson, D.J. Singh, C. Fiolhais, Erratum: atoms, molecules, solids, and surfaces: applications of the generalized gradient approximation for exchange and correlation. Phys. Rev. B Condens. Matter. **48**, 4978-4978 (1993).

[S4] Grimme S, Antony J, Ehrlich S and Krieg H, A consistent and accurate ab initio parametrization of density functional dispersion correction (DFT-D) for the 94 elements H-Pu. Journal of Chemical Physics. **132**, 154104 (2010).

[S5] Henkelman G, Uberuaga B P, Jónsson H, A climbing image nudged elastic band method for finding saddle points and minimum energy paths. The Journal of chemical physics, 113, 9901-9904 (2000).
